# Annulation of *O*-silyl *N*,*O*-ketene acetals with alkynes for the synthesis of dihydropyridinones and its application in concise total synthesis of phenanthroindolizidine alkaloids

**DOI:** 10.3389/fchem.2023.1267422

**Published:** 2023-09-21

**Authors:** Seokwoo Lee, Jae Eui Shin, Ran Yoon, Hanbin Yoo, Sanghee Kim

**Affiliations:** ^1^ College of Pharmacy, Seoul National University, Seoul, Republic of Korea; ^2^ College of Pharmacy, Chungnam National University, Daejeon, Republic of Korea

**Keywords:** *N*-heterocycle, *O*-silyl, *N*,*O*-ketene acetal, dihydropyridinone, total synthesis, phenanthroindolizidine, phenanthroquinolizidine, alkaloid

## Abstract

The formation of *N*-heterocycles with multiple substituents is important in organic synthesis. Herein, we report a novel method for the construction of functionalized dihydropyridinone rings through the annulation of an amide *α*-carbon with a tethered alkyne moiety. The reaction of the amide with the alkyne was achieved via *O*-silyl *N*,*O*-ketene acetal formation and silver-mediated addition. Furthermore, the developed method was applied for the total synthesis of phenanthroindolizidine and phenanthroquinolizidine alkaloids. By varying the coupling partners, a concise and collective total synthesis of these alkaloids was achieved.

## 1 Introduction

**SCHEME 1:**
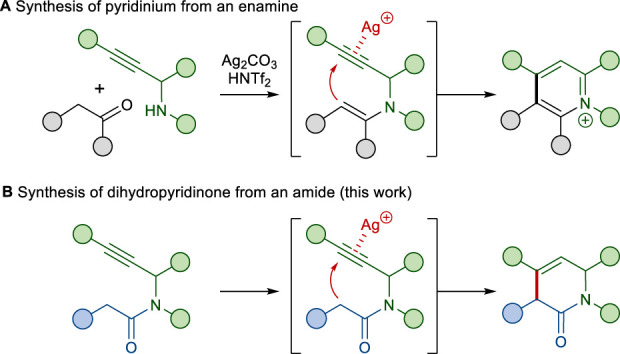
Synthesis of *N*-heterocycles via the annulation of an alkyne with a tethered moiety.

The construction of *N*-heterocycles containing multiple substituents still remains an important synthetic challenge (Vo et al., 2014; Nandakumar et al., 2015; He et al., 2021; Chen et al., 2023). We recently described the Ag(I) and Brønsted acid co-catalyzed cyclization of an enamine with a tethered alkyne moiety as a one-pot method for pyridinium formation (Scheme 1A) (Lee et al., 2023). Our proposed mechanism for the transformation involves the addition of a nucleophilic enamine to a silver(I)-complexed alkyne, followed by protonolysis of the resulting vinyl-silver species and subsequent aromatization. Based on this annulation, we envisioned that the nucleophilic addition of the amide *α*-carbon onto the appended alkyne would form a dihydropyridinone (Scheme 1B). To the best of our knowledge, the reactions of alkynes with amides have not been well explored, although reactions with various types of carbon nucleophiles, especially stabilized carbon nucleophiles such as malonates, *β*-ketoesters, and diketones, have been well explored (Dénes et al., 2010; Hack et al., 2015; Lin et al., 2021).

Herein, we report the annulation of an amide *α*-carbon with a tethered alkyne moiety, which is a new complementary process for the functionalization of dihydropyridinone rings. In addition, we discuss the application of this C–C bond-forming reaction for the expedient total synthesis of phenanthroindolizidine and phenanthroquinolizidine alkaloids.

## 2 Results and discussion

We examined the feasibility of the proposed reaction using model substrate **1** (Table 1), which was prepared in two steps from commercially available materials. Based on our previous results on pyridinium formation (Lee et al., 2023), Ag_2_CO_3_ or AgNTf_2_ were employed as a catalyst for alkyne activation. Without a base, no conversion of **1** occurred. The addition of a conventional base, such as an alkali metal carbonate or 1,8-diazabicyclo[5.4.0]undec-7-ene (DBU), did not result in product formation (Supplementary Table S1). Despite the reported incompatibility of strong bases and Lewis-acidic metals (Yamamoto, 2000; Yamamoto et al., 2008; Lappert et al., 2009), the strong bases generally used for amide enolate generation, including potassium bis(trimethylsilyl)amide (KHMDS) and LiHMDS, were also examined. However, all of these attempts failed, and most of the starting material decomposed or was recovered (Supplementary Table S1).

**TABLE 1:** Conditions for the formation of **2** **from** **1**.**^a^**

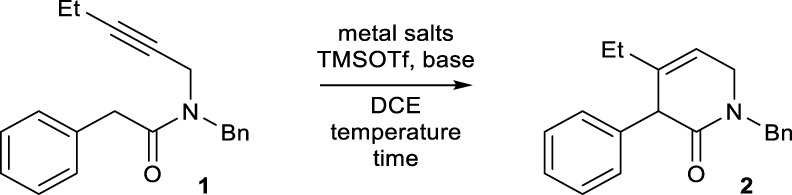					

Entry	Metal salts	Base	Temp.	Time (h)	Yield (%)^b^
1	Ag_2_CO_3_	DIPEA	reflux	1	59 (57)^c^
2	Ag_2_CO_3_	2,6-lutidine	reflux	1	46
3	Ag_2_CO_3_	DIPEA	60°C	1	63
4	Ag_2_CO_3_	DIPEA	40°C	1.5	80
5	Ag_2_CO_3_	DIPEA	r.t.	6	95 (93)^c^
6	AgNTf_2_	DIPEA	r.t.	3	96 (93)^c^
7^d^	AgNTf_2_	DIPEA	r.t.	3	95 (92)^c^
8^e^	AgNTf_2_	DIPEA	r.t.	3	94 (90)^c^

^a^ Reaction conditions: **1** (0.1 mmol), metal salts (0.1 equiv), TMSOTf (4.0 equiv), base (4.0 equiv), DCE (0.05 M).

^b^ The chemical yield was estimated via ^1^H NMR, analysis of the crude reaction mixtures using tetrachloroethane (C_2_H_2_Cl_4_) as the internal standard.

^c^ Isolation yield.

^d^ The reaction was carried out under CH_2_Cl_2_.

^e^ 1.0 mmol scale. TMSOTf = Trimethylsilyl trifluoromethanesulfonate, DIPEA = *N*,*N*-Diisopropylethylamine.

We turned our attention to an *O*-silyl *N*,*O*-ketene acetal as a surrogate for the amide enolate. *O*-Silyl *N*,*O*-ketene acetals have been typically used for Mukaiyama-type reactions (Paris et al., 2022) and generated *in situ* by the treatment of an amide with a silylating agent and tertiary amine base (Kobayashi et al., 2011; Downey et al., 2015; Takeda et al., 2017). Previously, Shen and coworkers reported the gold(I)-catalyzed cyclization of alkynes with an *O*-silyl ketene amide or carbamate nucleophiles (Minnihan et al., 2007). However, the reaction of an alkyne with a silyl *N*,*O*-ketene acetal has not been reported.

At the outset of this study, TMSOTf was used as a silylating agent, and various amine bases were screened in the presence of 0.1 equiv of Ag_2_CO_3_ in dichloroethane (DCE) under reflux conditions. Among the tested amine bases, *N*,*N*-diisopropylethylamine (DIPEA) exhibited the best performance, affording dihydropyridinone **2** in a modest 59% yield along with a mixture of unidentifiable polar side products (Table 1, entry 1). The other sterically hindered base 2,6-lutidine also provided **2**, albeit in a lower yield (46%, entry 2).

After determining the feasibility of the reaction, further screening of the reaction conditions was performed using DIPEA and TMSOTf. Under reflux conditions, the yield of compound **2** was reduced, likely due to the formation of considerable amounts of unidentified side products. A reduction in the reaction temperature led to an increase in the yield of compound **2** (entries 3–5), likely as a result of decreased formation of side products. For example, **2** was formed in an excellent yield of 95% at room temperature, although a longer reaction time was required (entry 5). When Ag_2_CO_3_ was replaced with AgNTf_2_, the reaction time was reduced by half (3 h), and the yield was also excellent (96%, entry 6). As in our previous study on the annulation of enamines with alkynes (Lee et al., 2023), the 5-membered heterocycles formed via 5-*exo*-*dig* cyclization were not observed under the conditions. Other silylating agents did not lead to better yields than TMSOTf (Supplementary Table S2). Several solvents were tested for this transformation. The only other effective solvent was CH_2_Cl_2_, which furnished **2** with a similar yield (95%, entry 7). Other solvents did not enable the formation of **2** (Supplementary Table S3). Under the optimal conditions, the reaction could be enlarged to a 1.0 mmol scale without a significant decrease in yield (94%, entry 8).

**FIGURE 1:**
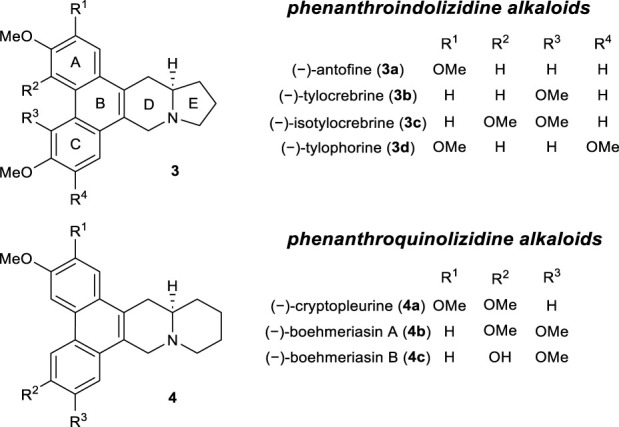
The structures of representative phenanthroindolizidine and phenanthroquinolizidine alkaloids.

Based on these results, we attempted the total synthesis of phenanthroindolizidine alkaloids (Figure 1). This family of natural products exhibits a wide range of biological effects, including significant anticancer and antiviral activities (De Fatima Pereira et al., 2015; Jia et al., 2021). Therefore, these alkaloids have been the synthetic targets of numerous research groups over the past few decades (Chemler, 2009; Burtoloso et al., 2014).

**SCHEME 2:**
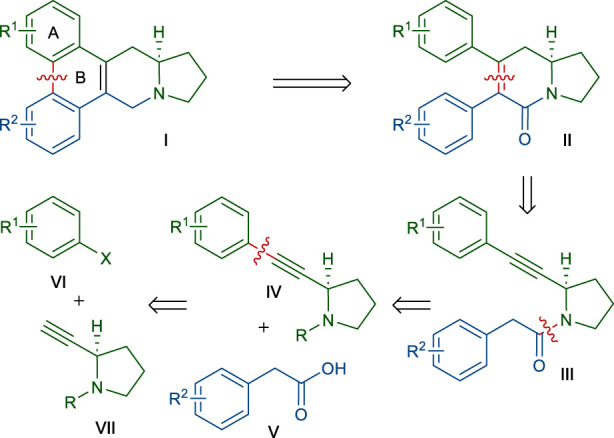
Retrosynthetic route for the synthesis of phenanthroindolizidine alkaloids.

Our retrosynthetic analysis, based on the above dihydropyridinone synthetic strategy, is depicted in Scheme 2. The B ring of the phenanthroindolizidine skeleton of **Ⅰ** could be constructed at the last stage of the synthesis via the biaryl coupling of **Ⅱ**. We envisioned that dihydropyridinone ring of **Ⅱ** could be formed by the annulation of an amide *α*-carbon with a tethered alkyne moiety in **Ⅲ**, according to the above-mentioned method. An obvious disconnection of the amide bond in **Ⅲ** led to the 2-alkyne-pyrrolidine **Ⅳ** and 2-arylacetic acid **Ⅴ**. Pyrrolidine derivative **Ⅳ** would be accessed by coupling of an aryl halide **Ⅵ** with the known alkyne **Ⅶ**. According to this retrosynthetic scheme, many members of this phenanthroindolizidine family and analogs could be synthesized by varying the two coupling partners **Ⅴ** and **Ⅵ**. Even, this scheme would permit the synthesis of phenanthroquinolizidine alkaloids if 2-alkyne-piperidine was employed instead of **Ⅶ**.

**SCHEME 3:**
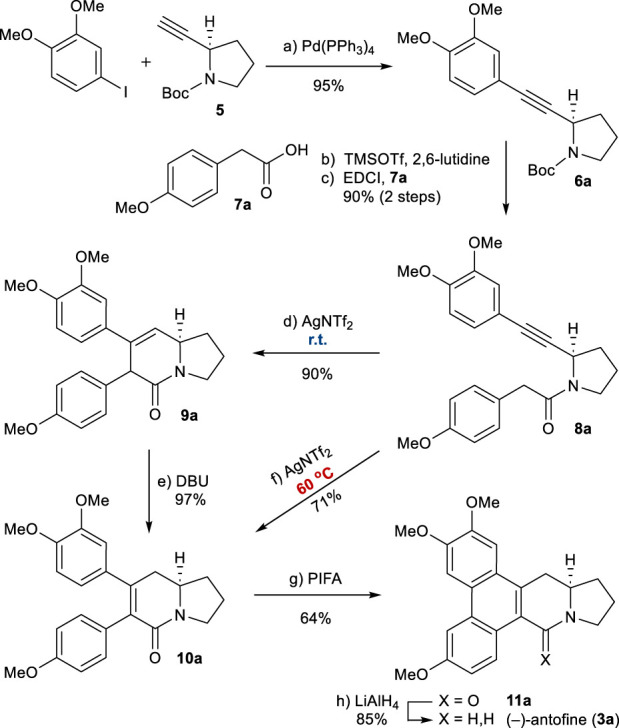
Synthesis of (−)-antofine (**3a**). Reagents and conditions: a) Pd(PPh_3_)_4_ (0.05 equiv), piperidine/MeCN (1:1), reflux, 3 h, 95%; b) TMSOTf (2.0 equiv), 2,6-lutidine (3.0 equiv), CH_2_Cl_2_, 0°C, 10 min; c) **7a** (1.2 equiv), EDCI (1.1 equiv), DMAP (1.1 equiv), CH_2_Cl_2_, r.t., 12 h, 90% for 2 steps; d) AgNTf_2_ (0.1 equiv), TMSOTf (4.0 equiv), DIPEA (4.0 equiv), DCE, r.t., 1 h, 90%; e) DBU (4.0 equiv), toluene, 90°C, 30 min, 97%; f) AgNTf_2_ (0.1 equiv), TMSOTf (4.0 equiv), DIPEA (4.0 equiv), DCE, 60°C, 2 h, 71%; g) PIFA (1.1 equiv), BF_3_OEt_2_ (1.5 equiv), CH_2_Cl_2_, –10°C, 1 h, 64%; h) LiAlH_4_ (2.0 equiv), THF, reflux, 1 h, 85%. EDCI = *N*-Ethylcarbodiimide hydrochloride, DMAP = 4-Dimethylaminopyridine.

Our synthesis began with the preparation of known alkyne **5** (Scheme 3), which is available in two steps from commercially available *N*-Boc-d-prolinol (Mercado-Marin et al., 2014). The Sonogashira coupling of **5** with 3,4-dimethoxy phenyl iodide afforded **6a** in high yield. Removal of the *N*-Boc group, followed by EDCI-mediated coupling with 2-arylacetic acid **7a**, generated amide **8a** in a good overall yield. Application of the developed reaction conditions to **8a** was successful, resulting in the formation of 3,6-dihydropyridin-2-one **9a** as the major product (90%) after 1 h. Treatment with DBU at 90°C led to the isomerization of **9a** to the thermodynamically more favorable 5,6-dihydropyridinone **10a** (see Supplementary Material). At this stage, we envisaged that **10a** could be obtained directly from the alkyne–amide cyclization. Fortunately, we found modified conditions that allowed the direct formation of **10a** from **8a**. At an elevated temperature of 60°C for 2 h, **10a** was obtained directly from **8a** in a 71% yield.

The oxidative biaryl coupling of **10a** was accomplished with hypervalent iodine reagent phenyliodine(III) bis(trifluoroacetate) (PIFA) to give pentacyclic product **11a** in a 64% yield (Kwon et al., 2015). Finally, the amide group of **11a** was reduced with LiAlH_4_ to give (−)-antofine (**3a**) in an 85% yield (Iwao et al., 1983). Overall, this asymmetric total synthesis was completed in only 8 steps from *N*-Boc-d-prolinol with a 33% overall yield (5 steps from known **6a** and a 35% overall yield).

With an established route to (−)-antofine, we pursued the total synthesis of (−)-tylocrebrine (**3b**), whose structure differs from that of (−)-antofine (**3a**) due to the presence of a methoxy group at C-5. Unlike the method for B-ring formation in **3a**, radical-mediated oxidative biaryl coupling could not be used for **3b** synthesis because of the regioselectivity problem. Therefore, we planned to employ palladium catalyzed C–H annulation (Ghosh et al., 2022; Thombal et al., 2022).

**SCHEME 4:**
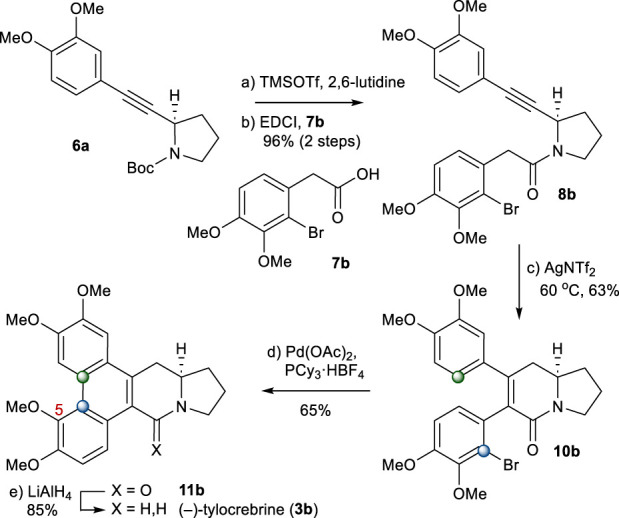
Synthesis of (−)-tylocrebrine (**3b**). Reagents and conditions: a) TMSOTf (2.0 equiv), 2,6-lutidine (3.0 equiv), CH_2_Cl_2_, 0°C, 10 min; b) **7b** (1.2 equiv), EDCI (1.1 equiv), DMAP (1.1 equiv), CH_2_Cl_2_, r.t., 20 h, 96% for 2 steps; c) AgNTf_2_ (0.1 equiv), TMSOTf (4.0 equiv), DIPEA (4.0 equiv), DCE, 60°C, 2 h, 63%; d) Pd(OAc)_2_ (0.2 equiv), PCy_3_^.^HBF_4_ (0.4 equiv), K_2_CO_3_ (4.0 equiv), 1,4-dioxane, 110°C, 1 h, 65%; e) LiAlH_4_ (2.0 equiv), THF, reflux, 1 h, 85%.

From intermediate **6a**, (−)-tylocrebrine (**3b**) was readily accessible. First, **6a** was coupled with 2-arylacetic acid **7b** to afford **8b**. The annulation of an amide with a tethered alkyne moiety in **8b** under the abovementioned conditions gave 5,6-dihydropyridinone **10b** directly in a 63% yield. After several trials, we found that the treatment of **10b** with Pd(OAc)_2_ and PCy_3_·HBF_4_ as the palladium source and ligand in dioxane, respectively, led to the formation of **11b** as the only detectable regioisomer (Campeau et al., 2006; Yadav et al., 2010). After the reduction of the amide group in **11b**, (−)-tylocrebrine (**3b**) was obtained in 5 steps from **6a** (Scheme 4).

**SCHEME 5:**
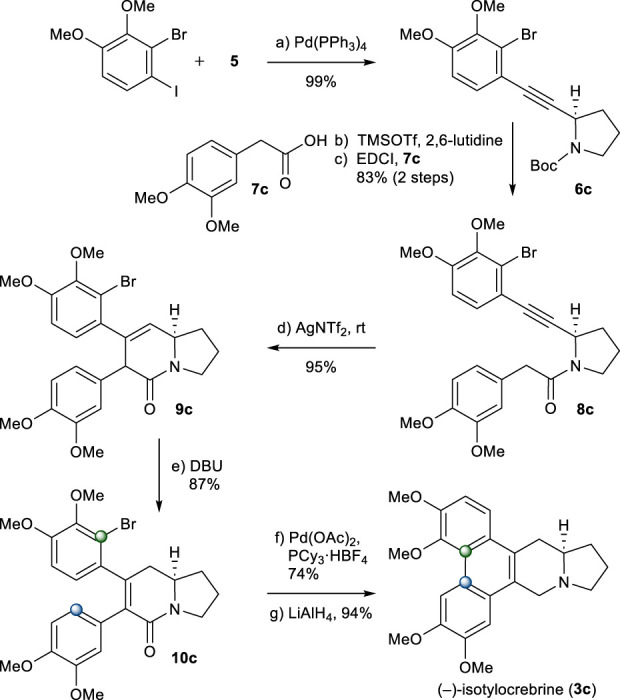
Synthesis of (−)-isotylocrebrine (**3c**). Reagents and conditions: a) Pd(PPh_3_)_4_ (0.05 equiv), CuI (0.1 equiv), *i*Pr_2_NH, r.t., 2 h, 99%; b) TMSOTf (2.0 equiv), 2,6-lutidine (3.0 equiv), CH_2_Cl_2_, 0°C, 10 min; c) **7c** (1.2 equiv), EDCI (1.1 equiv), DMAP (1.1 equiv), CH_2_Cl_2_, r.t., 12 h, 83% for 2 steps; d) AgNTf_2_ (0.1 equiv), TMSOTf (4.0 equiv), DIPEA (4.0 equiv), DCE, r.t., 1 h, 95%; e) DBU (4.0 equiv), toluene, 90°C, 1.5 h, 87%; f) Pd(OAc)_2_ (0.2 equiv), PCy_3_^.^HBF_4_ (0.4 equiv), K_2_CO_3_ (4.0 equiv), 1,4-dioxane, 110°C, 12 h, 74%; g) LiAlH_4_ (2.0 equiv), THF, reflux, 1 h, 94%.

The same chemistry was used for the total synthesis of (−)-isotylocrebrine (**3c**). Total synthesis of **3c** started from alkyne **5**. The Sonogashira coupling of **5** with 2-bromo-1-iodo-3,4-dimethoxybenzene chemoselectively afforded **6c** in an excellent yield (Weijiang et al., 2013). After *N*-Boc group removal, 2-aryl acetic acid **7c** was introduced to give amide **8c** in a good yield. Under the aforementioned one-pot cyclization/isomerization conditions, **8c** would not yield 5,6-dihydropyridinone **10c**. Instead, **9c** was formed in a high yield. Isomerization to **10c** was achieved when **9c** was treated with DBU in toluene at 90°C. Palladium-catalyzed B-ring formation, followed by the reduction of the amide group, provided (−)-isotylocrebrine (**3c**), as shown in Scheme 5.

**SCHEME 6:**
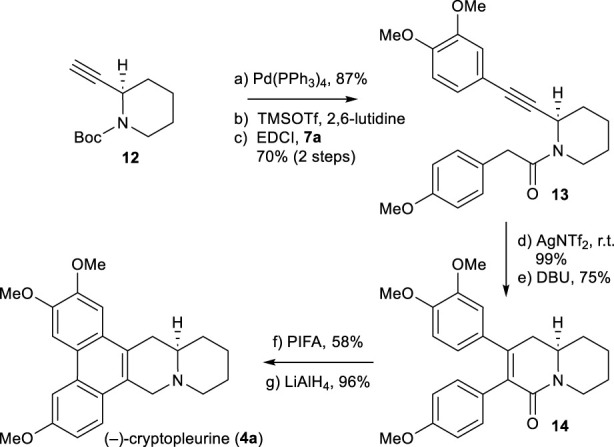
Synthesis of (−)-cryptopleurine (**4a**). Reagents and conditions: a) 3,4-dimethoxyiodobenzene, Pd(PPh_3_)_4_ (0.05 equiv), piperidine/MeCN (1:1), r.t., 14 h, 87%; b) TMSOTf (2.0 equiv), 2,6-lutidine (3.0 equiv), CH_2_Cl_2_, 0°C, 25 min; c) **7a** (1.2 equiv), EDCI (1.1 equiv), DMAP (1.1 equiv), CH_2_Cl_2_, r.t., 12 h, 70% for 2 steps; d) AgNTf_2_ (0.1 equiv), TMSOTf (4.0 equiv), DIPEA (4.0 equiv), DCE, r.t., 2 h, 99%; e) DBU (4.0 equiv), toluene, 90°C, 6 h, 75%; f) PIFA (1.1 equiv), BF_3_OEt_2_ (3.0 equiv), CH_2_Cl_2_, –10°C, 30 min, 58%; g) LiAlH_4_ (3.0 equiv), THF, reflux, 30 min, 96%.

Using the same chemistry described for the synthesis of (−)-antofine (**3a**), we accomplished the total synthesis of the phenanthroquinolizidine alkaloid (−)-cryptopleurine (**4a**), as shown in Scheme 6. A notable difference is the use of 2-alkyne-piperidine **12** in place of **5**. The total synthesis of **4a** was accomplished from **12** in 7 steps, using the process shown in Scheme 3. The spectra data and optical rotations of obtained alkaloids **3a**, **3b**, **3c**, and **4a** were in good agreement with those reported in the literature (Abe et al., 1995; Suzuki et al., 1995; Stærk et al., 2002; Niphakis et al., 2011).

## 3 Conclusion

In conclusion, we successfully developed a new synthetic strategy for the construction of functionalized dihydropyridinone rings through the annulation of an amide *α*-carbon with a tethered alkyne moiety. An unexplored reaction between amide and alkyne was realized through an *O*-silyl *N*,*O*-ketene acetal. Our method was applied for the total synthesis of phenanthroindolizidine and phenanthroquinolizidine alkaloids. Varying the coupling partners allowed for the culminative total synthesis of (−)-antofine (**3a**), (−)-tylocrebrine (**3b**), (−)-isotylocrebrine (**3c**), and (−)-cryptopleurine (**4a**). Further applications of this reaction for the synthesis of various functional dihydropyridinones and investigation of its extension to the total synthesis of other types of heterocyclic compounds are underway in our laboratory.

## Data Availability

The original contributions presented in the study are included in the article/Supplementary Material, further inquiries can be directed to the corresponding author.
