# Supplementary material for: Annulation of O-silyl N,O-ketene acetals with alkynes for the synthesis of dihydropyridinones and its application in concise total synthesis of phenanthroindolizidine alkaloids
Source: Front Chem. 2023 Sep 21;11:1267422. doi: 10.3389/fchem.2023.1267422 (PMC10551152; doi:10.3389/fchem.2023.1267422)

Supplementary Material

Annulation of *O*-Silyl *N*,*O*-Ketene Acetals with Alkynes for the Synthesis of Dihydropyridinones and its Application in Concise Total Synthesis of Phenanthroindolizidine Alkaloids

Seokwoo Lee[1,2†], Jae Eui Shin[1†], Ran Yoon[1], Hanbin Yoo[1], and Sanghee Kim[1*]

*** Correspondence:** Sanghee Kim, pennkim@snu.ac.kr

Table of Contents

1. General Information ………… 2

2. Experimental procedures and Spectroscopic data analysis ………… 3 – 35

3. Computational studies ………… 36 – 39

4. References ………… 40

5. Copies of NMR data ………… 41 – 66

1 General Information

All chemicals were of reagent grade and were used as purchased. All reactions were performed under an inert atmosphere of dry nitrogen using distilled dry solvents. The reactions were monitored by thin-layer chromatography (TLC) using silica gel 60 F-254 thin-layer plates. Compounds on the TLC plates were visualized under UV light and sprayed with either potassium permanganate or anisaldehyde solutions. Flash column chromatography was conducted on silica gel 60 (230–400 mesh). The melting points were measured using a Buchi B-540 melting point apparatus without correction. ^{1}H and ^{13}C NMR spectra were recorded on a JEOLJNM-ECZ400S/L1 (400 MHz) instrument at 298 K unless otherwise noted. Chemical shifts are reported in ppm (δ) units relative to the undeuterated solvent as a reference peak ($CDCl_3$–d_1: 7.26 ppm/^{1}H NMR, 77.16 ppm/^{13}C NMR). The following abbreviations are used to represent the NMR peak multiplicities: s (singlet), d (doublet), t (triplet), m (multiplet), dd (doublet of doublets), dt (doublet of triplets), dq (doublet of quartets), td (triplet of doublets), quin (quintuplet), and br (broad signal). Infrared (IR) spectra were measured using a JASCO FT/IR 4200 spectrometer. The optical rotations were measured on a Jasco P-2000 Polarimeter using sodium light (D line 589.3 nm) and a 3.5 × 100 mm or 3.5 × 10 mm cell. The values are reported as the specific optical rotation with exact temperature, concentration (c (10 mg/mL)) and solvent. High-resolution mass spectra (HRMS) were recorded using fast atom bombardment (FAB) mass spectrometry.

2 Experimental Procedures and Spectroscopic Data Analysis

2.1 Preparation of 1

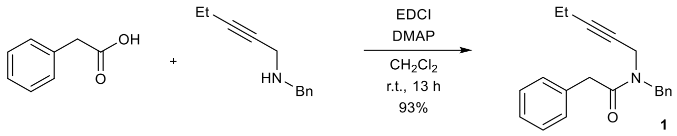

***N*-benzyl-*N*-(pent-2-yn-1-yl)-2-phenylacetamide (1):** To a solution of 2-phenylacetic acid (3.57 g, 26.2 mmol, 1.2 equiv) and *N*-benzylpent-2-yn-1-amine[1] (3.79 g, 21.8 mmol, 1.0 equiv) in dichloromethane (CH_2Cl_2, 73 mL), EDCI (4.61 g, 24.0 mmol, 1.1 equiv) and DMAP (2.94 g, 24.0 mmol, 1.1 equiv) were added at room temperature. The mixture was stirred at room temperature for 13 h and then quenched with saturated $NaHCO_3$ aqueous solution (50 mL) at room temperature. The mixture was extracted three times with CH_2Cl_2 (3 × 100 mL), and the combined organic fraction was dried over $MgSO_4$ and concentrated under reduced pressure. The residue was purified by flash chromatography on silica gel (hexane:EtOAc = 5:1, *v*/*v*) to obtain compound **1** (5.90 g, 93%) as a yellow oil.

TLC R_f = 0.2 (hexane:EtOAc = 5:1, *v*/*v*)

^{1}H NMR (400 MHz, $CDCl_3$) δ 7.39 – 7.22 (m, 9H), 7.14 (d, *J* = 6.9, 1.7 Hz, 1H), 4.70 (s, 1H), 4.65 (s, 1H), 4.22 (t, *J* = 2.3 Hz, 1H), 3.87 (s, 2H), 3.75 (s, 1H), 2.26 – 2.07 (m, 2H), 1.11 (dt, *J* = 11.4, 7.5 Hz, 3H)

^{13}C NMR (100 MHz, $CDCl_3$) δ (171.2, 171.1), 137.2, 136.6, (135.0, 134.9), (129.0, 128.9), 128.8 (2C), 128.6, 128.5, (127.7, 127.5), (127.0, 126.8), (86.6, 85.9), (74.1, 73.7), 50.4, 48.5, (41.1, 41.0), 37.2, 35.0, (13.9, 13.9), (12.5, 12.4)

IR (neat, cm^{-1}): 3065, 3033, 2978, 2939, 1654, 1454, 1417, 1360, 1173, 732, 697

HRMS (FAB): *m*/*z* calculated for $C_{20}H_{22}NO$ $[M+H]^+$ 292.1701, found 292.1705

2.2 Screening for the formation of 2

General procedure for the optimization of the reaction conditions (Table S1): Metal (0.010 mmol, 0.10 equiv) and base (0.40 mmol, 4.0 equiv) were added to a solution of compound **1** (29 mg, 0.10 mmol, 1.0 equiv) in solvent (2 mL, 0.05 M), as given in Table S1. The mixture was stirred under the conditions provided in Table S1 and quenched with saturated $NaHCO_3$ aqueous solution (5 mL) at 0 °C. The mixture was extracted three times with CH_2Cl_2 (3 × 10 mL), and the combined organic fraction was dried over $MgSO_4$ and concentrated under reduced pressure. The chemical yield was estimated by ^{1}H NMR analysis of the crude reaction mixture using tetrachloroethane ($C_2H_2Cl_4$) as the internal standard.

Table S1. Screening of metal and base for the formation of 2 from 1[a]

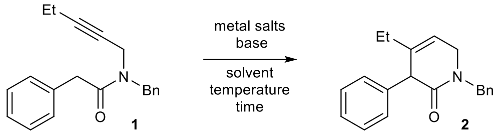

Entry	Metal salts	Base	Solvent	Temperature	Time	Yield (%)[b]
1	Ag_2CO_3	-	DCE	r.t.	6 h	0[c]
2	Ag_2CO_3	-	DCE	reflux	6 h	0[c]
3	$AgNTf_2$	-	DCE	r.t.	6 h	0[c]
4	$AgNTf_2$	-	DCE	reflux	6 h	0[c]
5	Ag_2CO_3	KO^{t}Bu	MeCN	r.t. to reflux	6 h	0[c]
6	Ag_2CO_3	K_2CO_3	MeCN	r.t. to reflux	6 h	0[c]
7	Ag_2CO_3	DBU	DCE	r.t. to reflux	6 h	0[c]
8	Ag_2CO_3	LiHMDS	CH_2Cl_2	–78 °C to reflux	6 h	0[c]
9	Ag_2CO_3	KHMDS	CH_2Cl_2	–78 °C to reflux	6 h	0[d]

[a] Reaction conditions: **1** (0.1 mmol), metal salts (0.1 equiv), base (4.0 equiv), solvent (0.05 M). [b] The chemical yield was estimated via ^{1}H NMR analysis of the crude reaction mixtures using tetrachloroethane ($C_2H_2Cl_4$) as the internal standard. [c] The starting material was remained. [d] The starting material was decomposed.

General procedure for the optimization of the reaction conditions (Table 1): Metal (0.010 mmol, 0.10 equiv) and base (0.40 mmol, 4.0 equiv) were added to a solution of compound **1** (29 mg, 0.10 mmol, 1.0 eq.) in DCE (2 mL, 0.05 M), as given in Table 1. The mixture was stirred under the conditions given in Table 1 and quenched with saturated $NaHCO_3$ aqueous solution (5 mL) at 0 °C. The mixture was extracted three times with CH_2Cl_2 (3 × 10 mL), and the combined organic fraction was dried over $MgSO_4$ and concentrated under reduced pressure. The chemical yield was estimated by ^{1}H NMR analysis of the crude reaction mixture using tetrachloroethane ($C_2H_2Cl_4$) as the internal standard. The crude mixture was purified by flash chromatography on silica gel (hexane:EtOAc = 5:1, *v*/*v*) to obtain compound **2** as a colorless oil.

1-benzyl-4-ethyl-3-phenyl-3,6-dihydropyridin-2(1*H*)-one (2)

TLC R_f = 0.15 (hexane:EtOAc = 5:1, *v*/*v*)

^{1}H NMR (400 MHz, $CDCl_3$) δ 7.37 – 7.25 (m, 8H), 7.19 (dd, *J* = 7.7, 1.8 Hz, 2H), 5.62 (dt, *J* = 3.9, 2.0 Hz, 1H), 4.63 (s, 2H), 4.10 (t, *J* = 3.2 Hz, 1H), 4.05 – 3.96 (m, 1H), 3.88 – 3.74 (m, 1H), 2.02 – 1.83 (m, 2H), 0.99 (t, *J* = 7.4 Hz, 3H)

^{13}C NMR (100 MHz, $CDCl_3$) δ 169.2, 139.7, 139.5, 137.0, 128.8 (2C), 128.7 (2C), 128.3 (2C), 128.2 (2C), 127.6, 127.4, 114.5, 52.1, 49.9, 48.0, 26.8, 11.5

IR (neat, cm^{-1}): 3064, 3030, 2967, 2878, 1643, 1487, 1453, 1261, 751, 700

HRMS (FAB): *m*/*z* calculated for $C_{20}H_{22}NO$ $[M+H]^+$ 292.1701, found 292.1706

Scale-up reaction for the synthesis of compound 2: To a solution of **1** (291 mg, 1.0 mmol, 1.0 equiv) in DCE (20 mL, 0.05 M), $AgNTf_2$ (39 mg, 0.10 mmol, 0.1 equiv), TMSOTf (0.73 mL, 4.0 mmol, 4.0 equiv) and DIPEA (0.70 mL, 4.0 mmol, 4.0 equiv) were added at room temperature. The mixture was stirred at room temperature for 3 h and quenched with saturated $NaHCO_3$ aqueous solution (40 mL) at 0 °C. The mixture was extracted three times with CH_2Cl_2 (3 × 40 mL), and the combined organic fraction was dried over $MgSO_4$ and concentrated under reduced pressure. The crude mixture was purified by flash chromatography on silica gel (hexane:EtOAc = 5:1, *v*/*v*) to obtain compound **2** (262 mg, 90%) as a colorless oil.

General procedure for the optimization of the reaction conditions (Table S2): $AgNTf_2$ (0.010 mmol, 0.10 equiv), Silyl reagent (0.40 mmol, 4.0 equiv) and DIPEA (0.40 mmol, 4.0 equiv) were added to a solution of compound **1** (29 mg, 0.10 mmol, 1.0 equiv) in DCE (2 mL, 0.05 M), as given in Table S2. The mixture was stirred under the conditions provided in Table S2 and quenched with saturated $NaHCO_3$ aqueous solution (5 mL) at 0 °C. The mixture was extracted three times with CH_2Cl_2 (3 × 10 mL), and then the combined organic fraction was dried over $MgSO_4$ and concentrated under reduced pressure. The chemical yield was estimated by ^1H NMR analysis of the crude reaction mixture using tetrachloroethane ($C_2H_2Cl_4$) as the internal standard.

Table S2. Screening of silyl reagent for the formation of 2 from 1[a]

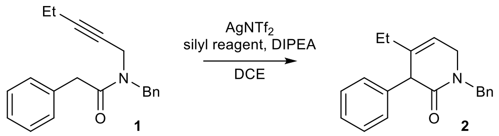

Entry	Silyl reagent	Time	Temperature	Yield of **1** (%)[b]	Yield of **2** (%)[b]
1	TMSOTf	3 h	r.t.	0	96 (93)[c]
2	TBSOTf	6 h	r.t.	21	63
3	TBSOTf	6 h	reflux	15	42
4	TIPSOTf	6 h	r.t.	90	2
5	TIPSOTf	6 h	reflux	88	5
6	TMSCl	6 h	r.t.	99	0
7	TMSCl	6 h	reflux	95	0
8	TMSI	6 h	r.t.	71	0
9	TMSI	6 h	reflux	63	0

[a] Reaction conditions: **1** (0.1 mmol), $AgNTf_2$ (0.1 equiv), silyl reagent (4.0 equiv), DIPEA (4.0 equiv), DCE (0.05 M). [b] The chemical yield was estimated via ^1H NMR analysis of the crude reaction mixtures using tetrachloroethane ($C_2H_2Cl_4$) as the internal standard. [c] Isolated yield.

General procedure for the optimization of the reaction conditions (Table S3): AgNTf$_2$ (0.010 mmol, 0.10 equiv), TMSOTf (0.40 mmol, 4.0 equiv) and DIPEA (0.40 mmol, 4.0 equiv) were added to a solution of compound **1** (29 mg, 0.10 mmol, 1.0 equiv) in solvent (2 mL, 0.05 M), as given in Table S3. The mixture was stirred at room temperature under the conditions provided in Table S3 and quenched with saturated $NaHCO_3$ aqueous solution (5 mL) at 0 °C. The mixture was extracted three times with CH_2Cl_2 (3 × 10 mL), and then the combined organic fraction was dried over $MgSO_4$ and concentrated under reduced pressure. The chemical yield was estimated by ^{1}H NMR analysis of the crude reaction mixture using tetrachloroethane ($C_2H_2Cl_4$) as the internal standard.

Table S3. Screening of solvent for the formation of 2 from 1[a]

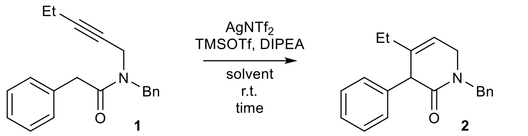

Entry	Solvent	Time	Yield (%)[b]
1	DCE	3 h	96 (93)[c]
2	CH_2Cl_2	4 h	95 (92)[c]
3	MeOH	3 h	0[d]
4	MeCN	3 h	0[d]
5	THF	3 h	0[d]
6	Toluene	12 h	24 (brsm 84)
7	DMF	3 h	0[d]

[a] Reaction conditions: **1** (0.1 mmol), metal (0.1 equiv), TMSOTf (4.0 equiv), DIPEA (4.0 equiv), solvent (0.05 M). [b] The chemical yield was estimated via ^{1}H NMR analysis of the crude reaction mixtures using tetrachloroethane ($C_2H_2Cl_4$) as the internal standard. [c] Isolated yield. [d] The starting material was remained.

2.3 Total synthesis of (–)-antofine (3a)

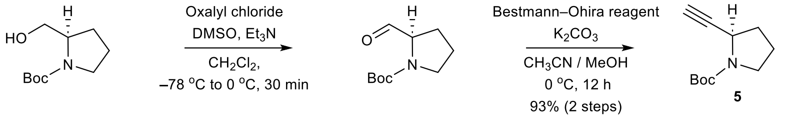

***tert*-butyl (*R*)-2-ethynylpyrrolidine-1-carboxylate (5):** Oxalyl chloride (2.0 M in CH_2Cl_2, 1.5 equiv, 37.5 mmol, 18.8 mL) was added to a solution of DMSO (3.0 equiv, 75.0 mmol, 5.33 mL) in dichloromethane (100 mL) at –78 °C, and the mixture was stirred at the same temperature for 30 min. A solution of *N*-Boc-D-prolinol (5.03 g, 25.0 mmol, 1.0 equiv) in dichloromethane (25 mL) was added to the reaction mixture at –78 °C, and the mixture was stirred at the same temperature for 30 min. Then, triethylamine (4.0 equiv, 100 mmol, 13.9 mL) was added to the reaction mixture at –78 °C, which was allowed to slowly warm to 0 °C and stirred for 30 min. The reaction was quenched with saturated aqueous $NaHCO_3$ solution, poured into water, extracted twice with CH_2Cl_2, dried over $MgSO_4$ and concentrated *in vacuo*. The residue was filtered through a silica gel pad and rinsed with EtOAc. The crude mixture was used in the next step without further purification. To a solution of Bestmann–Ohira reagent (1.45 equiv, 36.3 mmol) in acetonitrile (125 mL), K_2CO_3 (4.0 equiv, 100 mmol, 13.8 g) was added at 0 °C and stirred for 2 h. Then, a solution of the crude mixture in methanol (125 mL) was added dropwise to the reaction mixture and stirred at room temperature for 12 h. The reaction mixture was filtered through a Celite pad and rinsed with EtOAc. The filtrate was then concentrated *in vacuo.* EtOAc and brine were added to the residue, which was then extracted twice with EtOAc, dried over $MgSO_4$ and concentrated *in vacuo*. The crude mixture was purified by flash chromatography on silica gel (hexane:EtOAc = 5:1, *v*/*v*) to obtain compound **5** (4.54 g, 93%, 2 steps) as a yellow oil. The product matched reported spectra.[2]

TLC R_f = 0.4 (hexane:EtOAc = 5:1, *v*/*v*)

^{1}H NMR (400 MHz, $CDCl_3$) δ 4.60 – 4.24 (m, 1H), 3.50 – 3.38 (m, 1H), 3.36 – 3.20 (m, 1H), 2.26 – 2.13 (m, 1H), 2.14 – 1.95 (m, 3H), 1.90 – 1.79 (m, 1H), 1.47 (s, 9H)

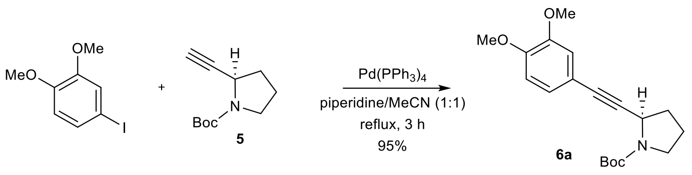

***tert*-butyl (*R*)-2-((3,4-dimethoxyphenyl)ethynyl)pyrrolidine-1-carboxylate (6a):** To a solution of 4-iodo-1,2-dimethoxybenzene (475 mg, 1.80 mmol, 1.1 equiv) and **5** (320 mg, 1.64 mmol, 1.0 equiv) in piperidine (1.7 mL) and CH_3CN (1.7 mL), $Pd(PPh_3)_4$ (94 mg, 0.082 mmol, 0.05 equiv) was added at room temperature. The mixture was refluxed for 3 h and quenched with saturated NH_4Cl aqueous solution (20 mL) at room temperature. The mixture was extracted three times with EtOAc (3 × 20 mL), and the combined organic fraction was dried over $MgSO_4$ and concentrated under reduced pressure. The residue was purified by flash chromatography on silica gel (hexane/EtOAc = 5:1, *v*/*v*) to obtain compound **6a** (517 mg, 95%) as a yellow oil.

TLC R_f = 0.2 (hexane:EtOAc = 5:1, *v*/*v*)

^{1}H NMR (400 MHz, $CDCl_3$) δ 6.99 (d, *J* = 8.2 Hz, 1H), 6.89 (s, 1H), 6.77 (d, *J* = 8.3 Hz, 1H), 4.89 – 4.39 (m, 1H), 3.87 (s, 3H), 3.86 (s, 3H), 3.60 – 3.48 (m, 1H), 3.42 – 3.29 (m, 1H), 2.12 – 2.08 (m, 3H), 1.93 – 1.88 (m, 1H), 1.49 (s, 9H)

^{13}C NMR (100 MHz, $CDCl_3$) δ 154.3, 149.4, 148.6, 124.9, 115.6, 114.5, 111.1, 88.5, 81.6, 79.7, 56.0 (2C), 48.9, 45.8, 33.9, 28.7 (3C), (24.62, 23.94)

IR (neat, cm^{-1}): 2976, 2935, 2879, 1738, 1625, 1405, 1362, 1217, 1149, 868

Optical Rotation: $[\alpha]_D^{25}$ +134.6 (*c* 0.5, $CHCl_3$)

HRMS (FAB): *m*/*z* calculated for $C_{15}H_{18}NO_4$ $[M–C_4H_9]^+$ 276.1236, found 276.1235

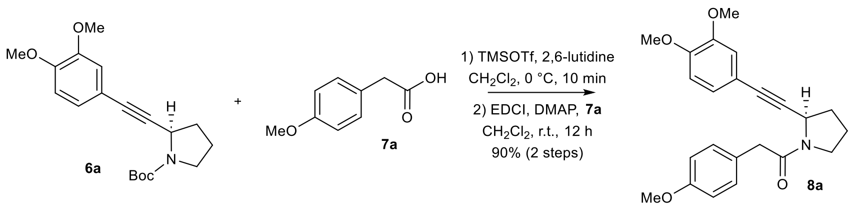

(*R*)-1-(2-((3,4-dimethoxyphenyl)ethynyl)pyrrolidin-1-yl)-2-(4-methoxyphenyl)ethan-1-one (8a): To a solution of **6a** (3.95 g, 11.9 mmol, 1.0 equiv) in CH_2Cl_2 (40 mL), TMSOTf (4.30 mL, 23.9 mmol, 2.0 equiv) and 2,6-lutidine (4.20 mL, 35.8 mmol, 3.0 equiv) were added at 0 °C. The mixture was stirred at 0 °C for 10 min and quenched with saturated Na_2CO_3 aqueous solution (100 mL) at 0 °C. The mixture was extracted three times with CH_2Cl_2 (3 × 150 mL), and the combined organic fraction was dried over $MgSO_4$ and concentrated under reduced pressure. The crude mixture was used in the next step without further purification. **7a** (1.59 g, 9.54 mmol, 1.2 equiv), EDCI (1.68 g, 8.75 mmol, 1.1 equiv) and DMAP (1.07 g, 8.75 mmol, 1.1 equiv) were added to a solution of the crude mixture in CH_2Cl_2 (27 mL) at 0 °C. The reaction mixture was stirred for 12 h at room temperature, quenched with saturated aqueous $NaHCO_3$ solution (100 mL), poured into water, extracted with twice CH_2Cl_2 (2 × 150 mL), dried over $MgSO_4$ and then concentrated *in vacuo*. The crude mixture was purified by flash chromatography on silica gel (hexane:EtOAc = 3:1, *v*/*v*) to obtain compound **8a** (2.82 g, 90%, 2 steps) as a yellow oil.

TLC R_f = 0.2 (hexane:EtOAc = 3:1, *v*/*v*)

^{1}H NMR (400 MHz, $CDCl_3$) δ 7.26 – 7.18 (m, 2H), 6.98 (dt, *J* = 8.2, 1.6 Hz, 1H), 6.90 – 6.74 (m, 4H), 5.02 – 4.97 (m, 0.4H), 4.69 (dd, *J* = 6.4, 2.6 Hz, 0.6H), 3.87 – 3.83 (m, 7H), 3.76 (d, *J* = 2.6 Hz, 3H), 3.72 – 3.63 (m, 1H), 3.62 – 3.55 (m, 1H), 3.52 – 3.35 (m, 1H), 2.21 – 1.86 (m, 4H)

^{13}C NMR (400 MHz, $CDCl_3$) δ (170.5, 169.6), 158.5, (149.7, 149.3), (148.7, 148.5), 130.2, 130.0, (127.1, 126.7), (125.1, 125.0), 115.4, (114.6, 114.5), 114.3, 114.0, (111.0, 110.8), (87.4, 86.8), (83.8, 81.8), 56.0, 55.9, 55.3, (49.2, 48.4), (46.6, 46.1), (41.3, 40.9), (34.6, 32.6), (25.0, 23.1)

IR (neat, cm^{-1}): 2954, 2926, 2873, 2837, 1647, 1512, 1407, 1244, 1137, 1024, 812

Optical Rotation: $[\alpha]_D^{25}$ +135.6 (*c* 0.5, $CHCl_3$)

HRMS (FAB): *m*/*z* calculated for $C_{23}H_{26}NO_4$ $[M+H]^+$ 380.1862, found 380.1867

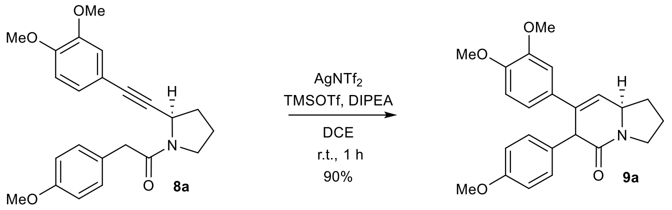

(8a*R*)-7-(3,4-dimethoxyphenyl)-6-(4-methoxyphenyl)-2,3,6,8a-tetrahydroindolizin-5(1*H*)-one (9a): To a solution of **8a** (71 mg, 0.19 mmol, 1.0 equiv) in 1,2-dichloroethane (3.8 mL), $AgNTf_2$ (7.3 mg, 0.019 mmol, 0.1 equiv), TMSOTf (0.14 mL, 0.75 mmol, 4.0 equiv) and DIPEA (0.13 mL, 0.75 mmol, 4.0 equiv) were added at room temperature. The mixture was stirred at room temperature for 1 h and quenched with saturated $NaHCO_3$ aqueous solution (10 mL) at 0 °C. The mixture was extracted three times with CH_2Cl_2 (3 × 20 mL), and the combined organic fraction was dried over $MgSO_4$ and concentrated under reduced pressure. The crude mixture was purified by flash chromatography on silica gel (CH_2Cl_2:EtOAc = 3:1, *v*/*v*) to obtain compound **9a** (64 mg, 90%) as a yellow oil.

TLC R_f = 0.4 (CH_2Cl_2:EtOAc = 2:1, *v*/*v*)

^{1}H NMR (400 MHz, $CDCl_3$) δ 7.10 (d, *J* = 8.6 Hz, 2H), 6.79 (dd, *J* = 8.3, 2.1 Hz, 1H), 6.74 – 6.63 (m, 4H), 6.24 (dd, *J* = 3.0, 1.6 Hz, 1H), 4.53 (dd, *J* = 5.3, 1.5 Hz, 1H), 4.27 – 4.15 (m, 1H), 3.86 – 3.81 (m, 1H), 3.79 (s, 3H), 3.75 (s, 3H), 3.70 (s, 3H), 3.36 (ddd, *J* = 12.9, 10.1, 3.7 Hz, 1H), 2.36 – 2.23 (m, 1H), 2.13 – 2.01 (m, 1H), 2.00 – 1.88 (m, 1H), 1.75 – 1.53 (m, 1H)

^{13}C NMR (100 MHz, $CDCl_3$) δ 167.7, 158.5, 148.5, 148.4, 136.9, 132.1, 131.9, 129.9 (2C), 120.4, 119.0, 114.0 (2C), 110.8, 109.8, 59.4, 55.9 (2C), 55.2, 49.7, 43.9, 33.1, 22.6

IR (neat, cm^{-1}): 2960, 2937, 2910, 2838, 1642, 1512, 1455, 1249, 1027, 842, 761

Optical Rotation: $[\alpha]_D^{25}$ +54.8 (*c* 0.5, $CHCl_3$)

HRMS (FAB): *m*/*z* calculated for $C_{23}H_{26}NO_4$ $[M+H]^+$ 380.1862, found 380.1867

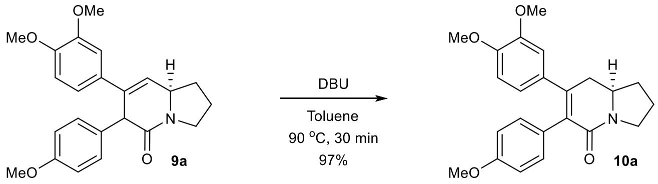

(*R*)-7-(3,4-dimethoxyphenyl)-6-(4-methoxyphenyl)-2,3,8,8a-tetrahydroindolizin-5(1*H*)-one (10a): To a solution of **9a** (30 mg, 0.079 mmol, 1.0 equiv) in toluene (0.8 mL), DBU (0.05 mL, 0.32 mmol, 4.0 equiv) was added at room temperature. The mixture was stirred at 90 °C for 30 min. After the completion of the reaction, as monitored by TLC, the reaction mixture was concentrated under reduced pressure. The crude mixture was purified by flash chromatography on silica gel (CH_2Cl_2:EtOAc, 4:1, *v*/*v*) to obtain compound **10a** (29 mg, 97%) as a yellow oil.

TLC R_f = 0.5 (CH_2Cl_2:EtOAc, 2:1, *v*/*v*)

^{1}H NMR (400 MHz, $CDCl_3$) δ 6.97 (dd, *J* = 9.2, 2.5 Hz, 2H), 6.71 (td, *J* = 6.1, 5.0, 2.9 Hz, 4H), 6.39 (d, *J* = 1.6 Hz, 1H), 3.98 – 3.85 (m, 1H), 3.80 (s, 3H), 3.72 (s, 3H), 3.68 – 3.59 (m, 1H), 3.54 – 3.48 (m, 1H), 3.46 (s, 3H), 2.89 – 2.66 (m, 2H), 2.34 – 2.24 (m, 1H), 2.14 – 2.02 (m, 1H), 1.95 – 1.78 (m, 1H), 1.77 – 1.58 (m, 1H)

^{13}C NMR (100 MHz, $CDCl_3$) δ 164.7, 158.6, 148.5, 147.9, 145.6, 132.3 (3C), 130.8, 128.4, 120.9, 113.5 (2C), 112.6, 110.5, 55.8 (2C), 55.5, 55.3, 45.3, 37.0, 33.7, 23.2

IR (neat, cm^{-1}): 2957, 2879, 2838, 1640, 1510, 1435, 1243, 1171, 1026, 758

Optical Rotation: $[\alpha]_D^{25}$ –34.9 (*c* 0.5, $CHCl_3$)

HRMS (FAB): *m*/*z* calculated for $C_{23}H_{26}NO_4$ $[M+H]^+$ 380.1862, found 380.1868

One-step synthesis of 10a from 8a

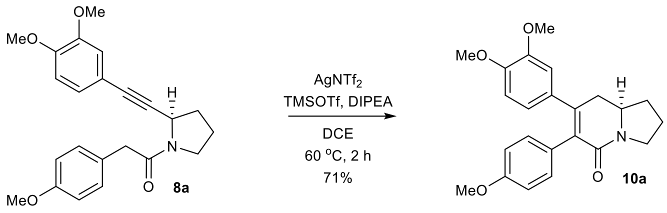

(*R*)-7-(3,4-dimethoxyphenyl)-6-(4-methoxyphenyl)-2,3,8,8a-tetrahydroindolizin-5(1*H*)-one (10a): To a solution of **8a** (89 mg, 0.23 mmol, 1.0 equiv) in 1,2-dichloroethane (4.7 mL), $AgNTf_2$ (9.1 mg, 0.023 mmol, 0.1 equiv), TMSOTf (0.17 mL, 0.94 mmol, 4.0 equiv) and DIPEA (0.16 mL, 0.94 mmol, 4.0 equiv) were added at room temperature. The mixture was stirred at 60 °C for 2 h and quenched with saturated $NaHCO_3$ aqueous solution (10 mL) at 0 °C. The mixture was extracted three times with CH_2Cl_2 (3 × 20 mL), and the combined organic fraction was dried over $MgSO_4$ and concentrated under reduced pressure. The crude mixture was purified by flash chromatography on silica gel (CH_2Cl_2:EtOAc = 4:1, *v*/*v*) to obtain compound **10a** (63 mg, 71%) as a yellow oil.

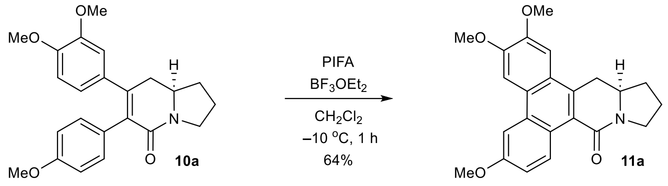

(*R*)-2,3,6-trimethoxy-12,13,13a,14-tetrahydrodibenzo[*f*,*h*]pyrrolo[1,2-*b*]isoquinolin-9(11*H*)-one (11a): To a solution of **10a** (286 mg, 0.754 mmol, 1.0 equiv) in CH_2Cl_2 (4.0 mL), (bis(trifluoroacetoxy)iodo)benzene (PIFA, 357 mg, 0.830 mmol, 1.1 equiv) and BF_3OEt_2 (0.14 mL, 1.1 mmol, 1.5 equiv) were added at –10 °C. The mixture was stirred at –10 °C for 1 h and quenched with saturated $NaHCO_3$ aqueous solution (10 mL) at –10 °C. The mixture was extracted three times with CH_2Cl_2 (3 × 20 mL), and the combined organic fraction was dried over $MgSO_4$ and concentrated under reduced pressure. The crude mixture was purified by flash chromatography on silica gel (CH_2Cl_2:EtOAc = 5:1, *v*/*v*) to obtain compound **11a** (183 mg, 64%) as a white solid.

TLC R_f = 0.2 (CH_2Cl_2:EtOAc = 5:1, *v*/*v*)

Melting point: 220–225 °C

^{1}H NMR (400 MHz, $CDCl_3$) δ 9.26 (d, *J* = 9.3 Hz, 1H), 7.81 (s, 1H), 7.78 (d, *J* = 2.6 Hz, 1H), 7.23 (dd, *J* = 9.3, 2.5 Hz, 1H), 7.20 (s, 1H), 4.09 (s, 3H), 4.00 (s, 3H), 3.99 (s, 3H), 3.87 – 3.72 (m, 4H), 3.44 (dd, *J* = 15.7, 4.0 Hz, 1H), 2.81 (dd, *J* = 15.2, 1.7 Hz, 1H), 2.51 – 2.30 (m, 1H), 2.17 – 2.09 (m, 1H), 2.02 – 1.78 (m, 1H)

^{13}C NMR (100 MHz, $CDCl_3$) δ 164.4, 157.7, 150.1, 149.5, 132.7, 131.0, 129.7 (2C), 126.4, 124.4, 123.6, 115.3, 104.9, 104.1, 103.7, 77.5, 77.2, 76.8, 56.1, 56.0, 55.6, 55.3, 45.4, 33.9, 32.6, 23.7

IR (neat, cm^{-1}): 2958, 2926, 2872, 2837, 1618, 1509, 1439, 1260, 1203, 1039, 838, 748

Optical Rotation: $[\alpha]_D^{25}$ –354.3 (*c* 0.5, $CHCl_3$)

HRMS (FAB): *m*/*z* calculated for $C_{23}H_{24}NO_4$ $[M+H]^+$ 378.1705, found 378.1708

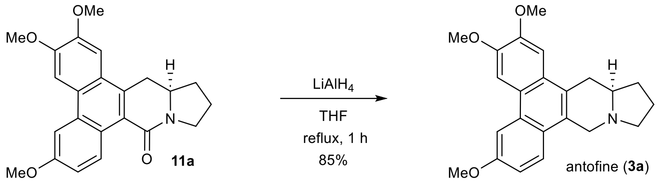

(–)-antofine (3a): To a solution of **11a** (52 mg, 0.14 mmol, 1.0 equiv) in THF (7 mL), $LiAlH_4$ (1.0 M in THF, 2.0 equiv, 0.28 mmol, 0.28 mL) was added at 0 °C. The mixture was refluxed for 1 h. The mixture was cooled to 0 °C and quenched with water (0.10 mL), 15% NaOH aqueous solution (0.10 mL) and water (0.30 mL) slowly. The mixture was allowed to warm to room temperature and stirred for 15 min. The suspension was added $MgSO_4$ and stirred for 15 min, filtered through a Celite pad and concentrated under reduced pressure. The crude mixture was purified by flash chromatography on silica gel (CH_2Cl_2:MeOH = 20:1, *v*/*v*) to obtain (–)-antofine (**3a**, 43 mg, 85%) as a white solid.

TLC R_f = 0.2 (CH_2Cl_2:MeOH = 20:1, *v*/*v*)

Melting point: 205–214 °C

^{1}H NMR (400 MHz, $CDCl_3$) δ 7.85 (s, 1H), 7.84 (d, J = 2.8 Hz, 1H), 7.74 (d, J = 9.0 Hz, 1H), 7.23 (s, 1H), 7.16 (dd, J = 9.0, 2.5 Hz, 1H), 4.65 (dd, J = 15.1, 1.3 Hz, 1H), 4.08 (s, 3H), 4.03 (s, 3H), 3.98 (s, 3H), 3.64 (dt, J = 15.0, 2.2 Hz, 1H), 3.45 (td, J = 8.7, 2.4 Hz, 1H), 3.25 (ddd, J = 15.9, 4.1, 1.7 Hz, 1H), 2.94 – 2.80 (m, 1H), 2.51 – 2.37 (m, 2H), 2.27 – 2.12 (m, 1H), 2.11 – 1.94 (m, 1H), 1.97 – 1.81 (m, 1H), 1.83 – 1.67 (m, 1H)

^{13}C NMR (100 MHz, $CDCl_3$) δ 157.5, 149.4, 148.4, 130.2, 127.0, 126.3, 125.5, 124.3, 124.1, 123.6, 114.9, 104.7, 104.0, 103.8, 60.3, 56.1, 55.9, 55.6, 55.0, 53.7, 33.4, 31.2, 21.6

IR (neat, cm^{-1}): 3002, 2938, 2834, 1618, 1513, 1467, 1258, 1203, 1034, 749

Optical Rotation: $[\alpha]_D^{25}$ –124.0 (*c* 0.15, $CHCl_3$)

HRMS (FAB): *m*/*z* calculated for $C_{23}H_{26}NO_3$ $[M+H]^+$ 364.1913, found 364.1912

2.4 Total synthesis of (–)-tylocrebrine (3b)

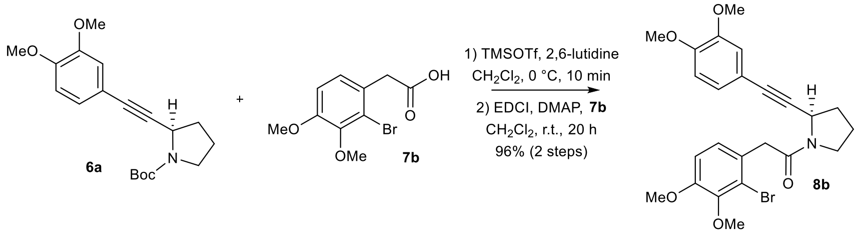

(*R*)-2-(2-bromo-3,4-dimethoxyphenyl)-1-(2-((3,4-dimethoxyphenyl)ethynyl)pyrrolidin-1-yl)ethan-1-one (8b)

Note: The procedure used to prepare **8a** with **6a** (637 mg, 1.92 mmol, 1.0 equiv) and 2-(2-bromo-3,4-dimethoxyphenyl)acetic acid[3] (**7b**, 635 mg, 2.31 mmol, 1.2 equiv) was followed. The crude mixture was purified by flash chromatography on silica gel (hexane:EtOAc = 1:1, *v*/*v*) to obtain **8b** (832 mg, 96%, 2 steps) as a white wax.

TLC R_f = 0.1 (hexane:EtOAc = 1:1, *v*/*v*)

^{1}H NMR (400 MHz, $CDCl_3$) δ 7.06 (dd, *J* = 15.3, 8.5 Hz, 1H), 7.05 – 6.95 (m, 1H), 6.91 (dd, *J* = 13.5, 1.9 Hz, 1H), 6.86 – 6.69 (m, 2H), 5.05 (dd, *J* = 6.9, 2.5 Hz, 0.4H), 4.83 (t, *J* = 4.9 Hz, 0.6H), 3.99 (s, 1H), 3.89 – 3.85 (m, 6H), 3.85 – 3.82 (m, 6H), 3.77 – 3.63 (m, 2H), 3.60 – 3.44 (m, 1H), 2.42 – 2.18 (m, 2H), 2.18 – 1.90 (m, 2H)

^{13}C NMR (100 MHz, $CDCl_3$) δ (169.4, 168.7), 152.5, (149.7, 149.4), (148.7, 148.6), 146.6, (128.5, 128.1), (126.2, 125.7), (125.2, 125.1), (120.8, 120.7), (115.5, 114.73), (114.68, 114.4), (111.5, 111.4), (111.1, 110.9), (87.4, 86.7), (84.0, 81.9), 60.5, 56.2, (56.1, 56.03), 55.98, (49.5, 48.6), (46.6, 46.2), (41.69, 41.65), (34.7, 32.7), (25.1, 23.3)

IR (neat, cm^{-1}): 2956, 2932, 2875, 2840, 1651, 1514, 1488, 1403, 1266, 1031, 813

Optical Rotation: $[\alpha]_D^{25}$ +82.3 (*c* 0.5, $CHCl_3$)

HRMS (FAB): *m*/*z* calculated for $C_{24}H_{27}BrNO_5$ $[M+H]^+$ 488.1073, found 488.1077

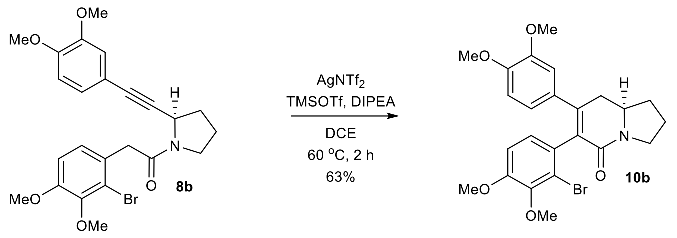

(*R*)-6-(2-bromo-3,4-dimethoxyphenyl)-7-(3,4-dimethoxyphenyl)-2,3,8,8a-tetrahydroindolizin-5(1*H*)-one (10b)

Note: The procedure of one-step synthesis used to prepare **10a** with **8b** (24 mg, 0.049 mmol, 1.0 equiv) was followed. The crude mixture was purified by flash chromatography on silica gel (CH_2Cl_2:EtOAc = 2:1, *v*/*v*) to obtain **10b** (15 mg, 63%) as a yellow wax.

TLC R_f = 0.2 (CH_2Cl_2:EtOAc = 2:1, *v*/*v*)

^{1}H NMR (400 MHz, $CDCl_3$) δ 6.87 – 6.75 (m, 1H), 6.75 – 6.66 (m, 2H), 6.63 – 6.54 (m, 1H), 6.43 (s, 1H), 4.12 – 3.98 (m, 1H), 3.93 – 3.77 (m, 9H), 3.75 – 3.62 (m, 2H), 3.53 (s, 3H), 3.00 – 2.86 (m, 1H), 2.79 (dd, *J* = 16.0, 4.8 Hz, 1H), 2.37 – 2.26 (m, 1H), 2.18 – 2.06 (m, 1H), 1.98 – 1.84 (m, 1H), 1.82 – 1.69 (m, 1H)

^{13}C NMR (100 MHz, $CDCl_3$) δ 163.8, 152.8, 148.8, 148.1, 146.5, 132.0, 131.4, 131.1, 127.8, 120.7, 120.6, 120.0, 111.7, 111.5, 110.6, 60.7, 56.2, 55.9 (2C), 55.6, 45.4, 37.0, 33.6, 23.4

IR (neat, cm^{-1}): 2957, 2928, 2840, 1645, 1515, 1440, 1264, 1190, 1028, 810, 762

Optical Rotation: $[\alpha]_D^{25}$ –115.5 (*c* 0.5, $CHCl_3$)

HRMS (FAB): *m*/*z* calculated for $C_{24}H_{27}BrNO_5$ $[M+H]^+$ 488.1073, found 488.1081

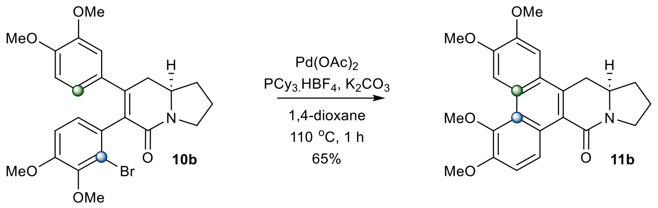

(*R*)-2,3,5,6-tetramethoxy-12,13,13a,14-tetrahydrodibenzo[*f*,*h*]pyrrolo[1,2-*b*]isoquinolin-9(11*H*)-one (11b)

To a solution of **10b** (48 mg, 0.10 mmol, 1.0 equiv) in 1,4-dioxane (2 mL), $Pd(OAc)_2$ (4.5 mg, 0.020 mmol, 0.2 equiv), $PCy_3{\cdot}HBF_4$ (15 mg, 0.041 mmol, 0.4 equiv) and K_2CO_3 (54 mg, 0.39 mmol, 4.0 equiv) were added at room temperature. The mixture was stirred at 110 °C for 1 h. The mixture was filtered through a Celite pad. The filtrate was concentrated under reduced pressure. The crude mixture was purified by flash chromatography on silica gel (CH_2Cl_2:EtOAc = 4:1, *v*/*v*) to obtain compound **11b** (26 mg, 65%) as a white solid.

TLC R_f = 0.8 (CH_2Cl_2:EtOAc = 1:1, *v*/*v*)

Melting point: 191–198 °C

^{1}H NMR (400 MHz, $CDCl_3$) δ 9.33 (s, 1H), 9.06 (d, *J* = 9.3 Hz, 1H), 7.33 – 7.28 (m, 2H), 4.07 (s, 3H), 4.04 (s, 3H), 4.02 (s, 3H), 3.93 – 3.88 (m, 1H), 3.86 (s, 3H), 3.83 – 3.70 (m, 2H), 3.49 (dd, *J* = 15.8, 4.1 Hz, 1H), 2.85 (dd, *J* = 15.7, 13.2 Hz, 1H), 2.52 – 2.35 (m, 1H), 2.21 – 2.09 (m, 1H), 2.00 – 1.77 (m, 2H)

^{13}C NMR (100 MHz, $CDCl_3$) δ 164.4, 150.8, 149.5, 148.9, 145.3, 133.4, 126.0, 125.3, 124.8, 124.4 (2C), 123.9, 112.6, 109.26, 104.4, 60.2, 56.4, 55.9, 55.9, 55.2, 45.5, 33.9, 33.0, 23.7

IR (neat, cm^{-1}): 2937, 2878, 2842, 1632, 1510, 1434, 1258, 1113, 1041, 752

Optical Rotation: $[\alpha]_D^{25}$ –323.5 (*c* 0.5, $CHCl_3$)

HRMS (FAB): *m*/*z* calculated for $C_{24}H_{26}NO_5$ $[M+H]^+$ 408.1811, found 408.1809

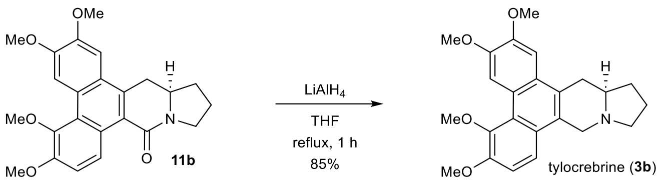

(–)-tylocrebrine (3b)

Note: The procedure used to prepare (–)-antofine (**3a**) with **11b** (32 mg, 0.079 mmol, 1.0 equiv) was followed. The crude mixture was purified by flash chromatography on silica gel (CH_2Cl_2:MeOH = 20:1, *v*/*v*) to obtain (–)-tylocrebrine (**3b**, 26 mg, 85%) as a white solid.

TLC R_f = 0.2 (CH_2Cl_2:MeOH = 20:1, *v*/*v*)

Melting point: 216–221 °C

^{1}H NMR (400 MHz, $CDCl_3$) δ 9.32 (s, 1H), 7.60 (d, *J* = 9.1 Hz, 1H), 7.29 (s, 1H), 7.26 (d, *J* = 9.0 Hz, 1H), 4.65 (d, *J* = 14.8 Hz, 1H), 4.06 (s, 3H), 4.05 (s, 3H), 4.01 (s, 3H), 3.91 (s, 3H), 3.67 (d, *J* = 15.2 Hz, 1H), 3.46 (td, *J* = 8.7, 2.4 Hz, 1H), 3.29 (ddd, *J* = 15.9, 4.1, 1.7 Hz, 1H), 2.92 (dd, *J* = 15.9, 10.5 Hz, 1H), 2.57 – 2.40 (m, 2H), 2.29 – 2.16 (m, 1H), 2.13 – 1.97 (m, 1H), 1.97 – 1.86 (m, 1H), 1.85 – 1.69 (m, 1H)

^{13}C NMR (100 MHz, $CDCl_3$) δ 150.7, 148.7, 147.8, 146.3, 127.9, 126.4, 126.1, 125.7, 123.6, 123.4, 118.9, 112.1, 109.1, 103.5, 60.3, 60.1, 56.5, 55.8, 55.1, 54.0, 33.6, 31.2, 21.6

IR (neat, cm^{-1}): 3000, 2930, 2851, 1515, 1497, 1283, 1256, 1114, 1030, 781

Optical Rotation: $[\alpha]_D^{22}$ –103.3 (*c* 1.0, $CHCl_3$)

HRMS (FAB): *m*/*z* calculated for $C_{24}H_{28}NO_4$ $[M+H]^+$ 394.2018, found 394.2013

2.5 Total synthesis of (–)-isotylocrebrine (3c)

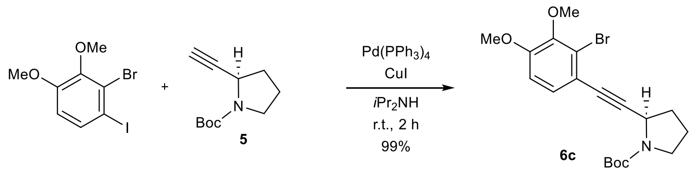

tert-butyl (*R*)-2-((2-bromo-3,4-dimethoxyphenyl)ethynyl)pyrrolidine-1-carboxylate (6c)

To a solution of 2-bromo-1-iodo-3,4-dimethoxybenzene[4] (1.17 g, 3.41 mmol, 1.1 equiv) and **5** (605 mg, 3.10 mmol, 1.0 equiv) in *i*Pr_2NH (6.2 mL), $Pd(PPh_3)_4$ (179 mg, 0.155 mmol, 0.05 equiv) and CuI (59 mg, 0.31 mmol, 0.1 equiv) were added at room temperature. The mixture was stirred at room temperature for 2 h and quenched with saturated NH_4Cl aqueous solution (15 mL) at 0 °C. The mixture was extracted with three times CH_2Cl_2 (3 × 30 mL), and the combined organic fraction was dried over $MgSO_4$ and concentrated under reduced pressure. The crude mixture was purified by flash chromatography on silica gel (hexane:EtOAc = 4:1, *v*/*v*) to obtain compound **6c** (1.26 g, 99%) as a yellow oil.

TLC R_f = 0.2 (hexane:EtOAc = 4:1, *v*/*v*)

^{1}H NMR (400 MHz, $CDCl_3$) δ 7.16 (d, *J* = 8.6 Hz, 1H), 6.78 (d, *J* = 8.6 Hz, 1H), 4.70 (brs, 1H), 3.86 (s, 3H), 3.83 (s, 3H), 3.58 – 3.47 (m, 1H), 3.43 – 3.31 (m, 1H), 2.27 – 2.09 (m, 3H), 2.00 – 1.88 (m, 1H), 1.49 (s, 9H)

^{13}C NMR (100 MHz, $CDCl_3$) δ 154.3, 153.8, 146.9, 128.9, 121.6, 118.5, 111.2, 93.0, 80.1, 79.8, 60.6, 56.2, 49.0, 45.7, (34.0, 33.3), 28.7 (3C), (24.6, 23.9)

IR (neat, cm^{-1}): 2974, 2939, 2881, 2843, 1696, 1486, 1392, 1296, 1166, 1035, 808

Optical Rotation: $[\alpha]_D^{25}$ +123.0 (*c* 0.5, $CHCl_3$)

HRMS (FAB): *m*/*z* calculated for $C_{15}H_{17}BrNO_4$ $[M–C_4H_9]^+$ 354.0341, found 354.0340

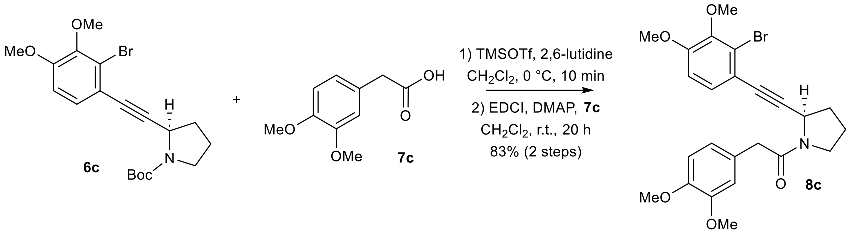

(*R*)-1-(2-((2-bromo-3,4-dimethoxyphenyl)ethynyl)pyrrolidin-1-yl)-2-(3,4-dimethoxyphenyl)ethan-1-one (8c)

Note: The procedure used to prepare **8a** with **6c** (1.23 g, 3.00 mmol, 1.0 equiv) and 2-(3,4-dimethoxyphenyl)acetic acid (**7c**, 705 mg, 3.60 mmol, 1.2 equiv) was followed. The crude mixture was purified by flash chromatography on silica gel (hexane:EtOAc = 1:1, *v*/*v*) to obtain compound **8c** (1.22 g, 83%, 2 steps) as a white wax.

TLC R_f = 0.2 (hexane:EtOAc = 1:1, *v*/*v*)

^{1}H NMR (400 MHz, $CDCl_3$) δ 7.17 (dd, *J* = 12.4, 8.6 Hz, 1H), 6.97 – 6.72 (m, 4H), 5.04 (d, *J* = 6.2 Hz, 0.4H), 4.75 – 4.71 (m, 0.6H), 3.93 – 3.83 (m, 12H), 3.81 (d, *J* = 4.8 Hz, 2H), 3.77 – 3.68 (m, 0.6H), 3.62 – 3.57 (m, 0.4H), 3.53 – 3.35 (m, 1H), 2.47 – 2.22 (m, 1H), 2.22 – 1.88 (m, 3H)

^{13}C NMR (100 MHz, $CDCl_3$) δ (170.4, 169.5), (154.3, 153.8), (149.2, 149.1), (148.1, 148.0), (147.0, 146.8), (129.1, 129.0), (127.7, 127.2), (121.8, 121.8), (121.4, 121.1), (118.2, 117.6), (112.4, 112.1), 111.4, (111.3, 111.1), (92.1, 91.3), (82.5, 80.5), (60.63, 60.56), (56.3, 56.2), 56.03, 55.98, (49.3, 48.6), (46.6, 46.2), (41.9, 41.6), (34.6, 32.6), (25.1, 23.2)

IR (neat, cm^{-1}): 2941, 2881, 2840, 1645, 1515, 1486, 1398, 1294, 1261, 1029, 810

Optical Rotation: $[\alpha]_D^{25}$ +119.2 (*c* 0.5, $CHCl_3$)

HRMS (FAB): *m*/*z* calculated for $C_{24}H_{27}BrNO_5$ $[M+H]^+$ 488.1073, found 488.1082

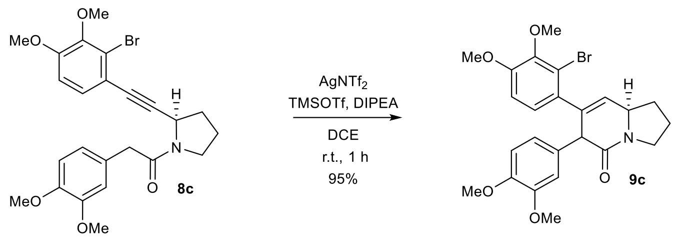

(8a*R*)-7-(2-bromo-3,4-dimethoxyphenyl)-6-(3,4-dimethoxyphenyl)-2,3,6,8a-tetrahydroindolizin-5(1*H*)-one (9c)

Note: The procedure used to prepare **9a** with **8c** (46 mg, 0.096 mmol, 1.0 equiv) was followed. The crude mixture was purified by flash chromatography on silica gel (CH_2Cl_2:EtOAc = 1:1, *v*/*v*) to obtain compound **9c** (44 mg, 95%) as a white wax.

TLC R_f = 0.5 (CH_2Cl_2:EtOAc = 1:1, *v*/*v*)

^{1}H NMR (400 MHz, $CDCl_3$) δ 6.69 – 6.60 (m, 2H), 6.59 – 6.48 (m, 3H), 5.95 (d, *J* = 2.7 Hz, 1H), 4.68 (d, *J* = 5.9 Hz, 1H), 4.37 – 4.15 (m, 1H), 3.95 – 3.81 (m, 1H), 3.79 – 3.74 (m, 9H), 3.73 (s, 3H), 3.53 – 3.39 (m, 1H), 2.33 – 2.21 (m, 1H), 2.16 – 2.06 (m, 1H), 2.05 – 1.92 (m, 1H), 1.84 – 1.64 (m, 1H)

^{13}C NMR (100 MHz, $CDCl_3$) δ 167.7, 152.4, 148.4, 147.7, 146.1, 138.7, 133.8, 131.6, 125.9, 124.8, 121.8, 118.1, 112.5, 110.9, 110.8, 60.5, 58.9, 56.0, 55.82, 55.79, 50.9, 44.2, 32.8, 22.6

IR (neat, cm^{-1}): 2961, 2939, 2840, 1644, 1486, 1447, 1290, 1258, 1029, 811, 754

Optical Rotation: $[\alpha]_D^{25}$ +25.4 (*c* 0.5, $CHCl_3$)

HRMS (FAB): *m*/*z* calculated for $C_{24}H_{27}BrNO_5$ $[M+H]^+$ 488.1073, found 488.1083

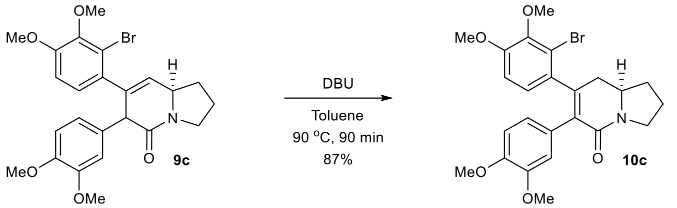

(*R*)-7-(2-bromo-3,4-dimethoxyphenyl)-6-(3,4-dimethoxyphenyl)-2,3,8,8a-tetrahydroindolizin-5(1*H*)-one (10c)

Note: The procedure used to prepare **10a** with **9c** (347 mg, 0.711 mmol, 1.0 equiv) was followed. The crude mixture was purified by flash chromatography on silica gel (CH_2Cl_2:EtOAc = 1:1, *v*/*v*) to obtain compound **10c** (303 mg, 87%) as a white wax.

TLC R_f = 0.7 (CH_2Cl_2:EtOAc = 1:1, *v*/*v*)

^{1}H NMR (400 MHz, $CDCl_3$) δ 6.87 (dd, *J* = 8.3, 2.0 Hz, 1H), 6.74 (s, 1H), 6.68 – 6.63 (m, 1H), 6.61 – 6.51 (m, 1H), 6.47 (dd, *J* = 8.4, 1.5 Hz, 1H), 4.20 – 4.06 (m, 0.7H), 4.00 – 3.89 (m, 0.3H), 3.81 (dd, *J* = 5.2, 1.4 Hz, 3H), 3.77 (d, *J* = 3.0 Hz, 6H), 3.70 – 3.67 (m, 3H), 3.66 – 3.51 (m, 2H), 3.06 (dd, *J* = 16.2, 14.0 Hz, 0.3H), 2.85 (dd, *J* = 16.7, 4.2 Hz, 0.7H), 2.56 – 2.37 (m, 1H), 2.34 – 2.18 (m, 1H), 2.15 – 1.97 (m, 1H), 1.94 – 1.80 (m, 1H), 1.78 – 1.60 (m, 1H)

^{13}C NMR (100 MHz, $CDCl_3$) δ 163.8, 152.4, (147.8, 147.7), 146.6, (145.6, 145.2), (135.8, 134.9), (134.7, 133.9), (128.4, 127.9), (125.4, 124.9), 122.9, 118.5, 117.2, (113.7, 113.4), (111.6, 111.4), (110.3, 110.2), 60.6, 56.3, 56.0, 55.8, 55.6, (45.0, 44.9), (37.5, 36.8), (33.8, 33.7), 23.4

IR (neat, cm^{-1}): 2940, 2880, 2840, 1650, 1433, 1290, 1255, 1142, 1030, 814, 754

Optical Rotation: $[\alpha]_D^{25}$ –109.4 (*c* 0.5, $CHCl_3$)

HRMS (FAB): *m*/*z* calculated for $C_{24}H_{27}BrNO_5$ $[M+H]^+$ 488.1073, found 488.1055

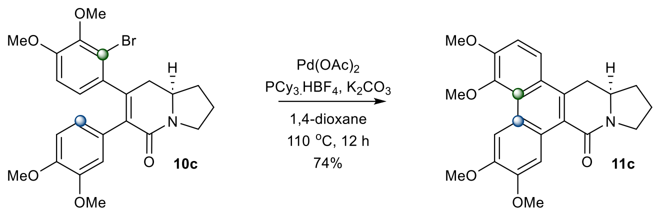

(*R*)-3,4,6,7-tetramethoxy-12,13,13a,14-tetrahydrodibenzo[*f,h*]pyrrolo[1,2-*b*]isoquinolin-9(11*H*)-one (11c)

Note: The procedure used to prepare **11b** with **10c** (26 mg, 0.054 mmol, 1.0 equiv) was followed. The crude mixture was purified by flash chromatography on silica gel (CH_2Cl_2:MeOH = 20:1, *v*/*v*) to obtain compound **11c** (16 mg, 74%) as a white solid.

TLC R_f = 0.2 (CH_2Cl_2:MeOH = 20:1, *v*/*v*)

Melting point: 231–238 °C

^{1}H NMR (400 MHz, $CDCl_3$) δ 9.24 (s, 1H), 8.89 (s, 1H), 7.84 (d, *J* = 9.1 Hz, 1H), 7.29 (d, *J* = 9.1 Hz, 1H), 4.08 (s, 3H), 4.05 (d, *J* = 3.2 Hz, 6H), 3.94 – 3.87 (m, 1H), 3.85 (s, 3H), 3.82 – 3.69 (m, 1H), 3.58 (dd, *J* = 15.9, 4.2 Hz, 1H), 2.88 (dd, *J* = 15.8, 13.3 Hz, 1H), 2.53 – 2.30 (m, 1H), 2.22 – 2.10 (m, 1H), 2.05 – 1.76 (m, 3H)

^{13}C NMR (100 MHz, $CDCl_3$) δ 164.5, 152.5, 148.9, 148.0, 145.9, 134.1, 126.2, 125.9, 124.8, 124.0, 123.3, 121.4, 112.0, 108.1, 107.6, 60.3, 56.5, 55.9, 55.8, 55.3, 45.5, 33.9, 32.8, 23.7

IR (neat, cm^{-1}): 2959, 2932, 2878, 2843, 1633, 1511, 1446, 1251, 1117, 1052, 752

Optical Rotation: $[\alpha]_D^{25}$ –334.6 (*c* 0.5, $CHCl_3$)

HRMS (FAB): *m*/*z* calculated for $C_{24}H_{26}NO_5$ $[M+H]^+$ 408.1811, found 408.1809

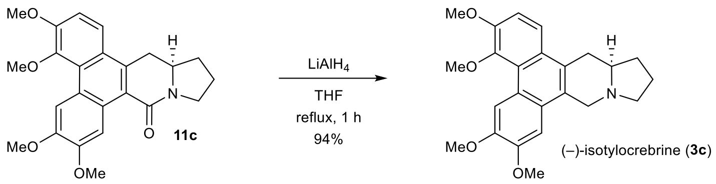

(–)-isotylocrebrine (3c)

Note: The procedure used to prepare **3a** with **11c** (11 mg, 0.027 mmol, 1.0 equiv) was followed. The crude mixture was purified by flash chromatography on silica gel (CH_2Cl_2:MeOH = 20:1, *v*/*v*) to obtain (–)-isotylocrebrine (**3c**, 9.9 mg, 94%) as a white solid.

TLC R_f = 0.2 (CH_2Cl_2:MeOH = 20:1, *v*/*v*)

Melting point: 201–207 °C

^{1}H NMR (400 MHz, $CDCl_3$) δ 9.32 (s, 1H), 7.76 (d, *J* = 9.1 Hz, 1H), 7.27 (d, *J* = 9.3 Hz, 1H), 7.11 (s, 1H), 4.59 (d, *J* = 14.4 Hz, 1H), 4.06 (s, 3H), 4.04 (s, 3H), 4.02 (s, 3H), 3.90 (s, 3H), 3.66 (d, *J* = 14.7 Hz, 1H), 3.48 (td, *J* = 8.8, 2.5 Hz, 1H), 3.34 (ddd, *J* = 16.1, 4.1, 1.7 Hz, 1H), 2.95 (dd, *J* = 16.1, 10.4 Hz, 1H), 2.61 – 2.39 (m, 2H), 2.30 – 2.15 (m, 1H), 2.13 – 1.97 (m, 1H), 1.99 – 1.85 (m, 1H), 1.85 – 1.69 (m, 1H)

^{13}C NMR (100 MHz, $CDCl_3$) δ 150.8, 148.8, 147.8, 146.1, 127.1, 126.8, 126.3, 125.5, 123.9, 123.2, 119.9, 112.0, 109.3, 102.6, 60.3, 60.1, 56.5, 55.9, 55.8, 55.1, 53.8, 33.5, 31.1, 21.6

IR (neat, cm^{-1}): 2936, 2835, 2794, 1514, 1467, 1281, 1255, 1113, 1031, 761

Optical Rotation: $[\alpha]_D^{25}$ –70.2 (*c* 0.5, $CHCl_3$)

HRMS (FAB): *m*/*z* calculated for $C_{24}H_{28}NO_4$ $[M+H]^+$ 394.2018, found 394.2019

2.6 Total synthesis of (–)-cryptopleurine (4a)

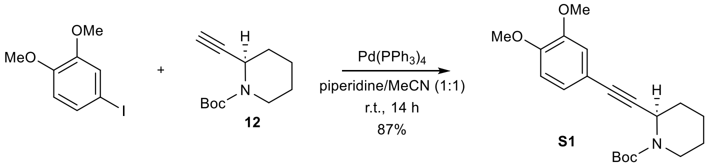

***tert*-butyl (*R*)-2-((3,4-dimethoxyphenyl)ethynyl)piperidine-1-carboxylate (S1)**

Note: The procedure used to prepare **6a** with 4-iodo-1,2-dimethoxybenzene (1.57 g, 5.94 mmol, 1.1 equiv) and **12**[5] (1.13 g, 5.40 mmol, 1.0 equiv) was followed. The crude mixture was purified by flash chromatography on silica gel (hexane:EtOAc = 5:1, *v*/*v*) to obtain compound **S1** (1.63 g, 87%) as a yellow oil.

TLC R_f = 0.2 (hexane:EtOAc = 5:1, *v*/*v*)

^{1}H NMR (400 MHz, $CDCl_3$) δ 7.02 (dd, *J* = 8.3, 1.9 Hz, 1H), 6.91 (d, *J* = 1.9 Hz, 1H), 6.78 (d, *J* = 8.3 Hz, 1H), 5.28 (s, 1H), 3.95 (d, *J* = 13.4 Hz, 1H), 3.87 (d, *J* = 1.1 Hz, 6H), 3.11 (t, *J* = 13.0 Hz, 1H), 1.89 – 1.80 (m, 2H), 1.74 – 1.63 (m, 3H), 1.48 (s, 9H), 1.44 – 1.35 (m, 1H)

^{13}C NMR (100 MHz, $CDCl_3$) δ 154.7, 149.4, 148.6, 125.1, 115.4, 114.5, 111.0, 86.2, 84.1, 80.0, 56.01, 56.00, 44.9, 40.6, 30.9, 28.6 (3C), 25.5, 20.3

IR (neat, cm^{-1}): 3004, 2939, 2864, 1694, 1514, 1409, 1244, 1160, 1027, 856, 763

Optical Rotation: $[\alpha]_D^{25}$ +96.7 (*c* 0.5, $CHCl_3$)

HRMS (FAB): *m*/*z* calculated for $C_{16}H_{20}NO_4$ $[M–C_4H_9]^+$ 290.1393, found 290.1398

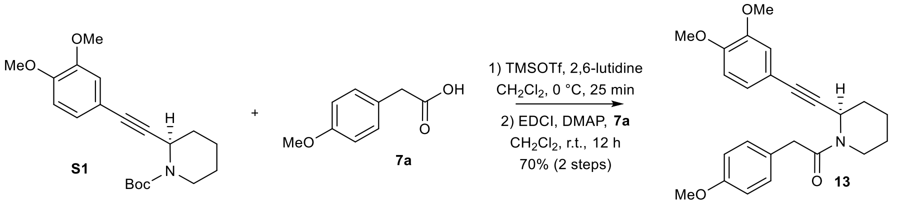

(*R*)-1-(2-((3,4-dimethoxyphenyl)ethynyl)piperidin-1-yl)-2-(4-methoxyphenyl)ethan-1-one (13)

Note: The procedure used to prepare **8a** with **S1** (430 mg, 1.24 mmol, 1.0 equiv) and **7a** (248 mg, 1.49 mmol, 1.2 equiv) was followed. The crude mixture was purified by flash chromatography on silica gel (hexane:EtOAc = 3:1, *v*/*v*) to obtain compound **13** (340 mg, 70%) as a white wax.

TLC R_f = 0.1 (hexane:EtOAc = 3:1, *v*/*v*)

^{1}H NMR (400 MHz, $CDCl_3$) δ 7.18 (dd, *J* = 15.7, 8.0 Hz, 2H), 7.05 – 6.95 (m, 1H), 6.93 – 6.81 (m, 3H), 6.78 (d, *J* = 8.3 Hz, 1H), 5.93 – 5.82 (m, 0.6H), 5.04 – 4.93 (m, 0.4H), 4.53 (d, *J* = 13.4 Hz, 0.4H), 3.87 (s, 6H), 3.77 (s, 3H), 3.74 – 3.69 (m, 0.6H), 3.68 (s, 2H), 3.44 – 3.35 (m, 0.6H), 3.04 – 2.90 (m, 0.4H), 2.08 – 1.52 (m, 5H), 1.47 – 1.07 (m, 1H)

^{13}C NMR (100 MHz, $CDCl_3$) δ 169.6, 158.4, 149.4, 148.6, 129.7 (2C), 127.1, 125.1, 115.2 114.5, 114.2 (2C), 110.9, (85.8, 85.1), (84.7, 84.1), (56.00, 55.98), 55.3, 47.3, 43.1, 42.4, (40.4, 38.5), (31.4, 30.6), (25.9, 25.2), 20.2

IR (neat, cm^{-1}): 3004, 2939, 2863, 2839, 1642, 1512, 1411, 1242, 1024, 814

Optical Rotation: $[\alpha]_D^{25}$ +88.4 (*c* 0.5, $CHCl_3$)

HRMS (FAB): *m*/*z* calculated for $C_{24}H_{28}NO_4$ $[M+H]^+$ 394.2018, found 394.2012

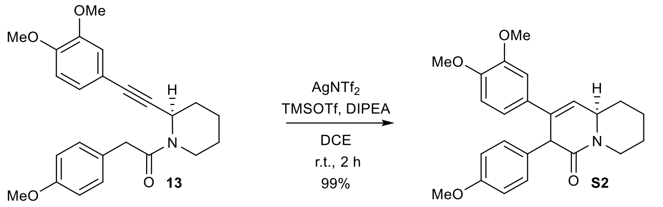

(9a*R*)-2-(3,4-dimethoxyphenyl)-3-(4-methoxyphenyl)-3,6,7,8,9,9a-hexahydro-4*H*-quinolizin-4-one (S2)

Note: The procedure used to prepare **9a** with **13** (91 mg, 0.23 mmol, 1.0 equiv) was followed. The crude mixture was purified by flash chromatography on silica gel (CH_2Cl_2:EtOAc = 1:1, *v*/*v*) to obtain compound **S2** (90 mg, 99%) as a white wax.

TLC R_f = 0.5 (CH_2Cl_2:EtOAc = 1:3, *v*/*v*)

^{1}H NMR (400 MHz, $CDCl_3$) δ 7.32 – 7.27 (m, 2H), 6.83 (dd, *J* = 8.3, 2.2 Hz, 1H), 6.80 – 6.74 (m, 3H), 6.69 (d, *J* = 8.4 Hz, 1H), 6.15 (d, *J* = 4.0 Hz, 1H), 4.66 (ddt, *J* = 13.1, 4.1, 2.0 Hz, 1H), 4.50 (d, *J* = 3.0 Hz, 1H), 4.02 – 3.94 (m, 1H), 3.77 (s, 3H), 3.73 (s, 3H), 3.70 (s, 3H), 2.51 (td, *J* = 13.0, 2.7 Hz, 1H), 2.12 – 2.03 (m, 1H), 1.90 (dd, *J* = 12.3, 4.6 Hz, 1H), 1.67 – 1.41 (m, 3H), 1.34 – 1.19 (m, 1H)

^{13}C NMR (100 MHz, $CDCl_3$) δ 167.5, 158.7, 148.5, 134.1, 131.7, 131.0, 129.3 (2C), 121.6, 118.6, 114.1 (2C), 110.9, 109.2, 58.7, 55.8, 55.7, 55.2, 49.3, 42.6, 34.1, 25.3, 24.5

IR (neat, cm^{-1}): 3002, 2936, 2840, 1634, 1510, 1464, 1240, 1172, 1026, 758

Optical Rotation: $[\alpha]_D^{25}$ +33.5 (*c* 0.5, $CHCl_3$)

HRMS (FAB): *m*/*z* calculated for $C_{24}H_{28}NO_4$ $[M+H]^+$ 394.2018, found 394.2008

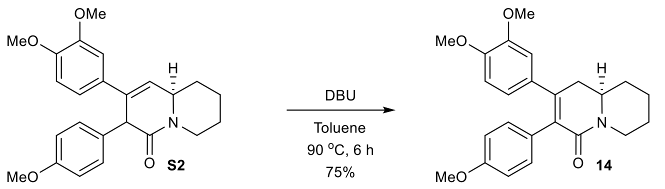

(*R*)-2-(3,4-dimethoxyphenyl)-3-(4-methoxyphenyl)-1,6,7,8,9,9a-hexahydro-4*H*-quinolizin-4-one (14)

Note: The procedure used to prepare **10a** with **S2** (26 mg, 0.066 mmol, 1.0 equiv) was followed. The crude mixture was purified by flash chromatography on silica gel (CH_2Cl_2:EtOAc = 2:1, *v*/*v*) to obtain compound **14** (19 mg, 75%) as a white wax.

TLC R_f = 0.8 (CH_2Cl_2:EtOAc = 2:1, *v*/*v*)

^{1}H NMR (400 MHz, $CDCl_3$) δ 6.99 (d, *J* = 8.5 Hz, 2H), 6.76 – 6.71 (m, 4H), 6.40 (d, *J* = 1.9 Hz, 1H), 4.55 (d, *J* = 13.5 Hz, 1H), 3.83 (s, 3H), 3.74 (s, 3H), 3.62 – 3.53 (m, 1H), 3.47 (s, 3H), 2.86 (dd, *J* = 17.2, 5.5 Hz, 1H), 2.73 (dd, *J* = 17.2, 10.8 Hz, 1H), 2.65 (td, *J* = 12.9, 3.1 Hz, 1H), 1.84 (dd, *J* = 29.8, 11.9 Hz, 3H), 1.63 – 1.35 (m, 3H)

^{13}C NMR (100 MHz, $CDCl_3$) δ 167.0, 158.4, 148.5, 147.9, 144.5, 132.5, 132.3 (2C), 130.5, 129.4, 120.8, 113.4 (2C), 112.8, 110.4, 55.8, 55.6, 55.3, 54.0, 43.4, 37.3, 33.5, 24.9, 23.7

IR (neat, cm^{-1}): 2998, 2936, 2839, 1641, 1608, 1463, 1174, 1027, 829

Optical Rotation: $[\alpha]_D^{25}$ –16.4 (*c* 0.5, $CHCl_3$)

HRMS (FAB): *m*/*z* calculated for $C_{24}H_{28}NO_4$ $[M+H]^+$ 394.2018, found 394.2027

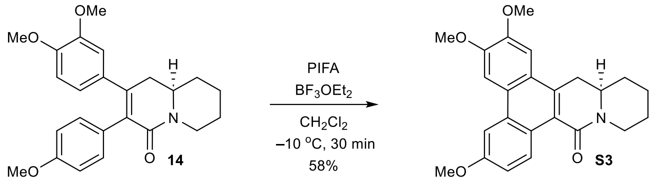

(*R*)-2,3,6-trimethoxy-11,12,13,14,14a,15-hexahydro-9*H*-dibenzo[*f*,*h*]pyrido[1,2-*b*]isoquinolin-9-one (S3)

Note: The procedure used to prepare **11a** with **14** (79 mg, 0.20 mmol, 1.0 equiv) was followed. The crude mixture was purified by flash chromatography on silica gel (CH_2Cl_2:MeOH = 20:1, *v*/*v*) to obtain compound **S3** (46 mg, 58%) as a white solid.

TLC R_f = 0.4 (CH_2Cl_2:MeOH = 20:1, *v*/*v*)

Melting point: 187–193 °C

^{1}H NMR (400 MHz, $CDCl_3$) δ 9.57 (d, *J* = 9.5 Hz, 1H), 7.88 (s, 1H), 7.85 (d, *J* = 2.7 Hz, 1H), 7.30 (s, 1H), 7.26 – 7.21 (m, 1H), 4.76 – 4.70 (m, 1H), 4.12 (s, 3H), 4.06 (s, 3H), 4.01 (s, 3H), 3.63 – 3.52 (m, 1H), 3.39 (dd, *J* = 16.3, 4.6 Hz, 1H), 2.97 (dd, *J* = 16.3, 11.1 Hz, 1H), 2.92 – 2.81 (m, 1H), 2.03 (d, *J* = 13.4 Hz, 1H), 1.90 (d, *J* = 11.3 Hz, 2H), 1.74 – 1.57 (m, 2H), 1.56 – 1.39 (m, 1H)

^{13}C NMR (100 MHz, $CDCl_3$) δ 167.2, 157.5, 150.2, 149.4, 132.9, 131.1, 130.1, 126.5, 123.9, 123.8, 121.0, 115.1, 104.5, 104.2, 103.6, 56.0, 55.9, 55.5, 52.5, 42.6, 33.0, 32.9, 24.7, 23.0

IR (neat, cm^{-1}): 3002, 2935, 2855, 1615, 1510, 1417, 1252, 1203, 1040, 838, 750

Optical Rotation: $[\alpha]_D^{25}$ –58.2 (*c* 0.5, $CHCl_3$)

HRMS (FAB): *m*/*z* calculated for $C_{24}H_{26}NO_4$ $[M+H]^+$ 392.1862, found 392.1857

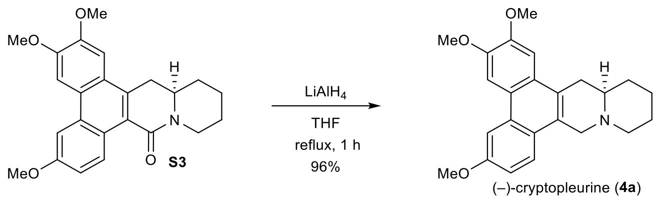

(–)-cryptopleurine (4a)

Note: The procedure used to prepare **3a** with **S3** (36 mg, 0.092 mmol, 1.0 equiv) was followed. The crude mixture was purified by flash chromatography on silica gel (CH_2Cl_2:MeOH = 20:1, *v*/*v*) to obtain (–)-cryptopleurine (**4a,** 33 mg, 96%) as a white solid.

TLC R_f = 0.2 (CH_2Cl_2:MeOH = 15:1, *v*/*v*)

Melting point: 189–194 °C

^{1}H NMR (400 MHz, $CDCl_3$) δ 7.87 (s, 1H), 7.86 (d, *J* = 2.8 Hz, 1H), 7.72 (d, *J* = 9.0 Hz, 1H), 7.20 (s, 1H), 7.18 (dd, *J* = 9.0, 2.5 Hz, 1H), 4.53 (d, *J* = 15.5 Hz, 1H), 4.09 (s, 3H), 4.05 (s, 3H), 4.00 (s, 3H), 3.85 – 3.68 (m, 1H), 3.37 (d, *J* = 11.3 Hz, 1H), 3.17 – 2.96 (m, 2H), 2.69 – 2.41 (m, 2H), 2.15 – 2.02 (m, 1H), 1.98 – 1.81 (m, 3H), 1.78 – 1.63 (m, 1H), 1.59 – 1.43 (m, 1H)

^{13}C NMR (100 MHz, $CDCl_3$) δ 157.7, 149.5, 148.6, 130.2, 126.0, 123.92 (2C), 123.86, 123.5, 123.1, 115.1, 104.8, 103.8 (2C), 57.6, 56.1, 56.0, 55.8, 55.6, 55.2, 33.4, 32.6, 25.0, 23.6

IR (neat, cm^{-1}): 3005, 2940, 2845, 1612, 1515, 1422, 1260, 1206, 1039, 784

Optical Rotation: $[\alpha]_D^{25}$ –95.5 (*c* 0.4, $CHCl_3$)

HRMS (FAB): *m*/*z* calculated for $C_{24}H_{28}NO_3$ $[M+H]^+$ 378.2069, found 378.2075

2.7 Data comparison of natural products

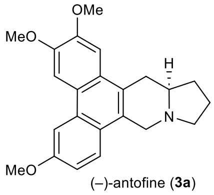

(–)-antofine (**3a**)

$[\alpha]_D^{25}$ –124.0 (c 0.15, $CHCl_3$)

lit. $[\alpha]_D^{25}$ –123.9 (c 0.15, $CHCl_3$)[6]

^{1}H NMR		^{13}C NMR	
Natural (300 MHz, $CDCl_3$)[6]	Synthetic (Kim) (400 MHz, $CDCl_3$)	Natural (75 MHz, $CDCl_3$)[6]	Synthetic (Kim) (100 MHz, $CDCl_3$)
7.91 (s, 1H)	7.85 (s, 1H)	157.75	157.5
7.90 (d, *J* = 2.6 Hz, 1H)	7.84 (d, *J* = 2.8 Hz, 1H)	149.63	149.4
7.83 (d, *J* = 9.1 Hz, 1H)	7.74 (d, *J* = 9.0 Hz, 1H)	148.60	148.4
7.31 (s, 1H)	7.23 (s, 1H)	130.39	130.2
7.21 (dd, *J* = 9.1, 2.6 Hz, 1H)	7.16 (dd, *J* = 9.0, 2.5 Hz, 1H)	127.24	127.0
4.74 (d, *J* = 15.1 Hz, 1H)	4.65 (dd, *J* = 15.1, 1.3 Hz, 1H)	126.68	126.3
4.11 (s, 3H)	4.08 (s, 3H)	125.72	125.5
4.07 (s, 3H)	4.03 (s, 3H)	124.48	124.3
4.02 (s, 3H)	3.98 (s, 3H)	124.28	124.1
3.72 (d, *J* = 15.1Hz, 1H)	3.64 (dt, *J* = 15.0, 2.2 Hz, 1H)	123.74	123.6
3.47 (dt, *J* = 8.7, 2.0 Hz, 1H)	3.45 (td, *J* = 8.7, 2.4 Hz, 1H)	115.08	114.9
3.35 (ddd, *J* = 15.6, 3.8, 1.4 Hz, 1H)	3.25 (ddd, *J* = 15.9, 4.1, 1.7 Hz, 1H)	104.84	104.7
2.92 (dd, *J* = 15.6, 10.5 Hz, 1H)	2.94 – 2.80 (m, 1H)	104.11	104.0
2.53 (m, 1H)	2.51 – 2.37 (m, 2H)	103.94	103.8
2.51 (q, *J* = 8.7 Hz, 1H)		60.35	60.3
2.25 (m, 1H)	2.27 – 2.12 (m, 1H)	56.10	56.1
2.03 (m, 1H)	2.11 – 1.94 (m, 1H)	55.98	55.9
1.93 (m, 1H)	1.97 – 1.81 (m, 1H)	55.14	55.6
1.80 (m, 1H)	1.83 – 1.67 (m, 1H)	55.13	55.0
		53.82	53.7
		33.63	33.4
		31.28	31.2
		21.61	21.6

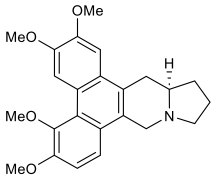

(–)-tylocrebrine (**3b**)

$[\alpha]_D^{22}$ –103.3 (c 1.0, $CHCl_3$)

lit. $[\alpha]_D^{22}$ –105.0 (c 1.0, $CHCl_3$)[7]

^{1}H NMR		^{13}C NMR	
Synthetic (Georg) (400 MHz, $CDCl_3$)[7]	Synthetic (Kim) (400 MHz, $CDCl_3$)[a]	Synthetic (Georg) (100 MHz, $CDCl_3$)[7]	Synthetic (Kim) (100 MHz, $CDCl_3$)[a]
9.33 (s, 1H)	9.32 (s, 1H)	150.6	150.7
7.65 (d, *J* = 9.1 Hz, 1H)	7.60 (d, *J* = 9.1 Hz, 1H)	148.7	148.7
7.33 (s, 1H)	7.29 (s, 1H)	147.7	147.8
7.28 (d, *J* = 9.1 Hz, 1H)	7.26 (d, *J* = 9.0 Hz, 1H)	146.3	146.3
4.66 (d, *J* = 14.8 Hz, 1H)	4.65 (d, *J* = 14.8 Hz, 1H)	127.9	127.9
4.07 (s, 3H)	4.06 (s, 3H)	126.5	126.4
4.07 (s, 3H)	4.05 (s, 3H)	126.5	126.1
4.03 (s, 3H)	4.01 (s, 3H)	125.8	125.7
3.92 (s, 3H)	3.91 (s, 3H)	123.6	123.6
3.68 (d, *J* = 14.7 Hz, 1H)	3.67 (d, *J* = 15.2 Hz, 1H)	123.3	123.4
3.46 (dd, *J* = 8.2, 8.2 Hz, 1H)	3.46 (td, *J* = 8.7, 2.4 Hz, 1H)	118.8	118.9
3.33 (dd, *J* = 15.8, 3.5 Hz, 1H)	3.29 (ddd, *J* = 15.9, 4.1, 1.7 Hz, 1H)	112.1	112.1
2.91 (dd, *J* = 14.8, 11.3 Hz, 1H),	2.92 (dd, *J* = 15.9, 10.5 Hz, 1H)	109.1	109.1
2.52 – 2.42 (m, 2H)	2.57 – 2.40 (m, 2H)	103.5	103.5
2.31 – 2.19 (m, 1H)	2.29 – 2.16 (m, 1H)	60.2	60.3
2.10 – 1.99 (m, 1H)	2.13 – 1.97 (m, 1H)	60.0	60.1
1.96 – 1.87 (m, 1H)	1.97 – 1.86 (m, 1H)	56.5	56.5
1.83 – 1.73 (m, 1H)	1.85 – 1.69 (m, 1H)	55.7	55.8
		55.1	55.1
		54.2	54.0
		34.0	33.6
		31.3	31.2
		21.6	21.6

[a] For data comparision, reference (^{1}H NMR, $CDCl_3$, 7.26 ppm/^{13}C NMR, $CDCl_3$, 77.2 ppm) as same as reported paper.[7]

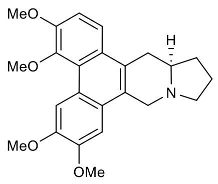

(-)-isotylocrebrine (**3c**)

$[\alpha]_D^{26}$ –25.2 (c 0.5, $CHCl_3$)

lit. $[\alpha]_D^{26}$ +20.2 (c 0.5, $CHCl_3$)[a],8

^{1}H NMR		^{13}C NMR	
Natural (400 MHz, $CDCl_3$)[8]	Synthetic (Kim) (400 MHz, $CDCl_3$)	Natural (100 MHz, $CDCl_3$)[8]	Synthetic (Kim) (100 MHz, $CDCl_3$)
9.33 (s, 1H)	9.32 (s, 1H)	150.5	150.8
7.81 (d, *J* = 9 Hz, 1H)	7.76 (d, *J* = 9.1 Hz, 1H)	148.4	148.8
7.34 (d, *J* = 9 Hz, 1H)	7.27 (d, *J* = 9.3 Hz, 1H)	147.5	147.8
7.17 (s, 1H)	7.11 (s, 1H)	145.8	146.1
4.60 (d, *J* = 15 Hz, 1H)	4.59 (d, *J* = 14.4 Hz, 1H)	126.6	127.1
4.07 (s, 3H)	4.06 (s, 3H)	126.4	126.8
4.06 (s, 3H)	4.04 (s, 3H)	125.9	126.3
4.05 (s, 3H)	4.02 (s, 3H)	125.0	125.5
3.94 (s, 3H)	3.90 (s, 3H)	123.4	123.9
3.69 (d, *J* = 15 Hz, 1H)	3.66 (d, *J* = 14.7 Hz, 1H)	122.9	123.2
3.46 (td, *J* = 9, 3 Hz, 1H)	3.48 (td, *J* = 8.8, 2.5 Hz, 1H)	119.5	119.9
3.40 (dd, *J* = 16, 2 Hz, 1H)	3.34 (ddd, *J* = 16.1, 4.1, 1.7 Hz, 1H)	112.0	112.0
2.95 (dd, *J* = 16, 10 Hz, 1H)	2.95 (dd, *J* = 16.1, 10.4 Hz, 1H)	109.1	109.3
2.56 (m, 1H)	2.61 – 2.39 (m, 2H)	102.5	102.6
2.53 (q, *J* = 9 Hz, 1H)		60.1	60.3
2.31 (m, 1H)	2.30 – 2.15 (m, 1H)	59.6	60.1
2.07-1.94 (m, 2H)	2.13 – 1.97 (m, 1H)	56.0	56.5
	1.99 – 1.85 (m, 1H)	55.3	55.9
1.79 (m, 1H)	1.85 – 1.69 (m, 1H)	55.3	55.8
		54.5	55.1
		53.4	53.8
		32.9	33.5
		30.4	31.1
		20.9	21.6

[a] Since there was no reported optical rotation for (–)-isotylocrebrine, we compared the optical rotation of (+)-isotylocrebrine as reported paper.[8]

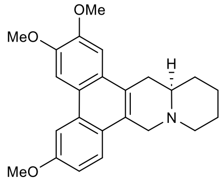

(–)-cryptopleurine (**4a**)

$[\alpha]_D^{25}$ –95.5 (c 0.4, $CHCl_3$)
lit. $[\alpha]_D^{25}$ –96.7 (c 0.4, $CHCl_3$)[9]

^1H NMR		^{13}C NMR	
Synthetic (Kibayashi) (300 MHz, $CDCl_3$)[9]	Synthetic (Kim) (400 MHz, $CDCl_3$)[a]	Synthetic (Kibayashi) (75 MHz, $CDCl_3$)[9]	Synthetic (Kim) (100 MHz, $CDCl_3$)[a]
7.92 (s, 1H)	7.87 (s, 1H)	157.6	157.7
7.90 (d, *J* = 2.6 Hz, 1H)	7.86 (d, *J* = 2.8 Hz, 1H)	149.6	149.5
7.80 (d, *J* = 9.0 Hz, 1H)	7.72 (d, *J* = 9.0 Hz, 1H)	148.5	148.6
7.27 (s, 1H)	7.20 (s, 1H)	130.2	130.2
7.20 (dd, *J* = 9.0, 2.6 Hz, 1H)	7.18 (d, *J* = 9.0, 2.5 Hz, 1H)	126.6	126.0
4.45 (d, *J* = 15.5 Hz, 1H)	4.53 (d, *J* = 15.5 Hz, 1H)	125.7	123.9
4.10 (s, 3H)	4.09 (s, 3H)	124.6	123.9
4.06 (s, 3H)	4.05 (s, 3H)	124.1	123.8
4.01 (s, 3H)	4.00 (s, 3H)	123.8	123.5
3.65 (d, *J* = 15.5 Hz, 1H)	3.85-3.68 (m, 1H)	123.6	123.1
3.28 (d, *J* = 11.4 Hz, 1H)	3.37 (d, *J* = 11.3 Hz, 1H)	114.9	115.1
3.11 (dd, *J* = 16.4, 2.8 Hz, 1H)	3.17-2.96 (m, 2H)	104.9	104.8
2.92 (dd, *J* = 16.4, 10.5 Hz, 1H)		104.2	103.8
2.41 (m, 1H)	2.69-2.41 (m, 2H)	104.1	103.8
2.32 (m, 1H)		57.7	57.6
2.05 (m, 1H)	2.15-2.02 (m, 1H)	56.3	56.1
1.93-1.76 (m, 2H)	1.98-1.81 (m, 3H)	56.2	56.0
1.61-1.40 (m, 2H)	1.78-1.63 (m, 1H)	56.1	55.8
	1.59-1.43 (m, 1H)	56.0	55.6
		55.6	55.2
		34.8	33.4
		33.9	32.6
		26.0	25.0
		24.4	23.6

[a] For data comparision, reference (^1H NMR, $CDCl_3$, 7.26 ppm/^{13}C NMR, $CDCl_3$, 77.2 ppm) as same as reported paper.[9]

3 Computational Studies

3.1 General procedure for molecular energy calculations

Computational energy minimization was performed for systems **9a** and **10a** using the DMol3 program[10] in Material Studio 2022 (Accelrys Software Inc., San Diego, CA, USA). A generalized gradient approximation (GGA) for the Perdew, Burke, and Ernzerhof (PBE)[11] exchange-correlation function was applied with double-numerical plus d-function polarization (DNP), as implemented in DMol3. All the molecules were modeled in the solvent phase (1,2-dichloroethane, COSMOS).

3.2 Geometry optimization and energy minimization of the compounds

The energy levels of the compounds were calculated as described above. This computational energy minimization demonstrated that **10a** is thermodynamically more stable than **9a**. The energy differences was 3.95 kcal/mol.

Table S4. Molecular energy of 9a and 10a

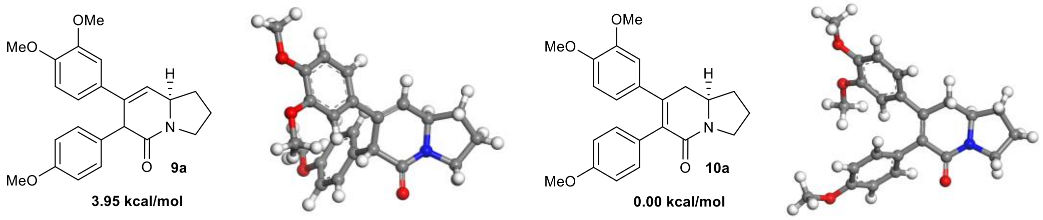

compound	Hartree (Ha)[a]	kcal/mol	Relative energy (kcal/mol)
**9a**	-1246.0712358	-781921.40232	3.95406
**10a**	-1246.0775370	-781925.35638	0

[a] 1Ha = 627.509391 kcal/mol

3.3 Calculation input

All calculations are performed under following conditions.

```
# Task parameters
Calculate                    optimize
Opt_energy_convergence       1.0000e-05
Opt_gradient_convergence     2.0000e-03 A
Opt_displacement_convergence  5.0000e-03 A
Opt_iterations               50
Opt_max_displacement         0.3000 A
Initial_hessian              improved
Symmetry                     on
Max_memory                   2048
File_usage                   smart
Scf_density_convergence      1.000000e-06
Scf_charge_mixing            2.000000e-01
Scf_diis                     6 pulay
Scf_iterations               50

# Electronic parameters
Spin_polarization            restricted
Charge                       0
Basis                        dnp
Pseudopotential              none
Functional                   pbe
Aux_density                  hexadecapole
Integration_grid             fine
Occupation                   fermi
Cutoff_Global                3.7000 angstrom
Cosmo                        ibs
COSMO_Dielectric             10.3600

# Calculated properties
```

Table S5. Cartesian coordinates of the structures

9a

Atom	X	Y	Z	Atom	X	Y	Z
C	-3.459378	-0.774402	-2.223376	H	5.604803	-3.118742	-0.050860
C	-3.120677	-1.679998	-1.215719	H	4.883132	-2.879937	1.552989
C	-1.830834	-1.691936	-0.669722	H	5.126623	-0.838115	-0.711726
C	-0.845648	-0.801830	-1.118162	H	5.206993	-0.566361	1.041227
C	-1.197836	0.123718	-2.127808	C	-0.248867	1.443281	3.055616
C	-2.477277	0.145333	-2.681343	C	-0.107284	2.837134	3.030968
C	0.524404	-0.838790	-0.554940	C	0.453565	3.458454	1.900966
C	1.256170	0.474798	-0.360438	C	0.867111	2.689043	0.818513
C	0.735706	1.288244	0.826849	H	-1.606117	-2.390962	0.136234
C	0.172447	0.684920	1.955990	H	-0.689831	0.939847	3.914814
C	2.780996	0.326793	-0.243453	O	-0.484847	3.676629	4.049511
N	3.251699	-0.843727	0.243723	H	0.554858	4.544843	1.888754
C	2.447128	-2.057896	0.476489	H	1.306871	3.179382	-0.052149
C	1.107013	-1.992546	-0.174759	C	-1.927325	1.962585	-4.138690
C	3.397212	-3.169721	-0.000482	C	-5.696751	-1.602632	-2.376691
C	4.761939	-2.660596	0.482169	C	-1.054207	3.071875	5.221748
C	4.698207	-1.140270	0.254463	H	-1.062774	1.464165	-4.604091
O	3.536175	1.263491	-0.568261	H	-1.584430	2.615427	-3.322227
O	-4.685896	-0.683126	-2.821033	H	-2.453695	2.559983	-4.889878
H	-3.865350	-2.379443	-0.838084	H	-6.582622	-1.378932	-2.980863
H	-0.452429	0.823937	-2.500157	H	-5.929074	-1.452164	-1.311764
O	-2.887466	1.004665	-3.665294	H	-5.388676	-2.644997	-2.546197
H	0.050184	-0.399588	1.987821	H	-1.275369	3.899585	5.903326
H	2.308265	-2.183749	1.570592	H	-0.341084	2.382155	5.697543
H	0.591070	-2.946575	-0.302507	H	-1.983843	2.533963	4.980235
H	3.124869	-4.148401	0.414769	H	1.129765	1.101469	-1.255810
H	3.362383	-3.230284	-1.098826				

Total energy = -1246.0712358 Ha

10a

Atom	X	Y	Z	Atom	X	Y	Z
C	-3.781471	-0.053977	0.427265	H	2.657301	1.391863	4.504168
C	-3.254112	-0.311759	1.694061	H	4.840615	2.512562	4.212326
C	-1.883892	-0.158617	1.940786	H	4.181184	3.551233	2.930352
C	-1.000350	0.226251	0.920130	H	4.868073	0.572673	2.757543
C	-1.542083	0.491233	-0.359778	H	5.096712	1.829201	1.522088
C	-2.902499	0.359324	-0.613775	C	0.430702	-2.467150	-2.285097
C	0.439352	0.400363	1.203613	C	0.913286	-1.876462	-3.462040
C	1.453359	0.100429	0.333713	C	1.577424	-0.638924	-3.394453
C	1.269368	-0.580388	-0.973216	C	1.762371	-0.014029	-2.165227
C	0.614064	-1.816972	-1.060475	H	-1.508941	-0.364029	2.943346
C	2.887187	0.342754	0.719834	H	-0.077027	-3.430022	-2.306977
N	3.094011	1.167297	1.776060	O	0.788500	-2.422425	-4.714781
C	2.006994	1.855820	2.493314	H	1.947691	-0.185935	-4.315229
C	0.798429	0.944525	2.578326	H	2.278150	0.946758	-2.130706
C	2.681613	2.247938	3.811985	C	-2.632435	0.969005	-2.912110
C	4.126707	2.548713	3.380028	C	-6.005245	-0.604415	1.110318
C	4.423681	1.479625	2.317089	C	0.105736	-3.682597	-4.818481
O	3.838578	-0.199468	0.115349	H	-1.908924	0.165387	-3.116385
O	-5.099977	-0.157351	0.086083	H	-2.095402	1.908212	-2.709140
H	-3.908652	-0.626456	2.504872	H	-3.290861	1.103539	-3.776532
H	-0.877968	0.817602	-1.155551	H	-6.995079	-0.613509	0.643307
O	-3.498035	0.615452	-1.820447	H	-6.004594	0.085748	1.966594
H	0.238194	-2.287787	-0.150688	H	-5.744715	-1.618119	1.450051
H	1.731832	2.769837	1.932289	H	0.111838	-3.936999	-5.883501
H	-0.046395	1.503186	3.002293	H	-0.933337	-3.600561	-4.465144
H	1.014545	0.107065	3.265941	H	0.627846	-4.466038	-4.249577
H	2.186648	3.100396	4.296187				

Total energy = -1246.0775370 Ha

4 References

(1) Y. Shao, F. Zhang, J. Zhang, X. Zhou, *Angew. Chem. Int. Ed.* **2016**, *55*, 11485–11489.

(2) J. L. Woodring, R. Behera, A. Sharma, J. Wiedeman, G. Patel, B. Singh, P. Guyett, E. Amata, J. Erath, N. Roncal, E. Penn, S. E. Leed, A. Rodriguez, R. J. Sciotti, K. Mensa-Wilmot, M. P. Pollastri, *ACS Med. Chem. Lett.* **2018**, *9*, 996–1001.

(3) K. Orito, M. Miyazawa, R. Kanbayashi, M. Tokuda, H. Suginome, *J. Org. Chem.* **1999**, *64*, 6 583–6596.

(4) A. Monopoli, M. Casiello, C. Fusco, L. D'Accolti, F. lannone, A. Nacci, *J. Organomet. Chem.* **2022**, *958*, 122193.

(5) W. Ying, J. W. Herndon, *Eur. J. Org. Chem.* **2013**, *2013*, 3112–3122.

(6) D. Stærk, A. K. Lykkeberg, J. Christensen, B. A. Budnik, F. Abe, J. W. Jaroszewski, *J. Nat. Pr od.* **2002**, *65*, 1299–1302.

(7) M. J. Niphakis, G. I. Georg, *Org. Lett.* **2011**, *13*, 196–199.

(8) F. Abe, Y. Iwase, T. Yamauchi, K. Honda, N. Hayashi, *Phytochemistry*, **1995**, *39*, 695–699.

(9) H. Suzuki, S. Aoyagi, C. Kibayashi, *J. Org. Chem.* **1995**, *60*, 6114-6122.

(10) a) B. J. Delley, *Chem. Phys.* **1990**, *92*, 508–517. b) B. J. Delley, *Chem. Phys.* **2000**, *113*, 775 6–7764.

(11) J. P. Perdew, K. Burke K. M. Ernzerhof, *Phys. Rev. Lett.* **1996**, *77*, 3865–3868.

5 Copies of NMR data

^{1}H NMR (400 MHz, $CDCl_3$) of **1**

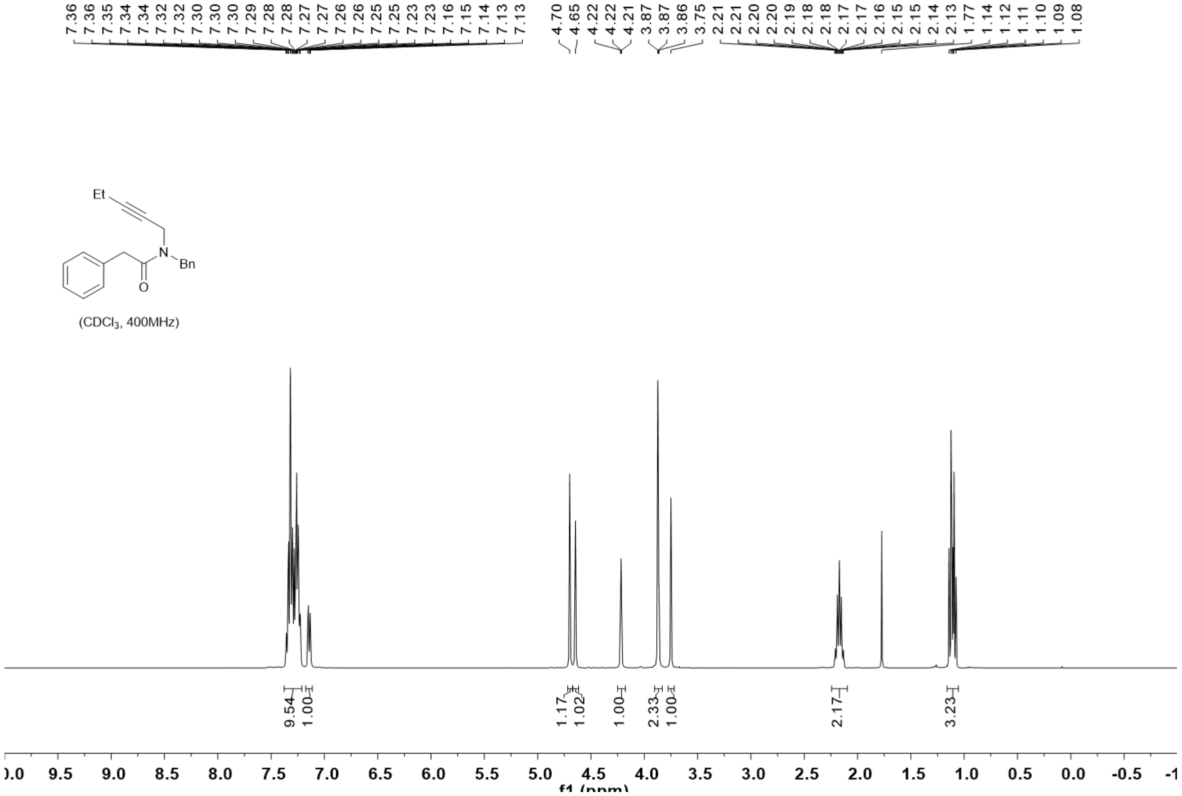

^{13}C NMR (100 MHz, $CDCl_3$) of **1**

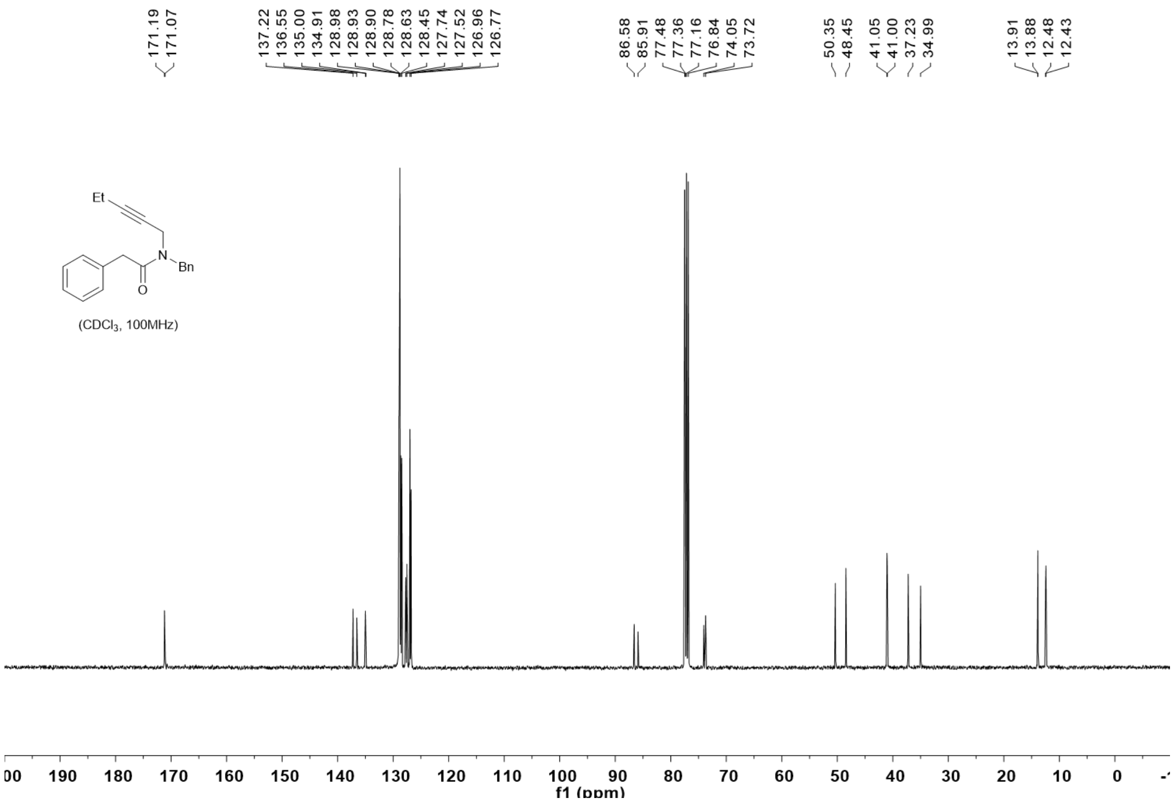

^{1}H NMR (400 MHz, $CDCl_3$) of **2**

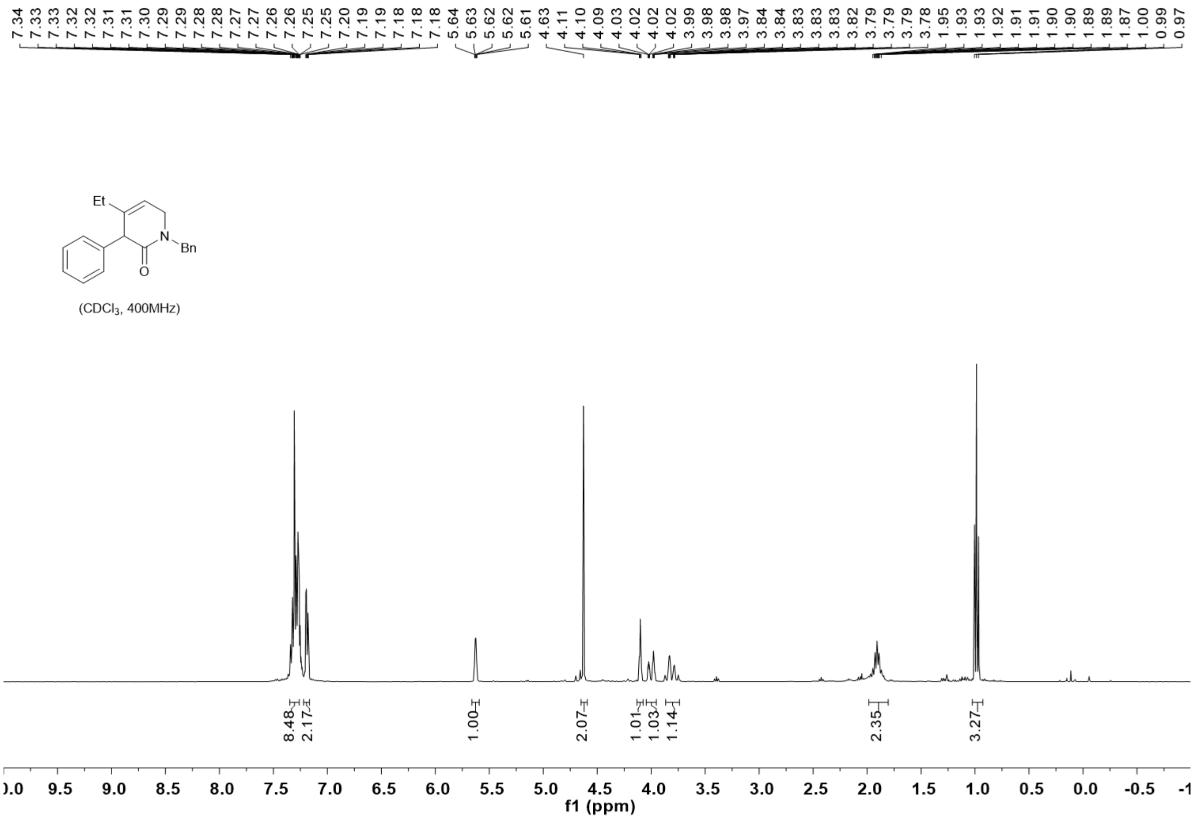

^{13}C NMR (100 MHz, $CDCl_3$) of **2**

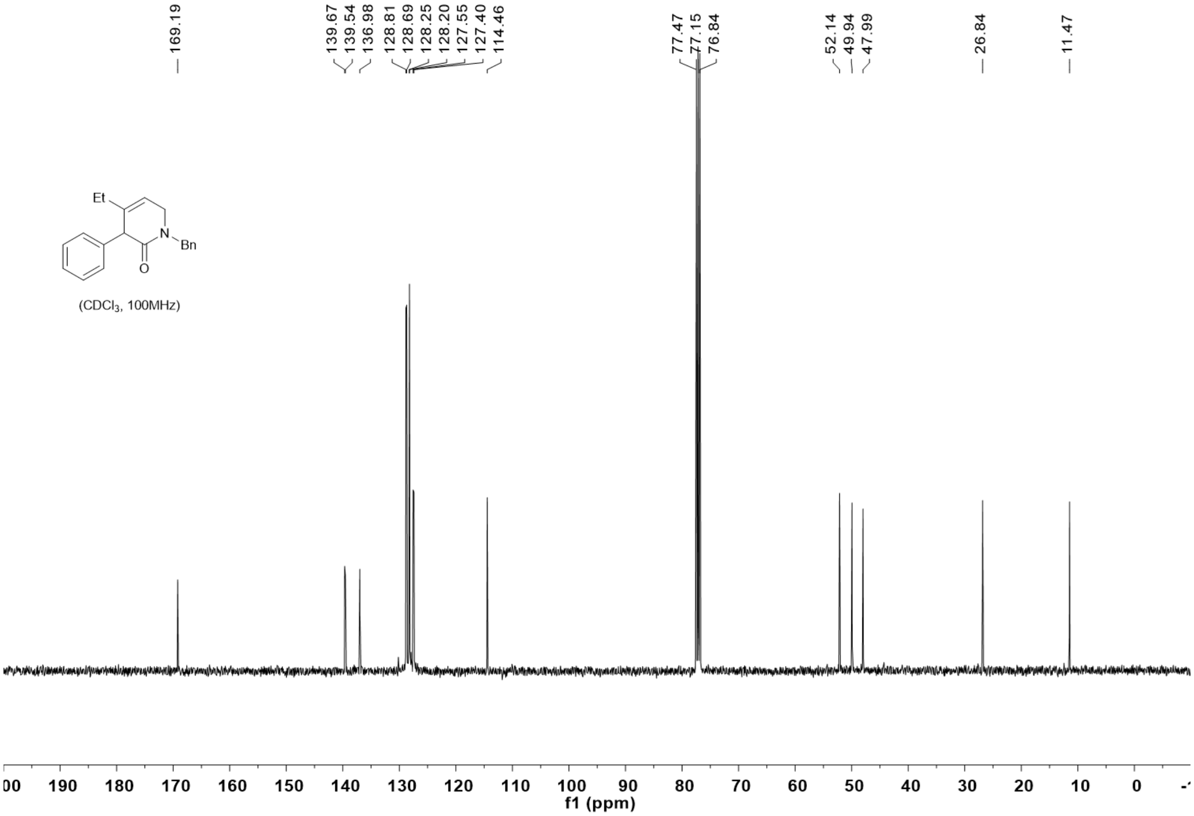

^{1}H NMR (400 MHz, $CDCl_3$) of **5**

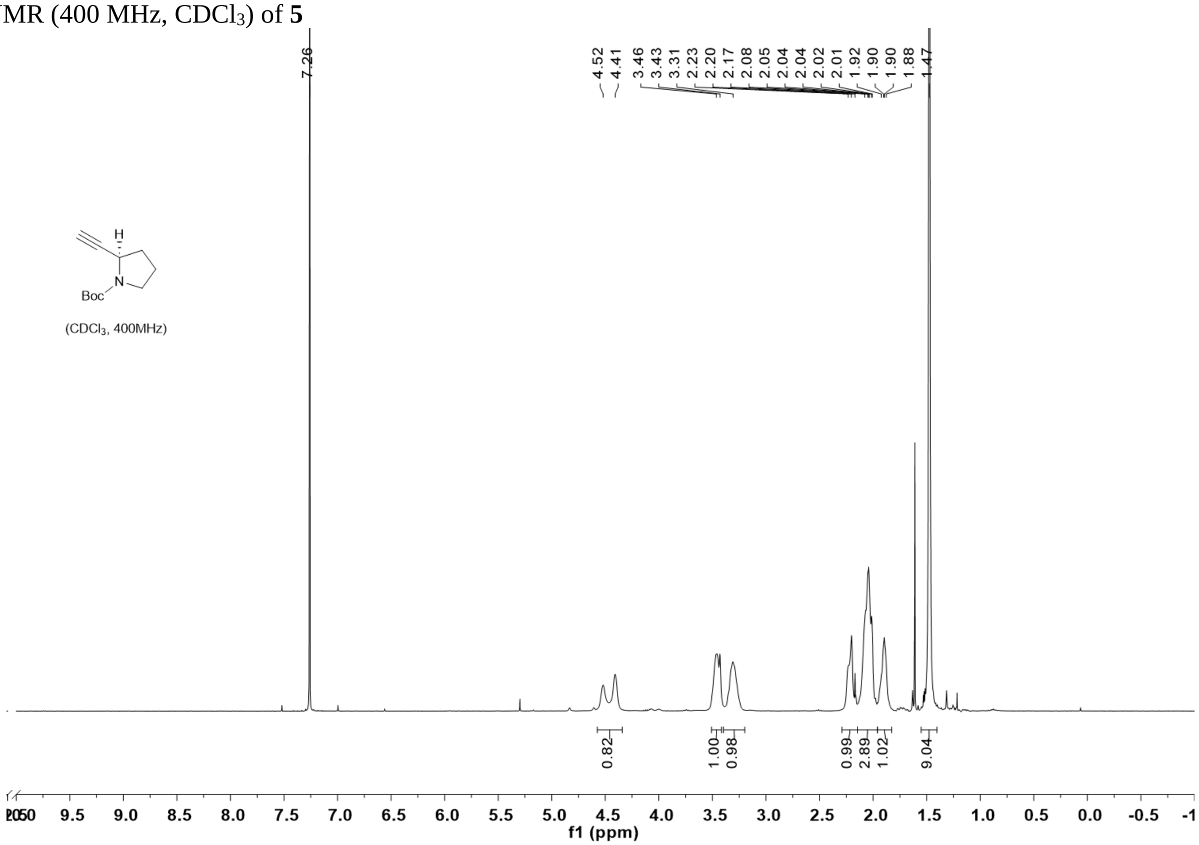

^{1}H NMR (400 MHz, $CDCl_3$) of **6a**

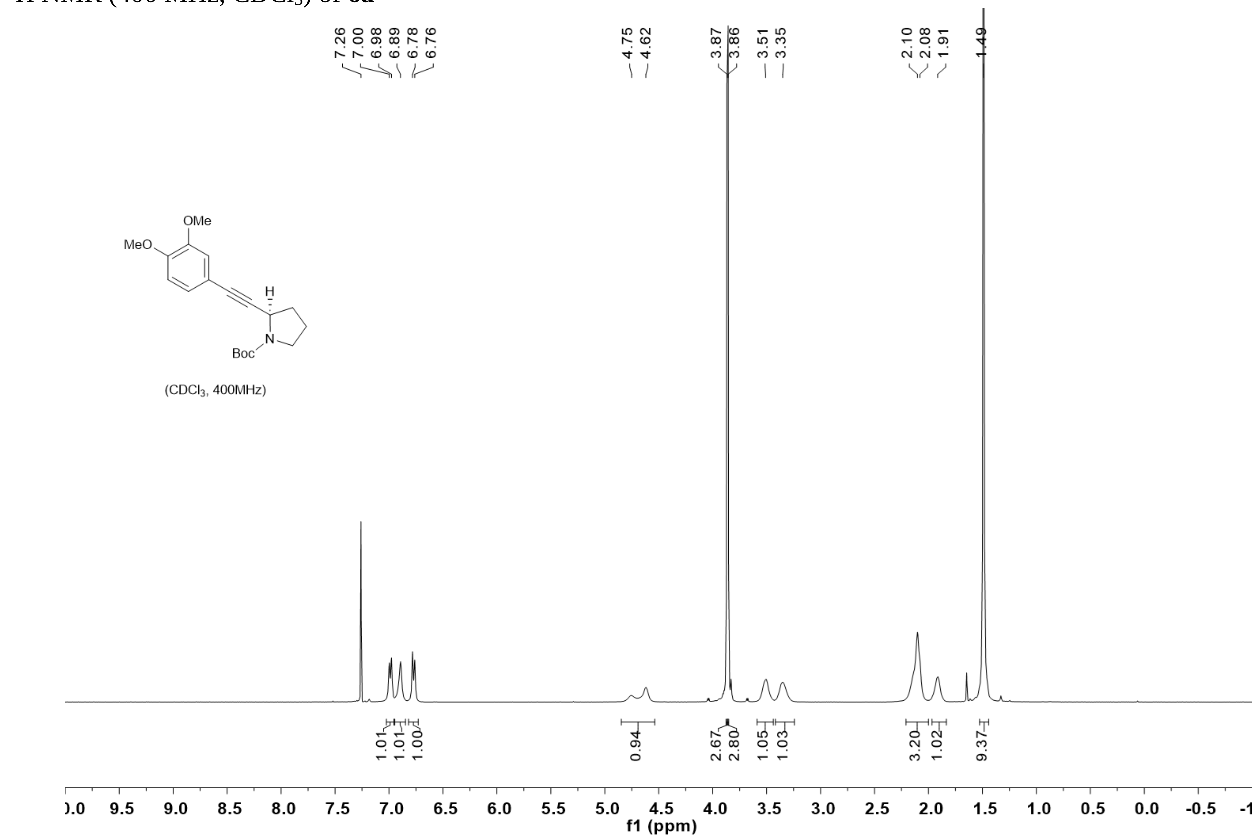

^{13}C NMR (100 MHz, $CDCl_3$) of **6a**

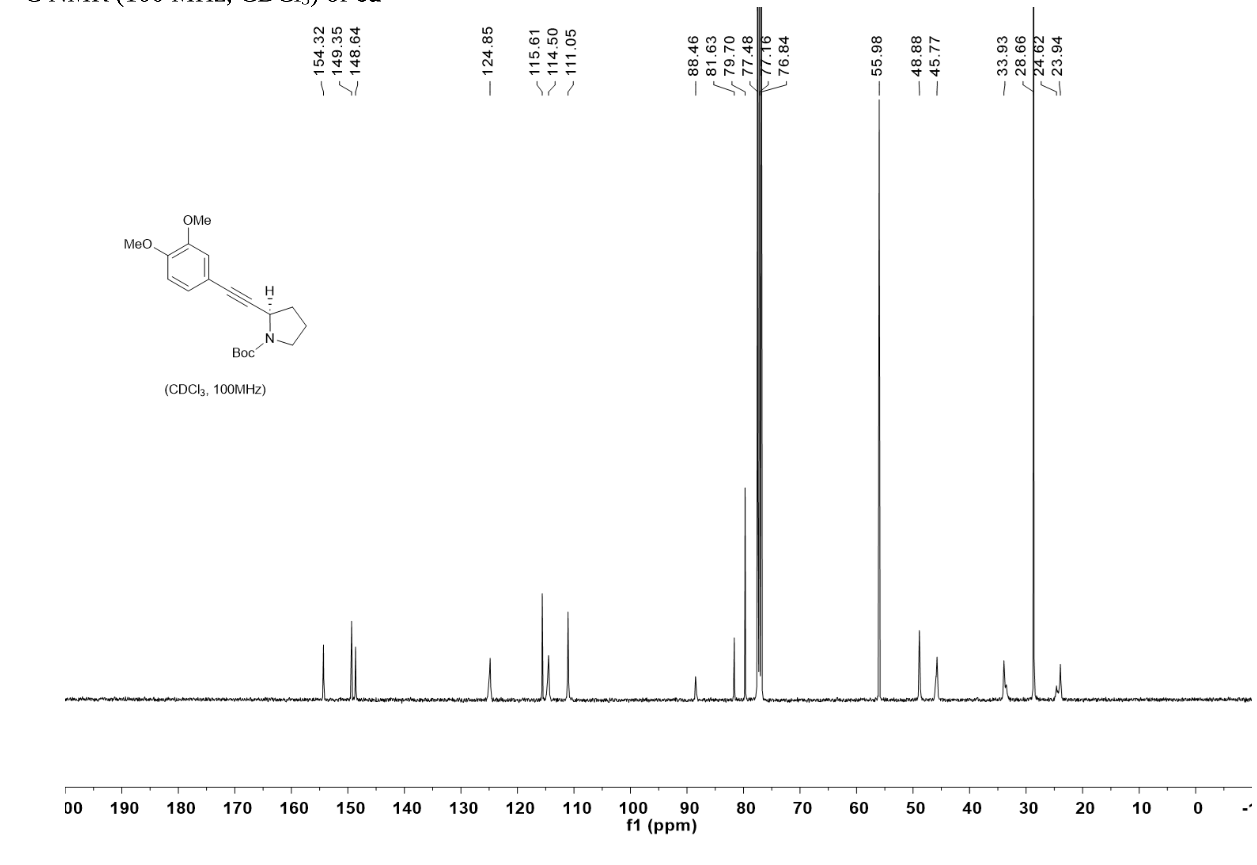

^{1}H NMR (400 MHz, $CDCl_3$) of **8a**

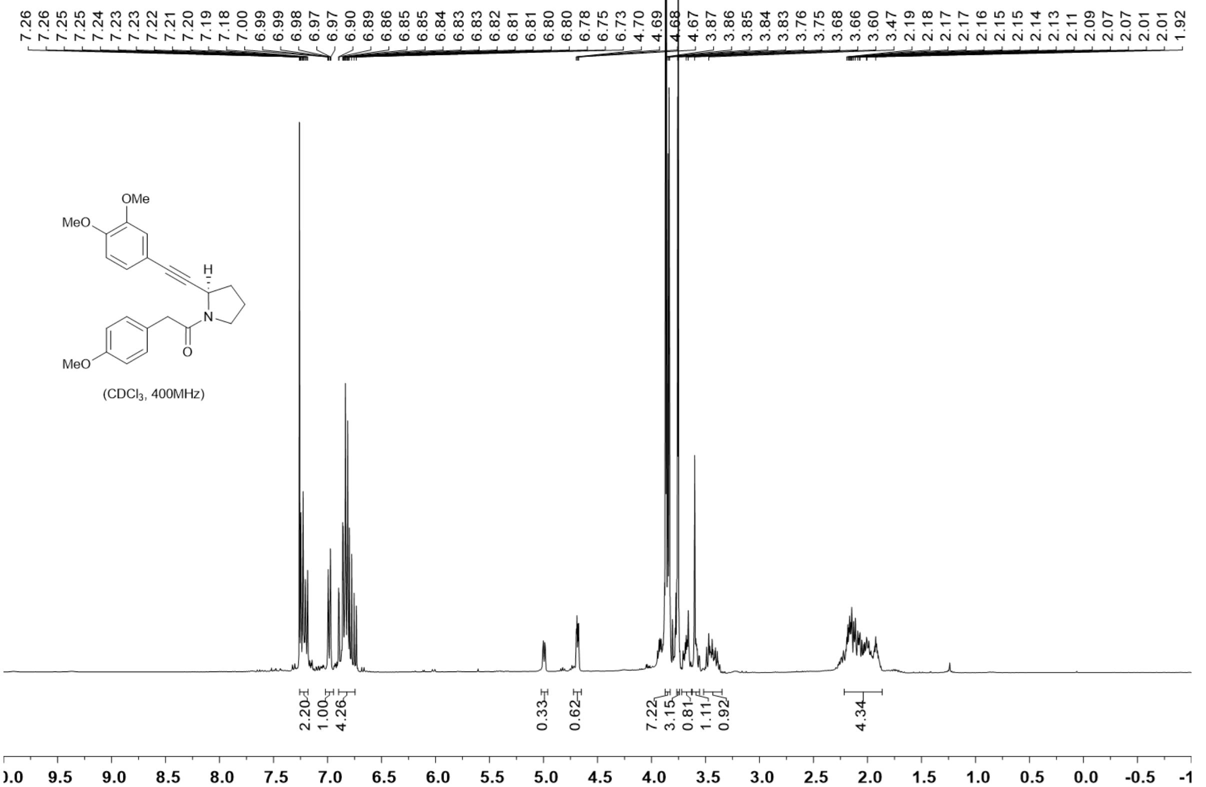

^{13}C NMR (100 MHz, $CDCl_3$) of **8a**

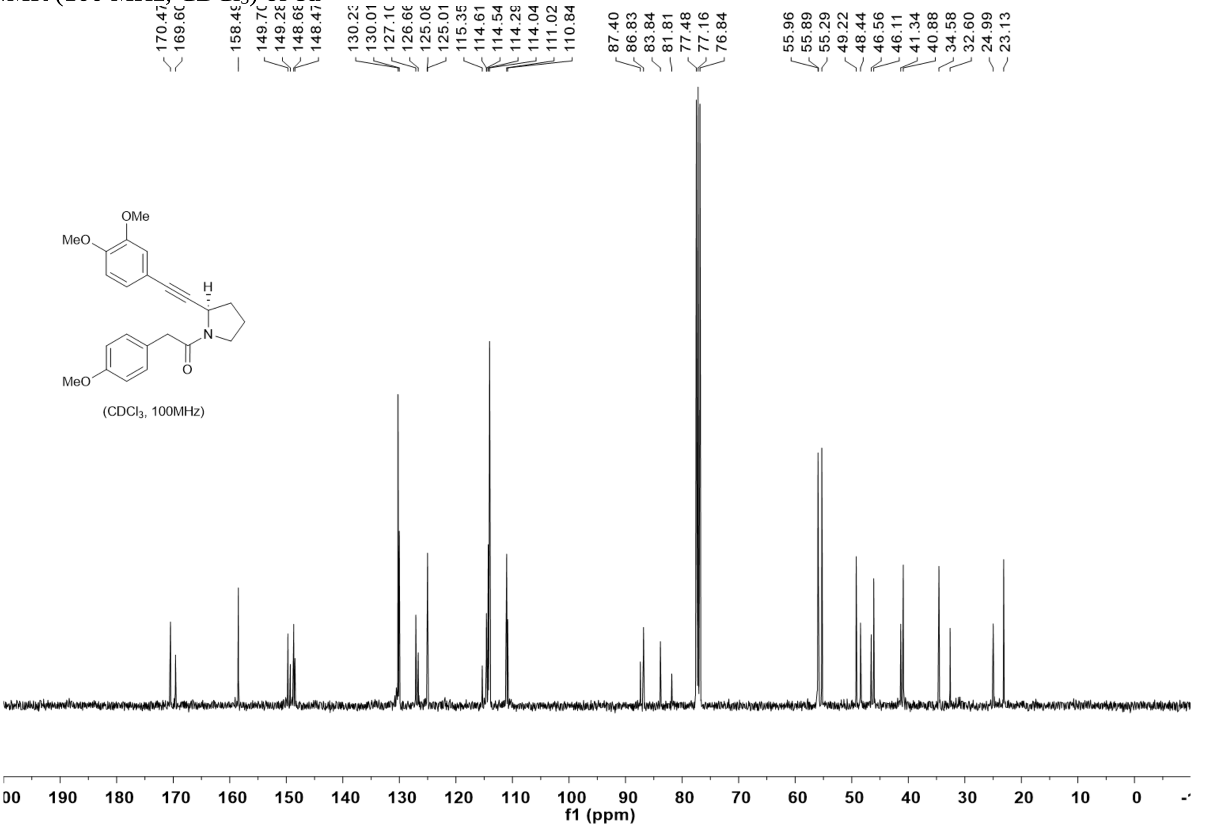

^{1}H NMR (400 MHz, $CDCl_3$) of **9a**

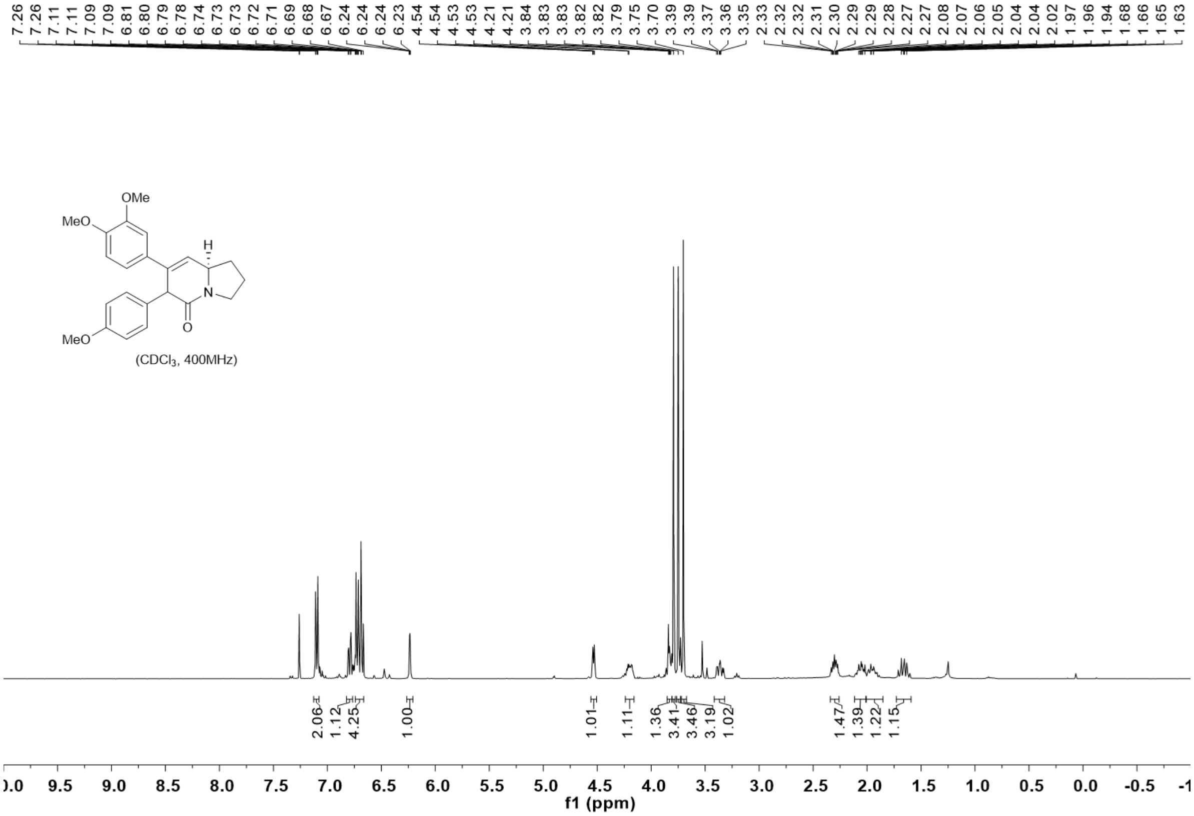

^{13}C NMR (100 MHz, $CDCl_3$) of **9a**

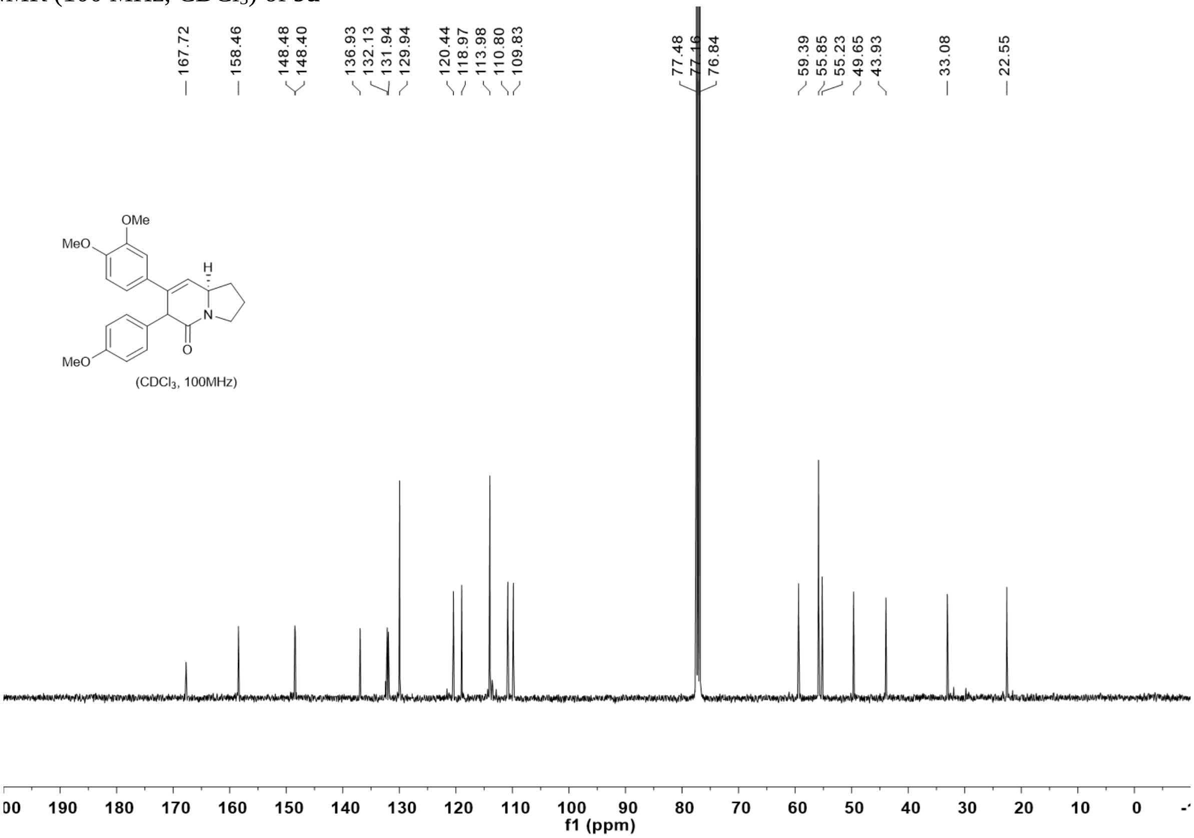

^{1}H NMR (400 MHz, $CDCl_3$) of **10a**

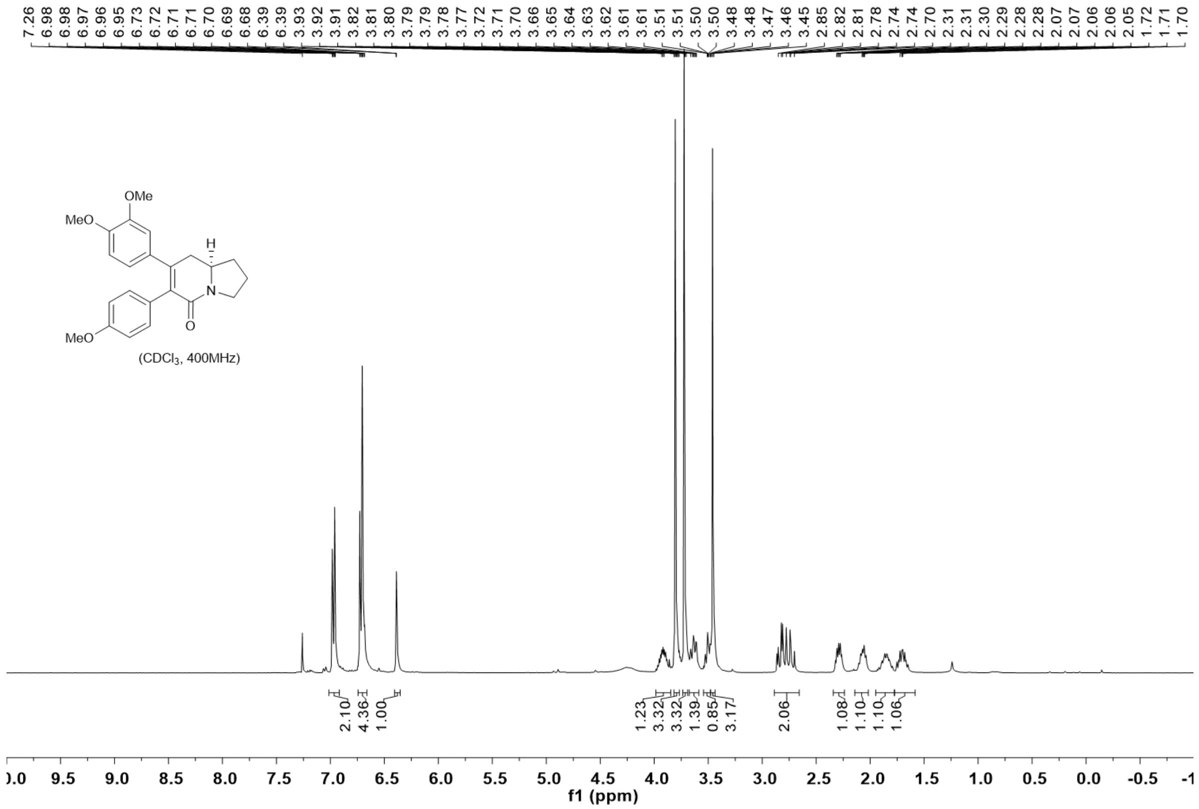

^{13}C NMR (100 MHz, $CDCl_3$) of **10a**

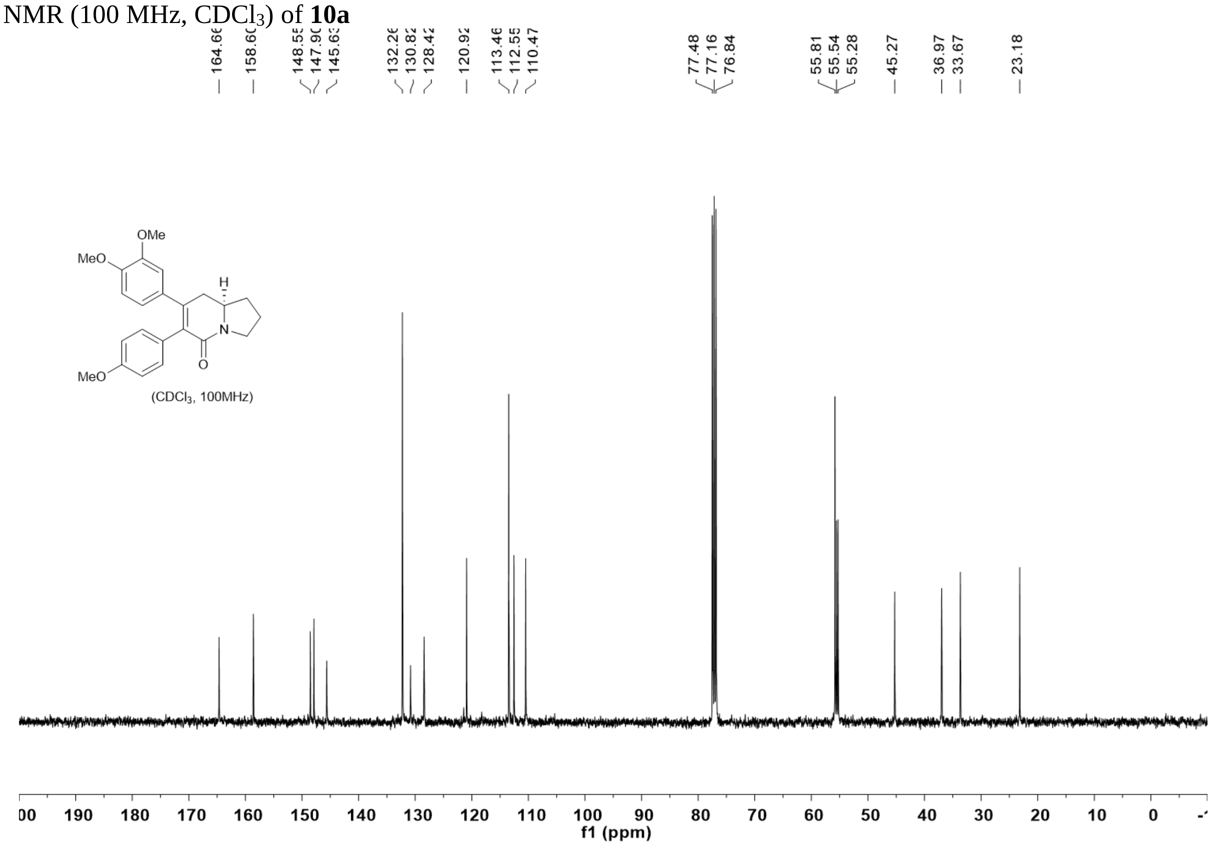

^{1}H NMR (400 MHz, $CDCl_3$) of **11a**

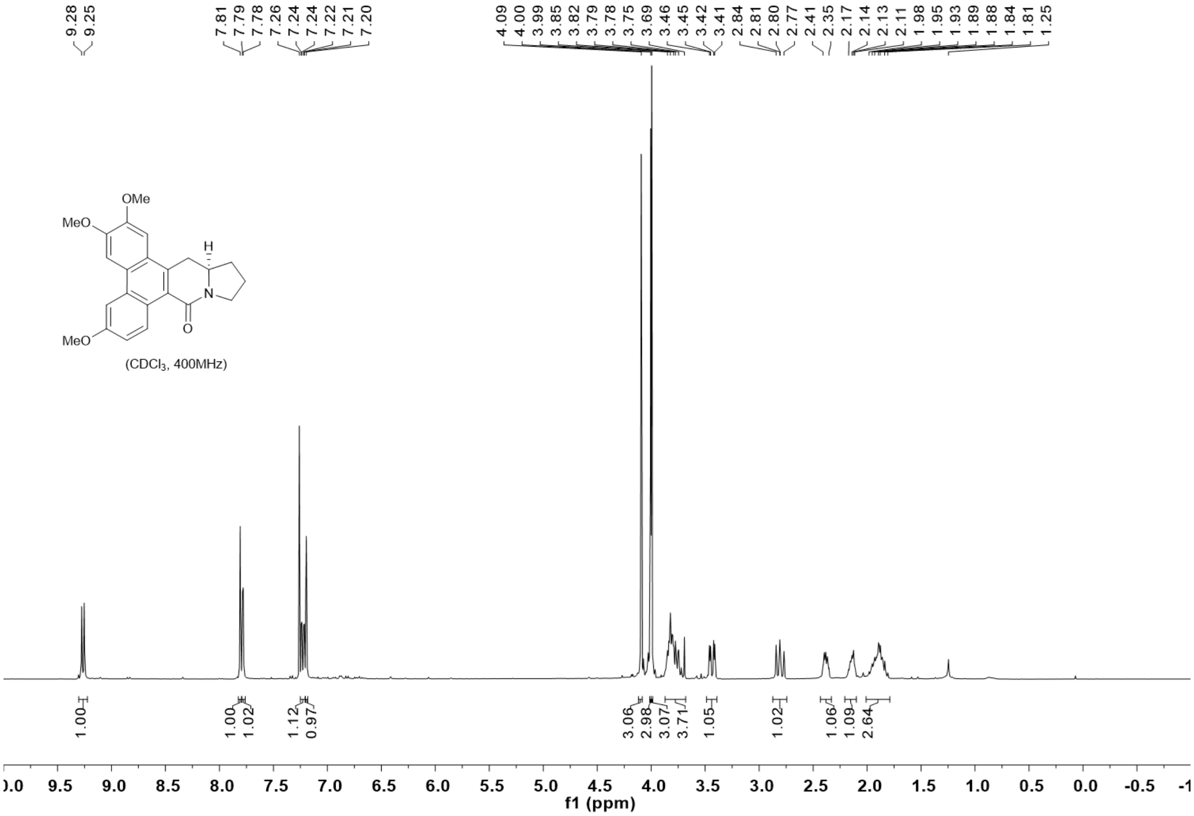

^{13}C NMR (100 MHz, $CDCl_3$) of **11a**

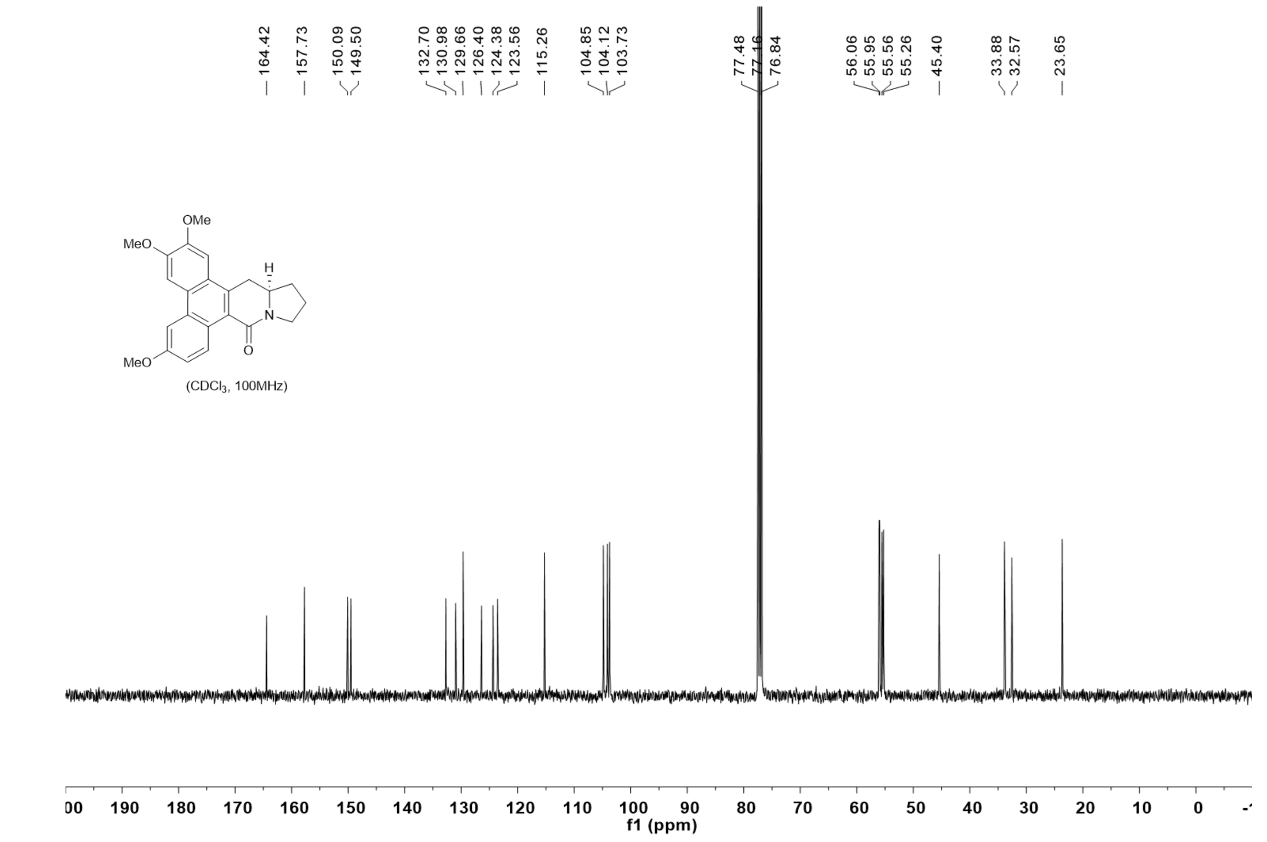

^{1}H NMR (400 MHz, $CDCl_3$) of **3a**

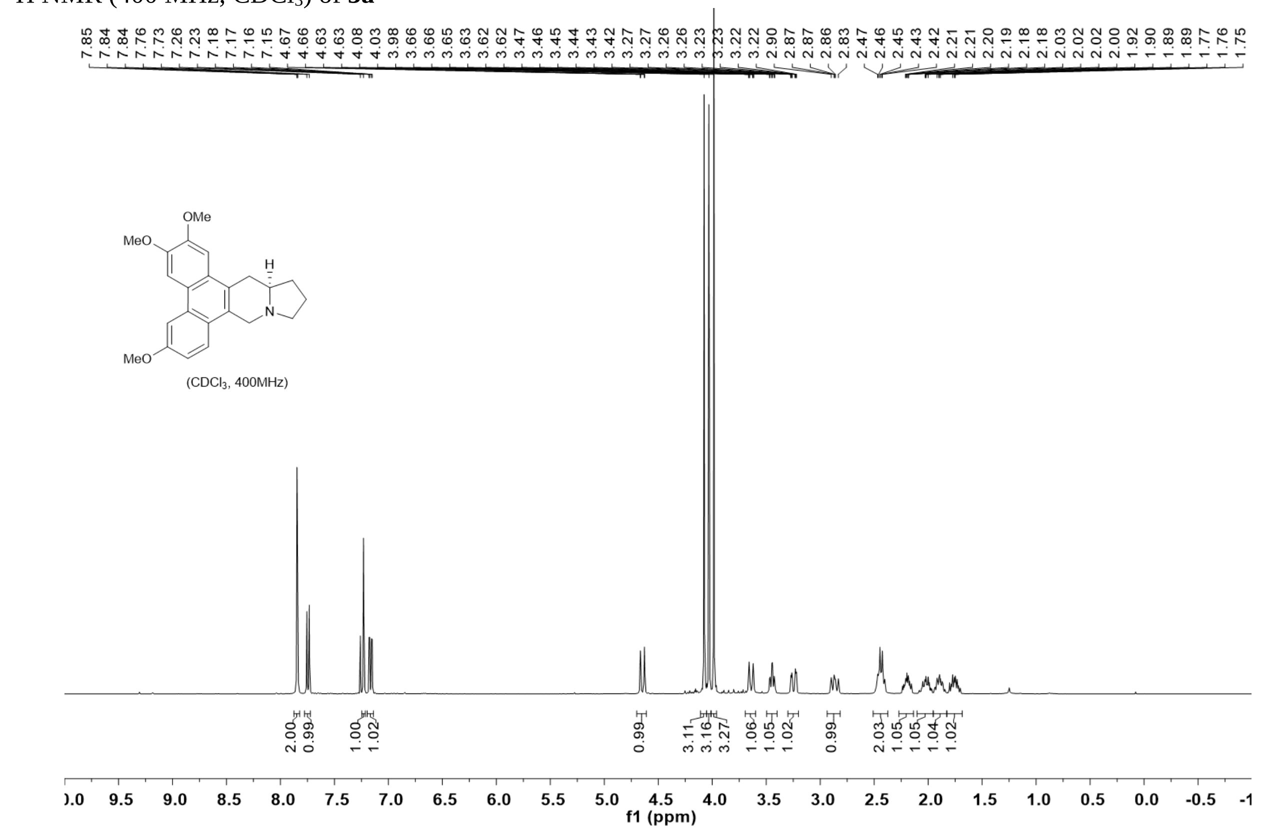

^{13}C NMR (100 MHz, $CDCl_3$) of **3a**

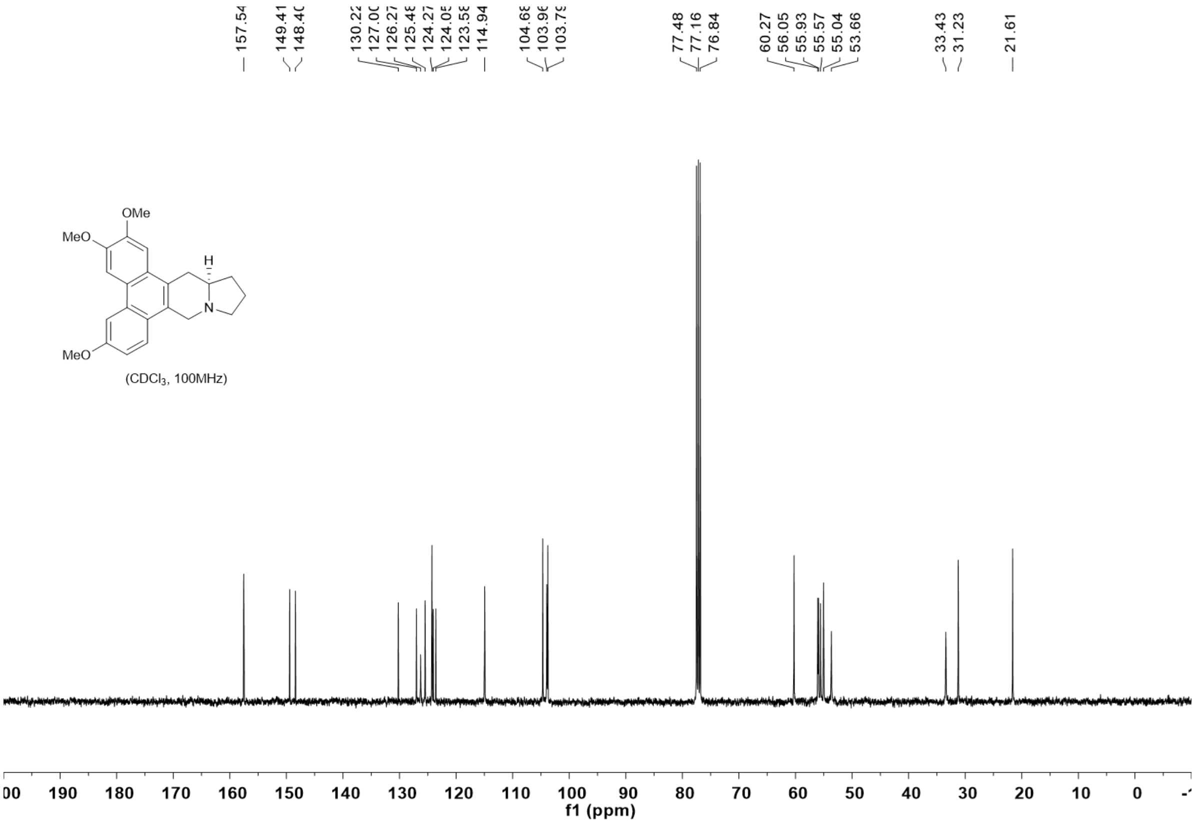

^{1}H NMR (400 MHz, $CDCl_3$) of **8b**

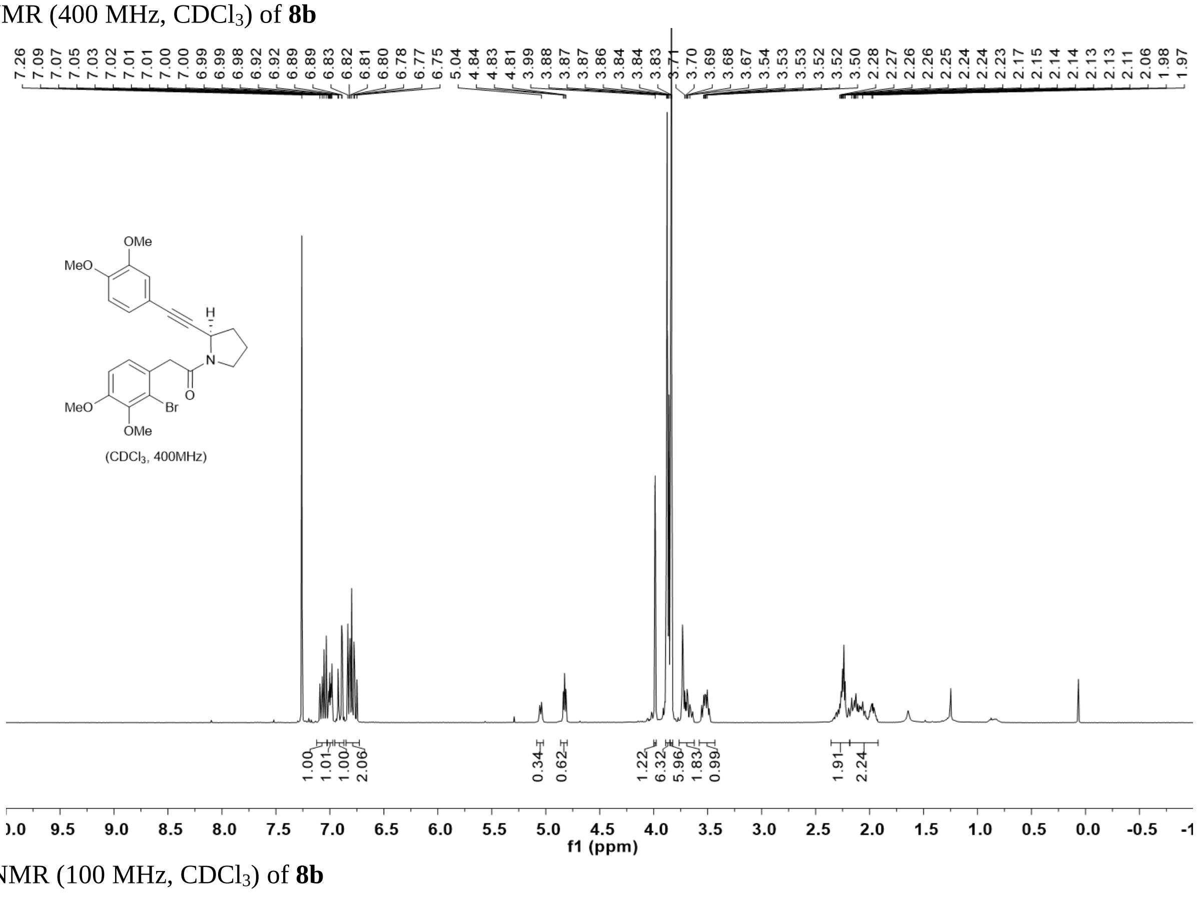

^{13}C NMR (100 MHz, $CDCl_3$) of **8b**

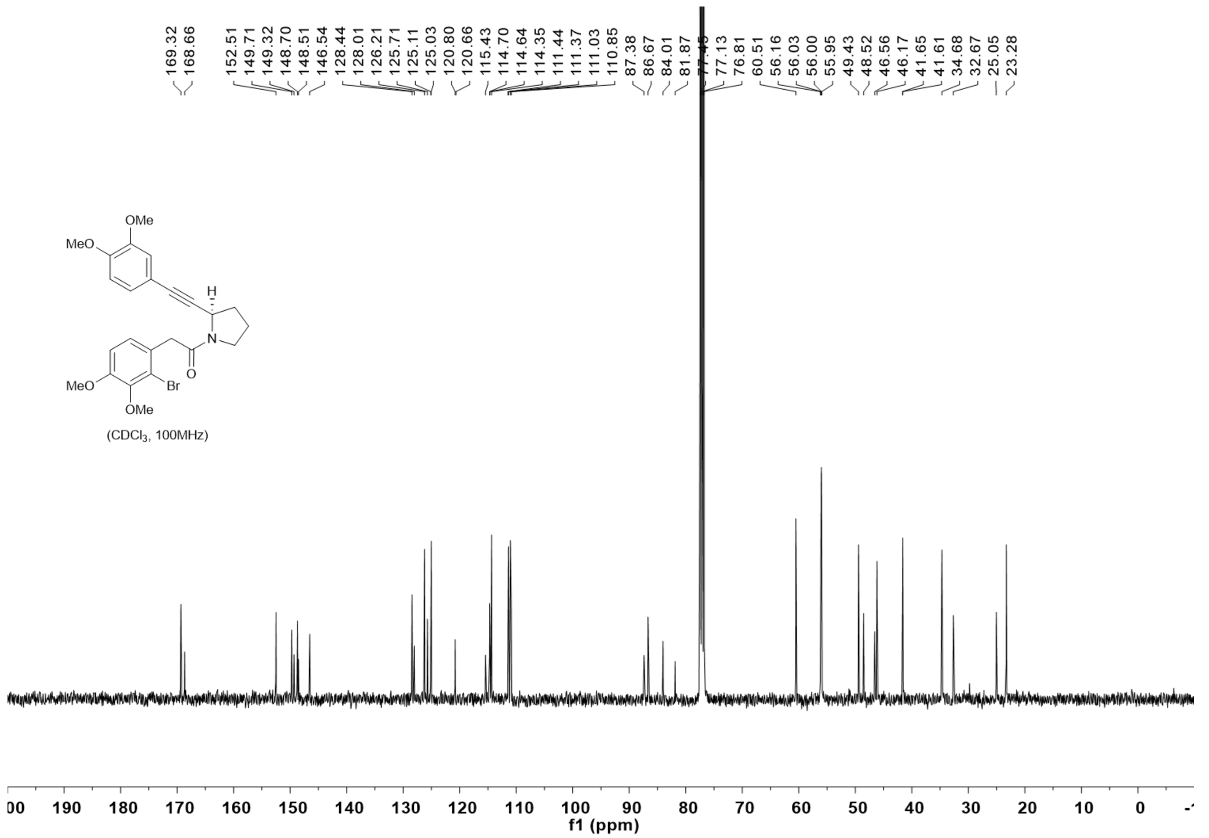

^{1}H NMR (400 MHz, $CDCl_3$) of **10b**

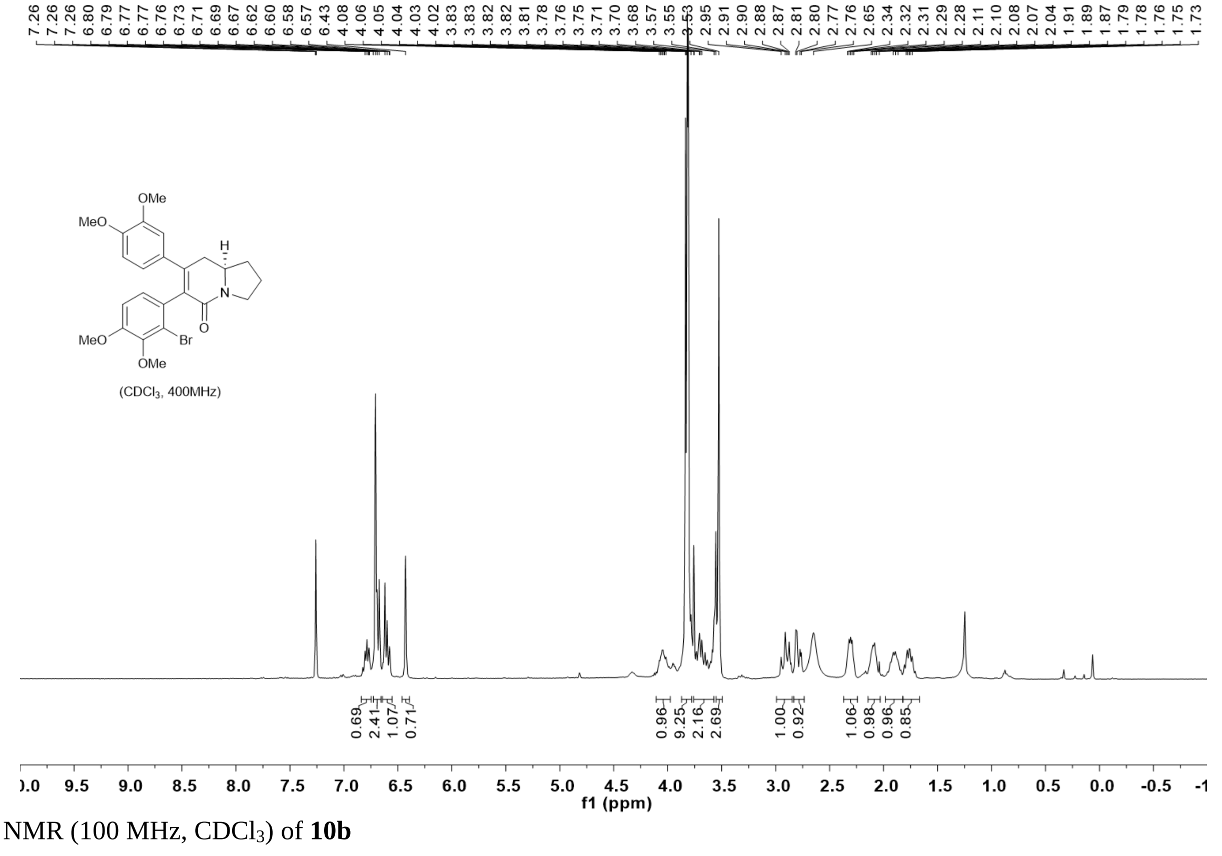

^{13}C NMR (100 MHz, $CDCl_3$) of **10b**

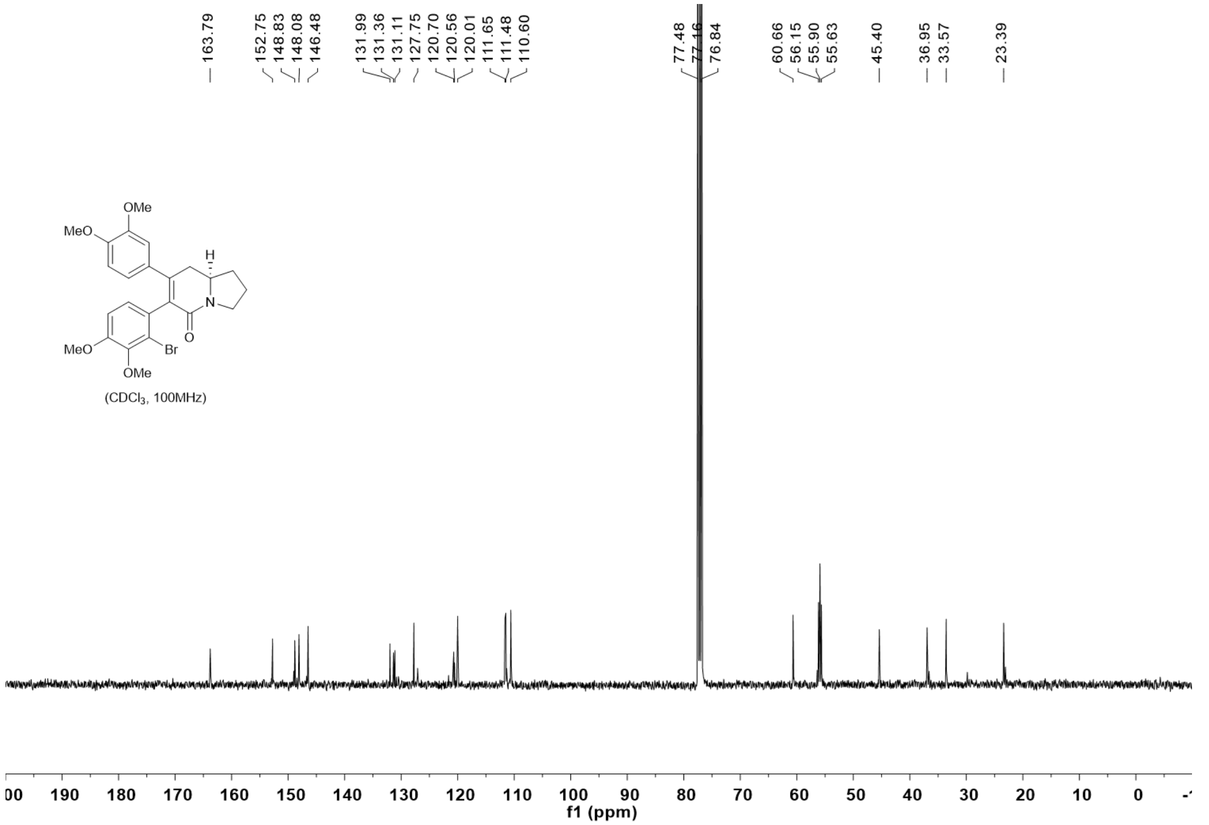

^{1}H NMR (400 MHz, $CDCl_3$) of **11b**

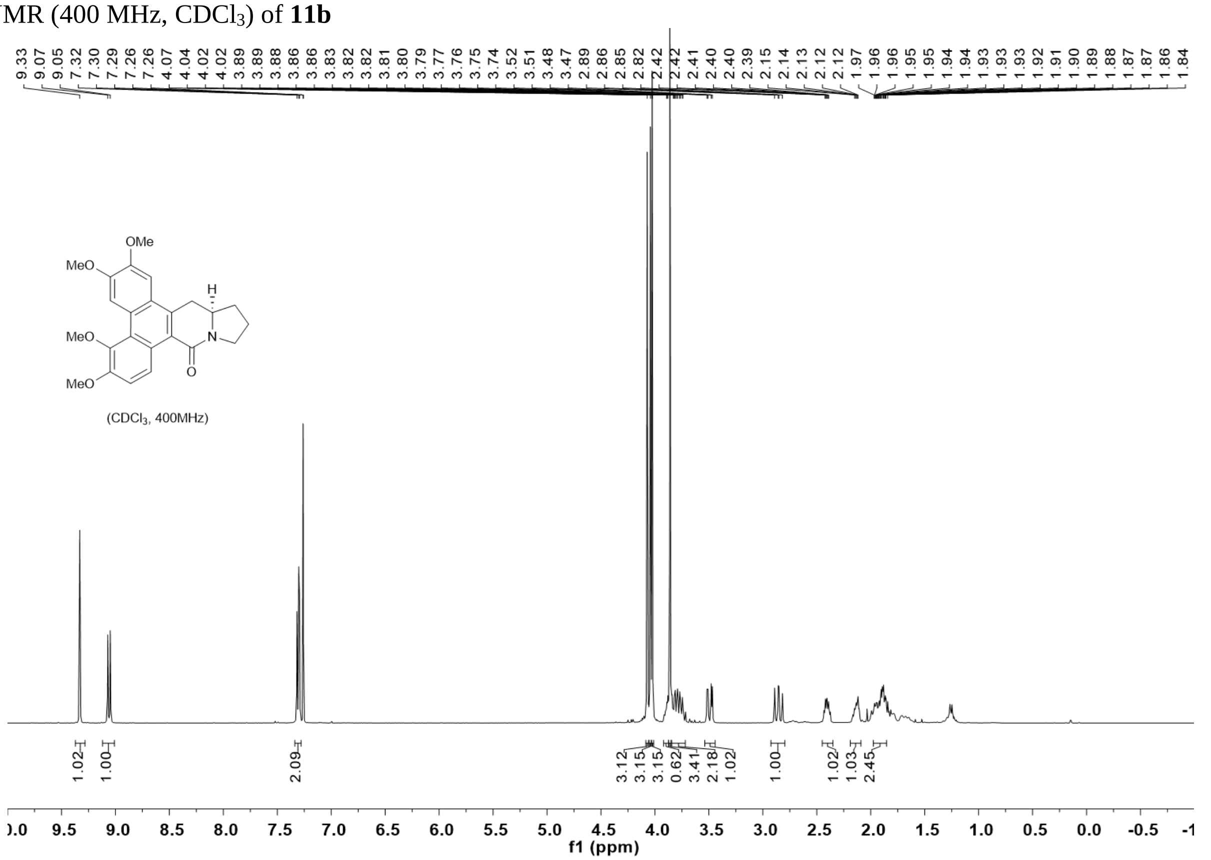

^{13}C NMR (100 MHz, $CDCl_3$) of **11b**

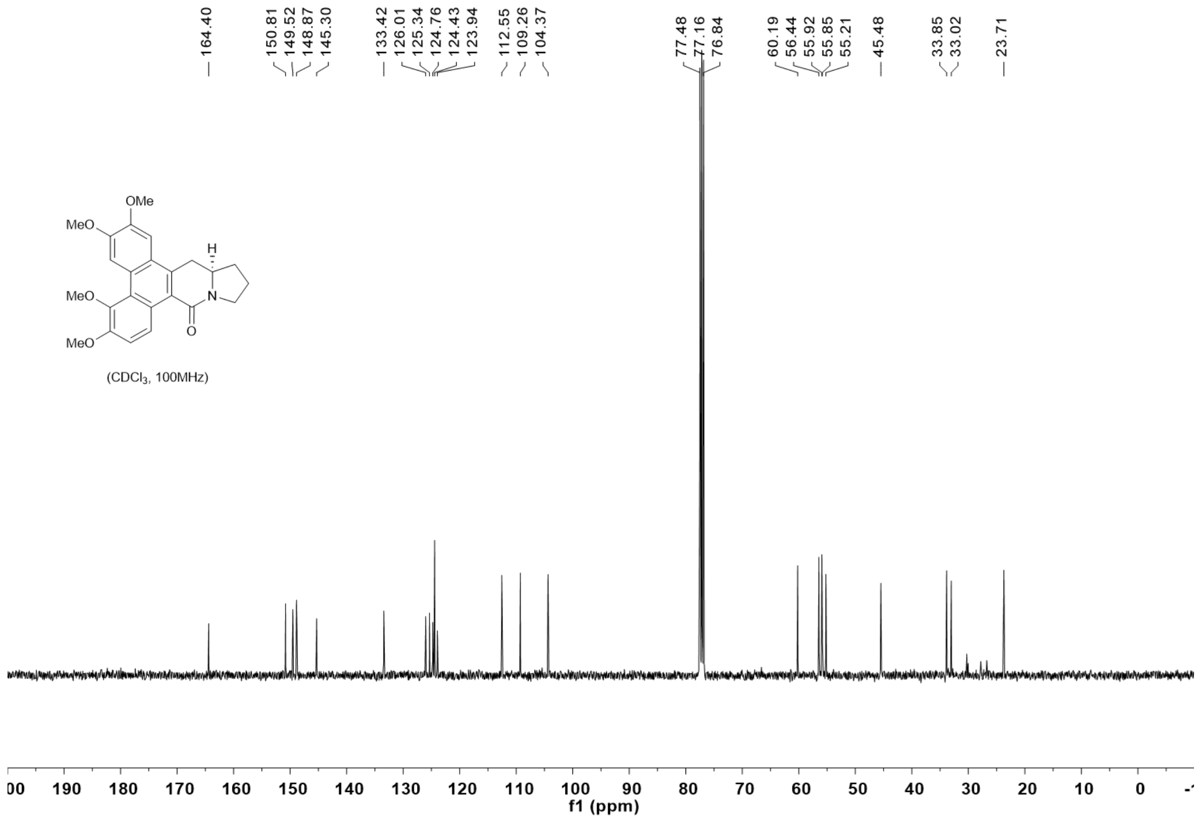

^{1}H NMR (400 MHz, $CDCl_3$) of **3b**

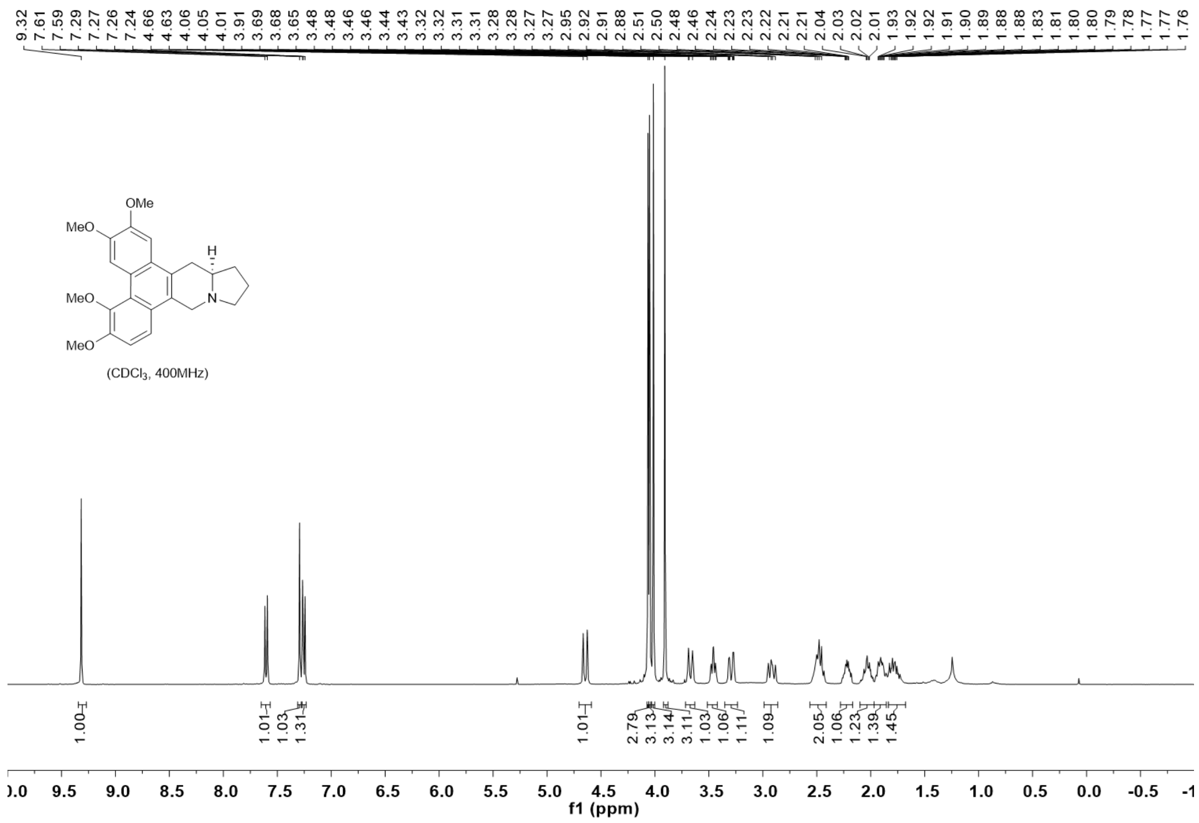

^{13}C NMR (100 MHz, $CDCl_3$) of **3b**

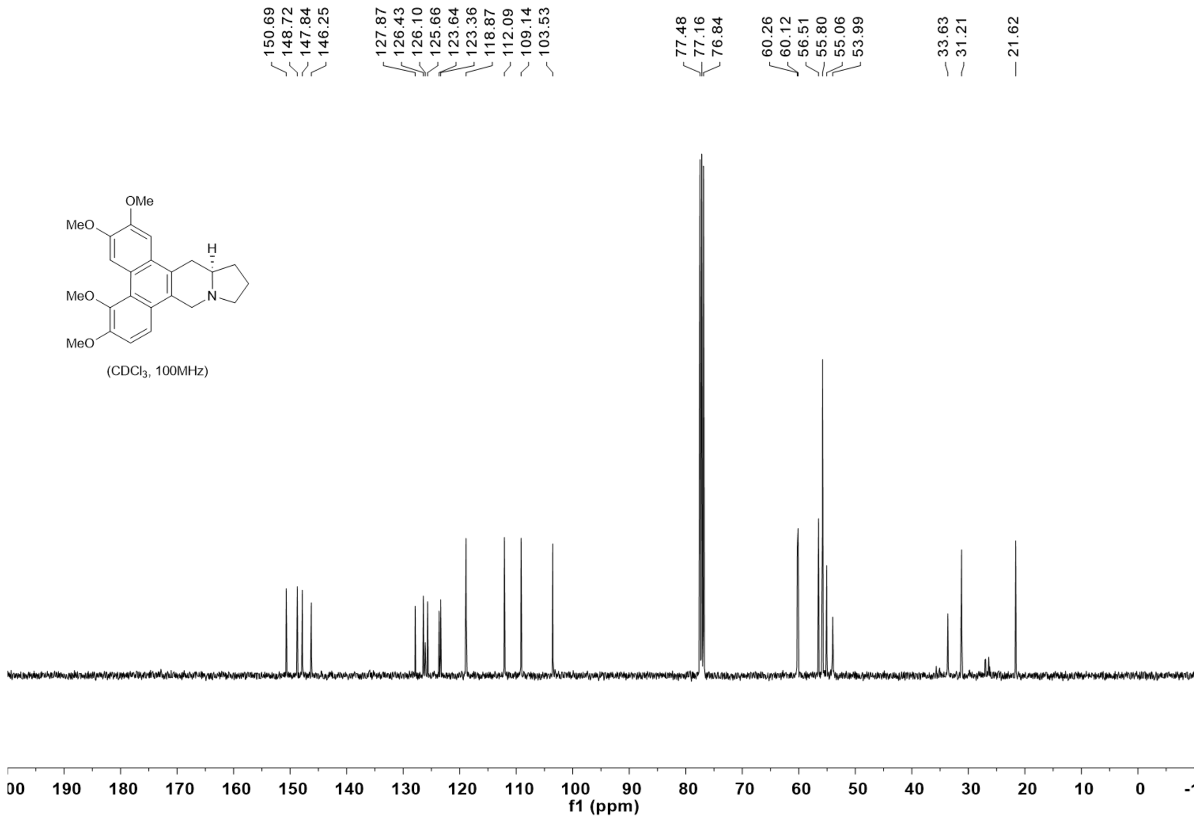

^{1}H NMR (400 MHz, $CDCl_3$) of **6c**

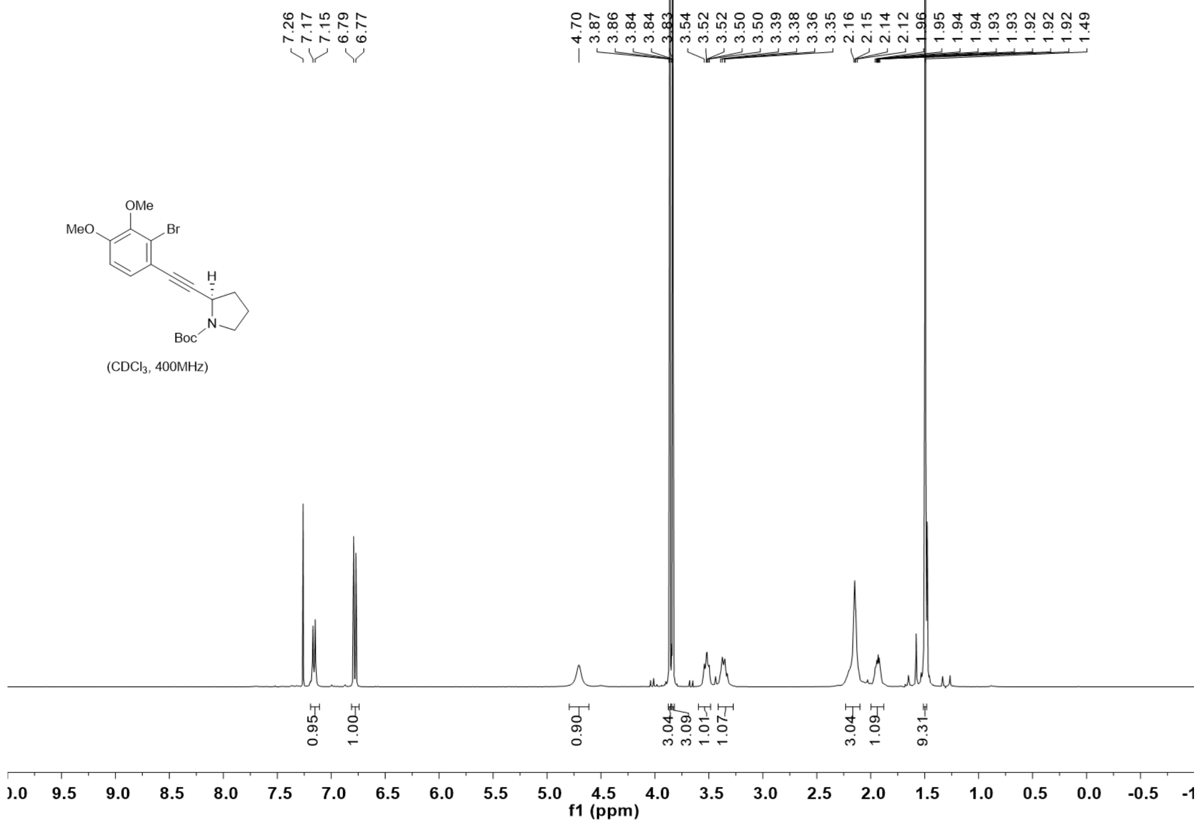

^{13}C NMR (100 MHz, $CDCl_3$) of **6c**

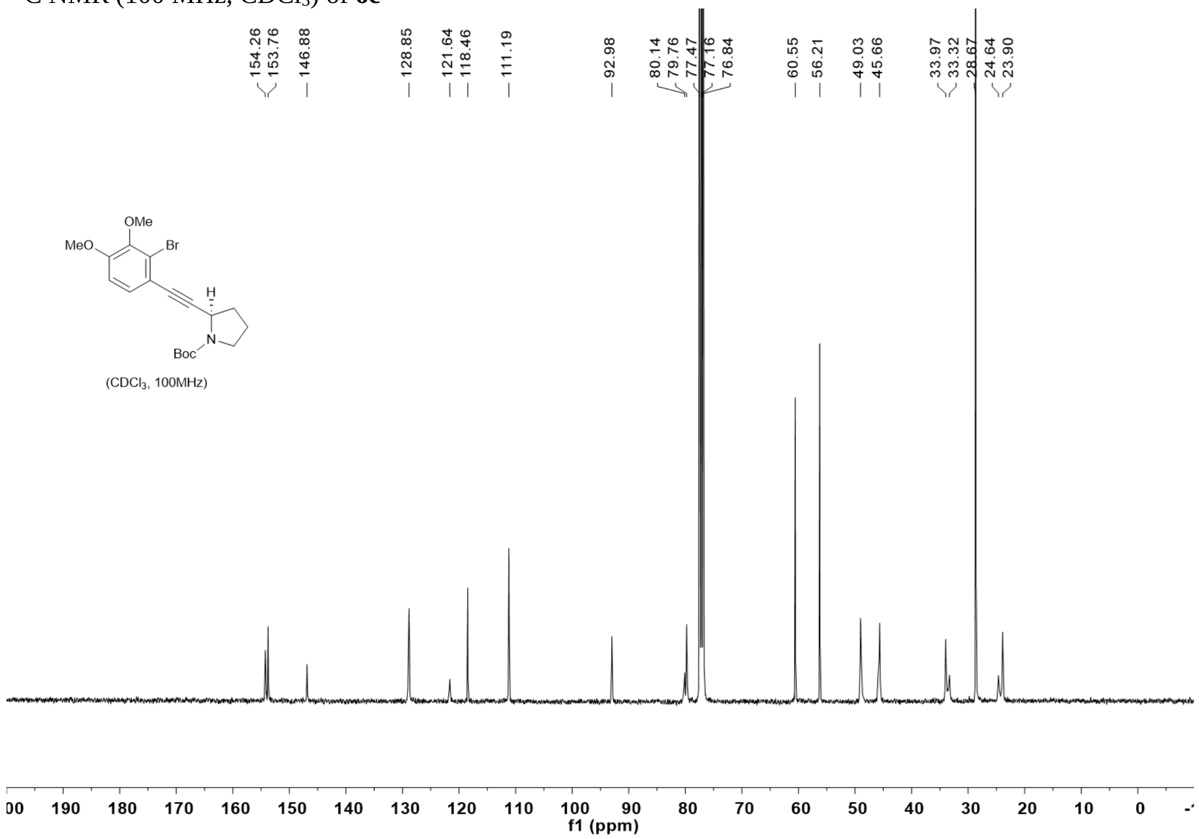

^{1}H NMR (400 MHz, $CDCl_3$) of **8c**

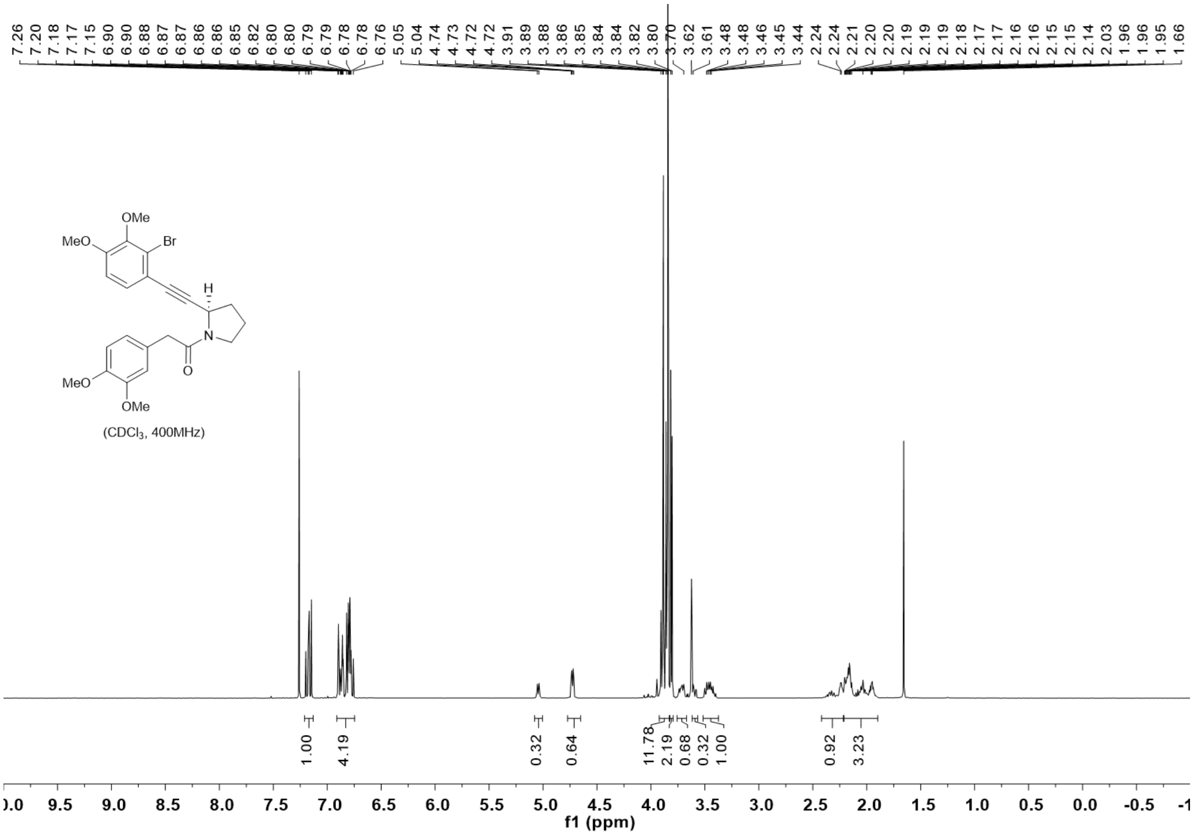

^{13}C NMR (100 MHz, $CDCl_3$) of **8c**

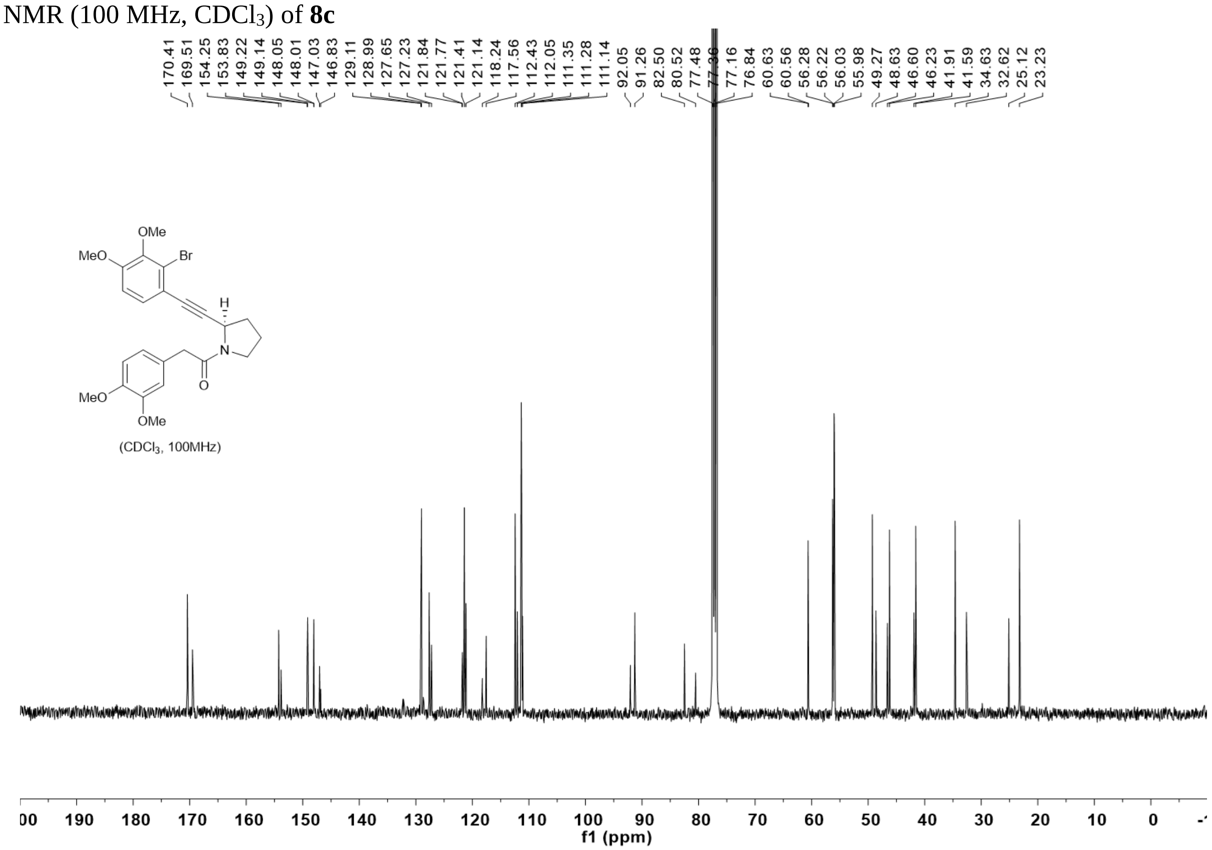

^{1}H NMR (400 MHz, $CDCl_3$) of **9c**

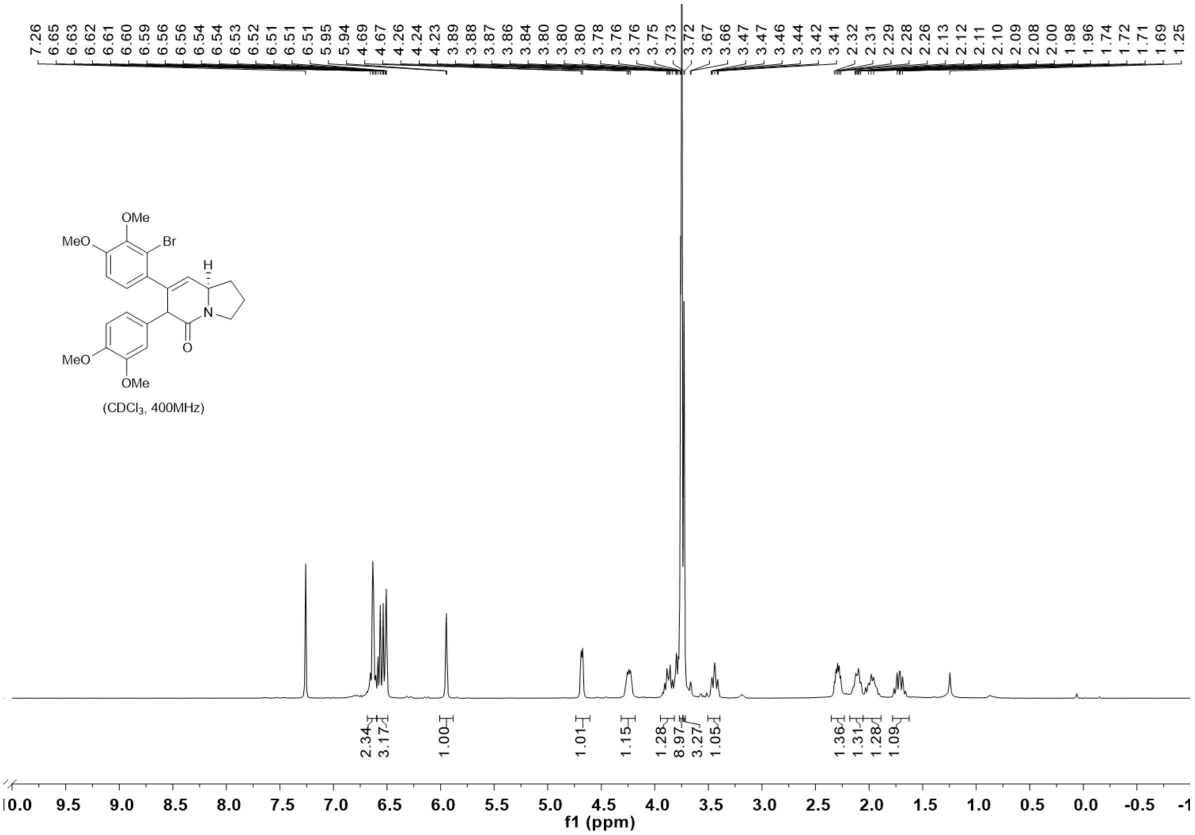

^{13}C NMR (100 MHz, $CDCl_3$) of **9c**

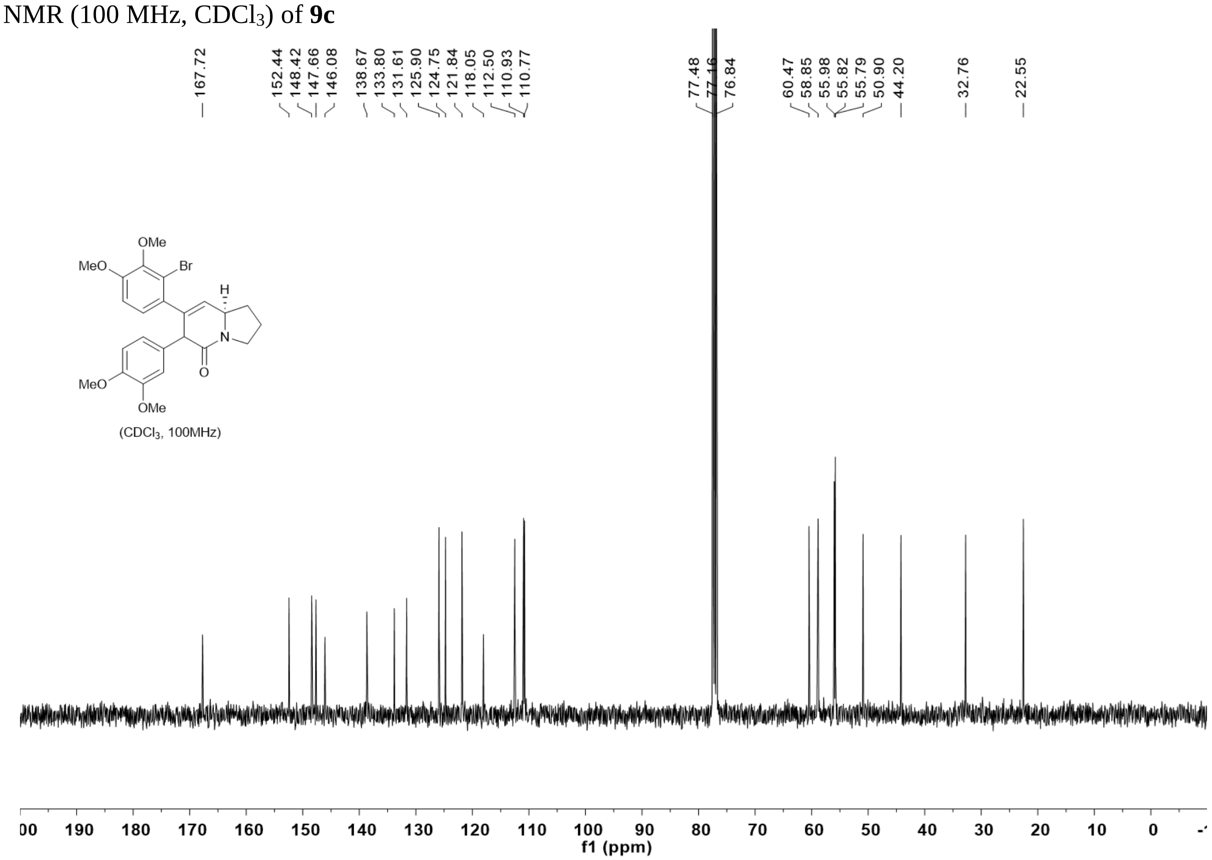

^{1}H NMR (400 MHz, $CDCl_3$) of **10c**

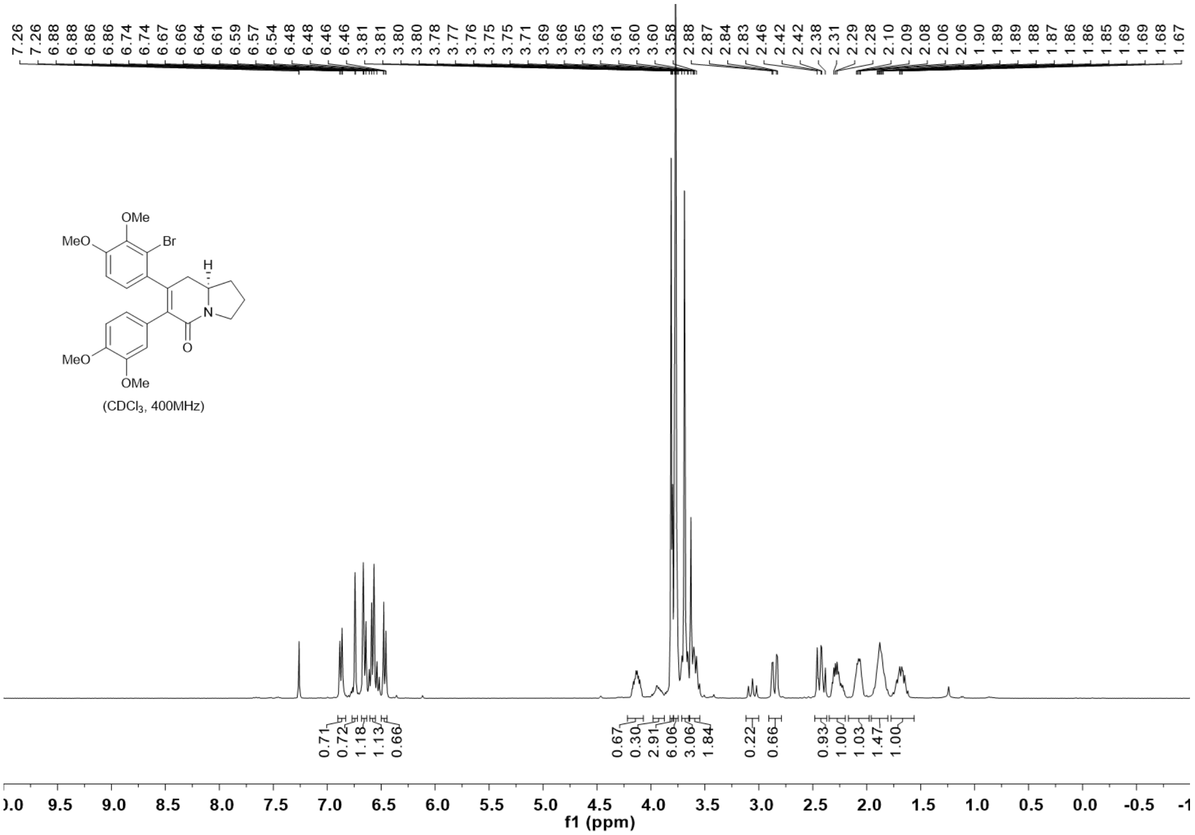

^{13}C NMR (100 MHz, $CDCl_3$) of **10c**

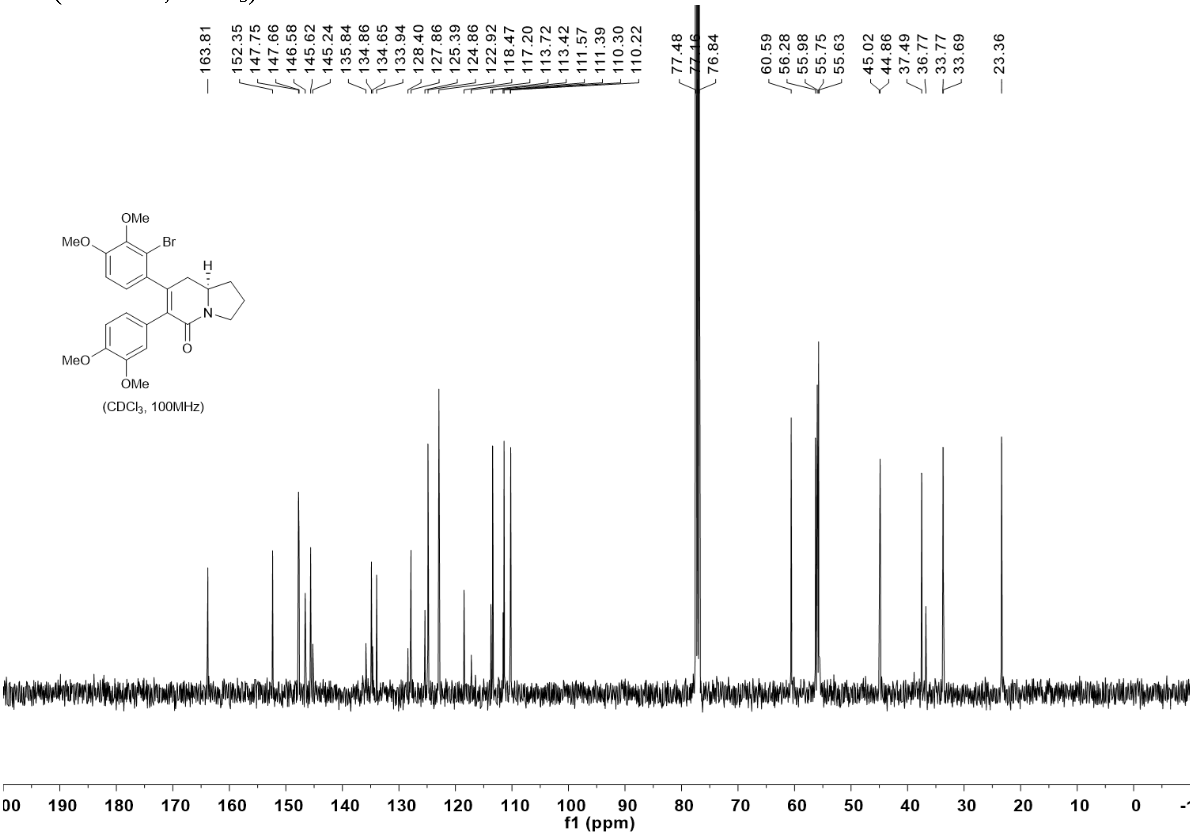

^{1}H NMR (400 MHz, $CDCl_3$) of **11c**

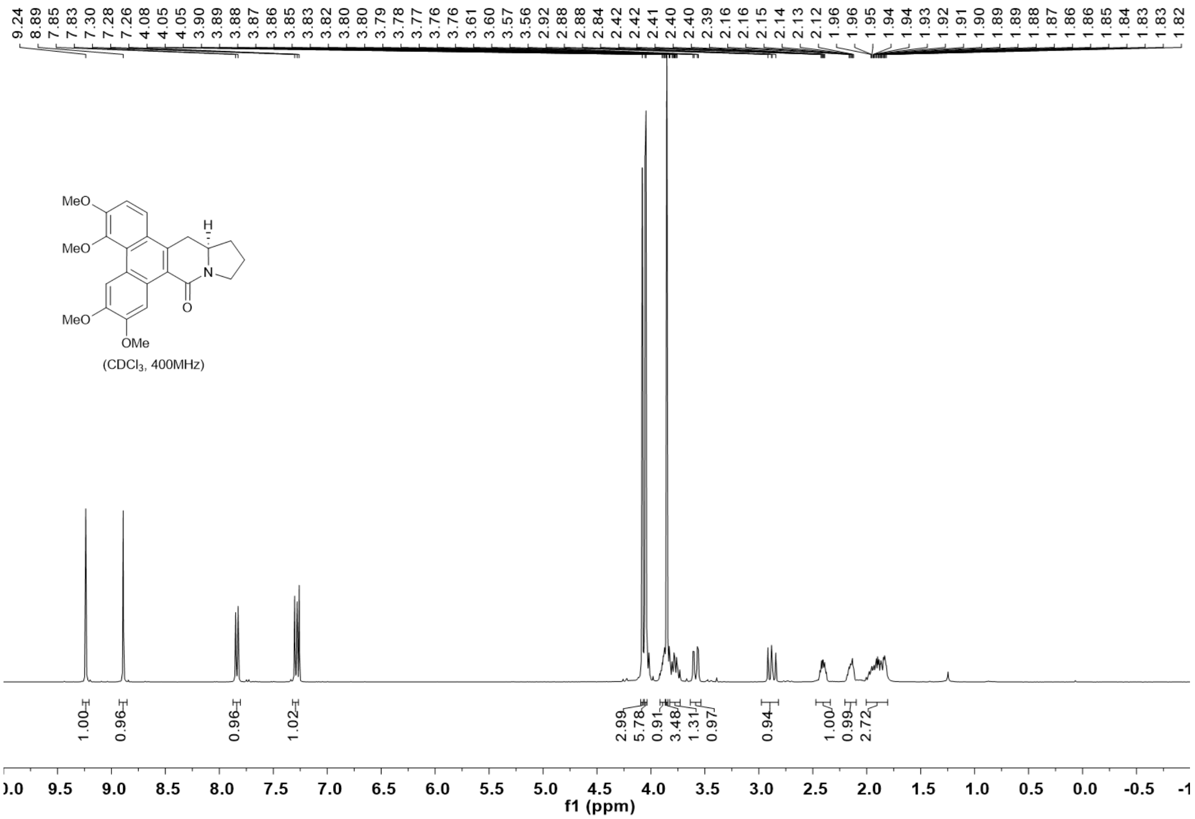

^{13}C NMR (100 MHz, $CDCl_3$) of **11c**

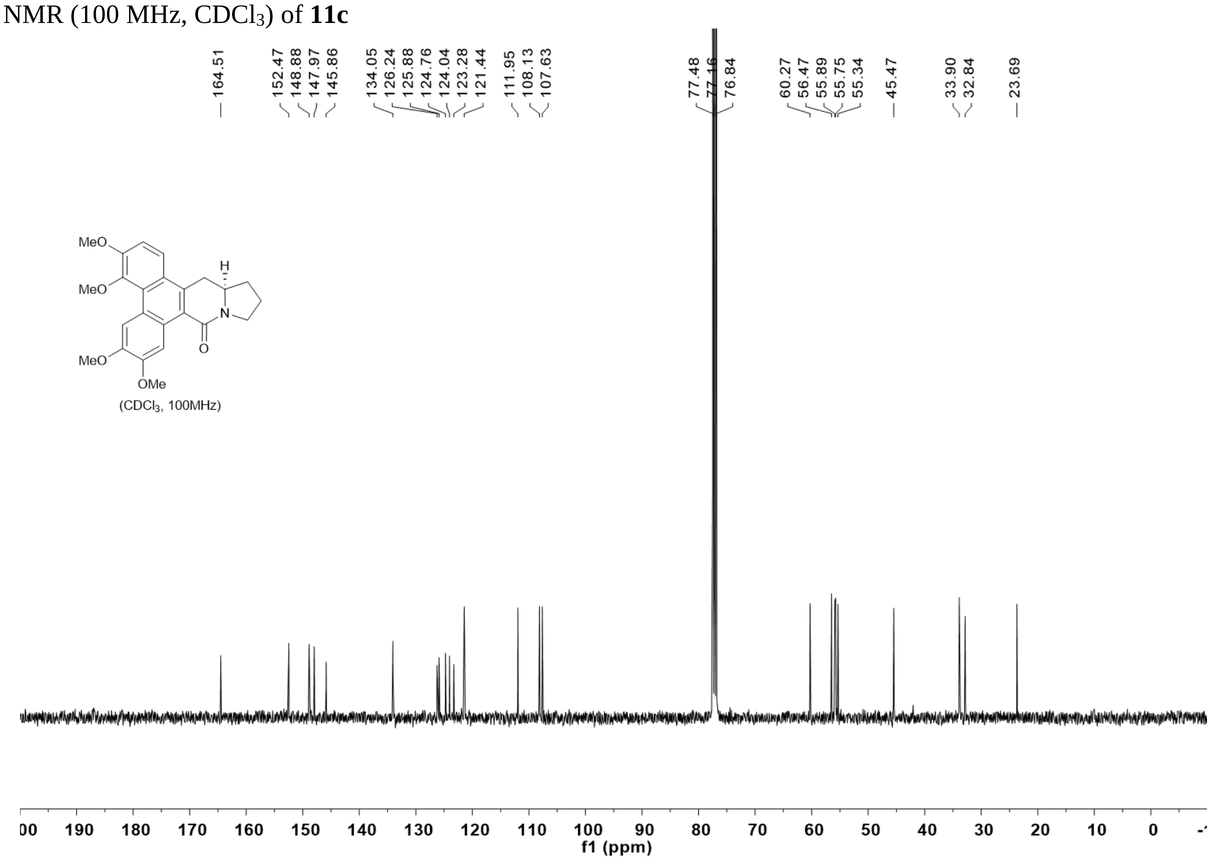

^{1}H NMR (400 MHz, $CDCl_3$) of **3c**

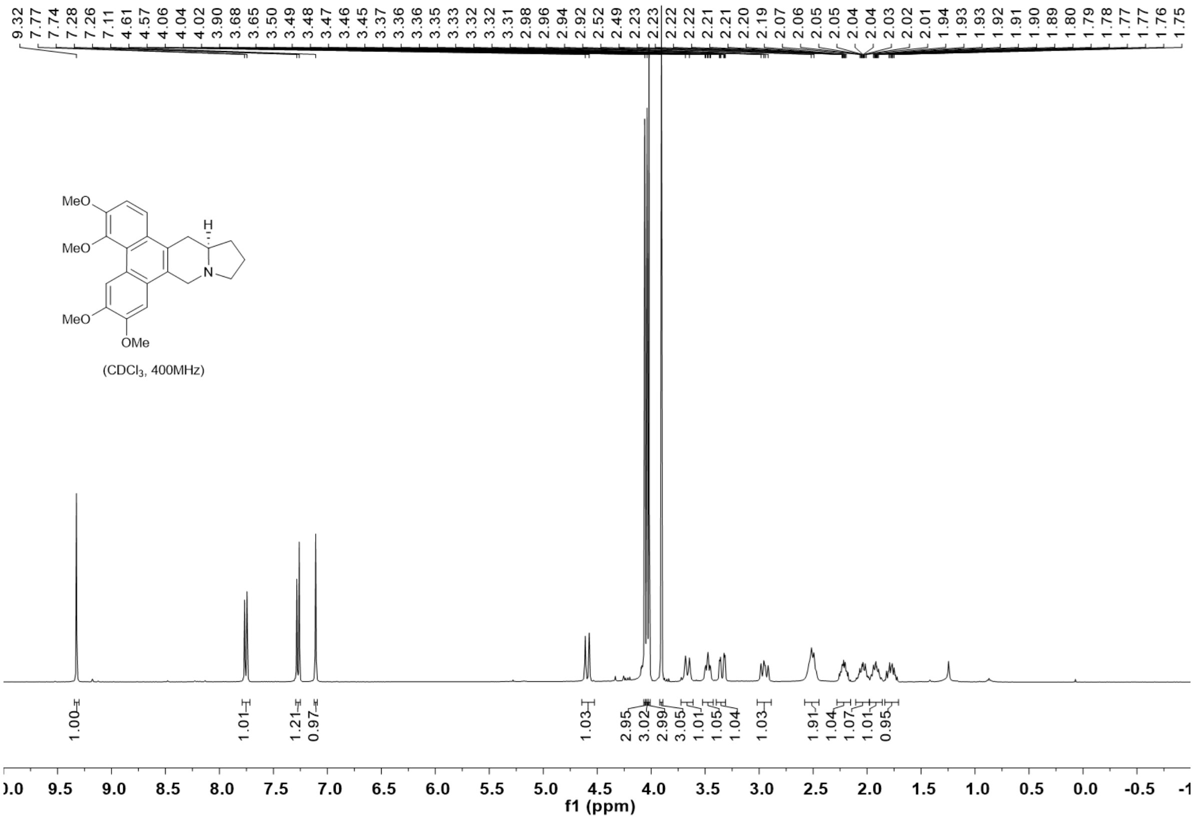

^{13}C NMR (100 MHz, $CDCl_3$) of **3c**

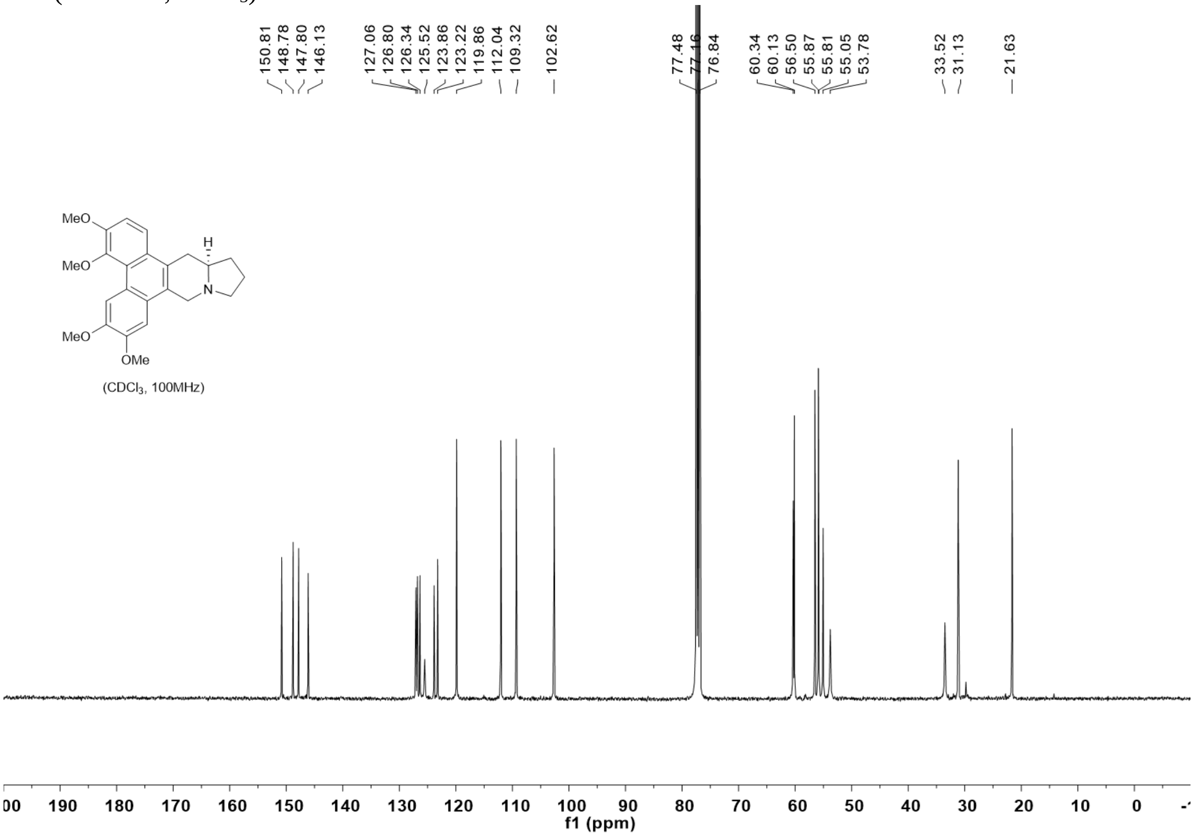

^{1}H NMR (400 MHz, $CDCl_3$) of **S1**

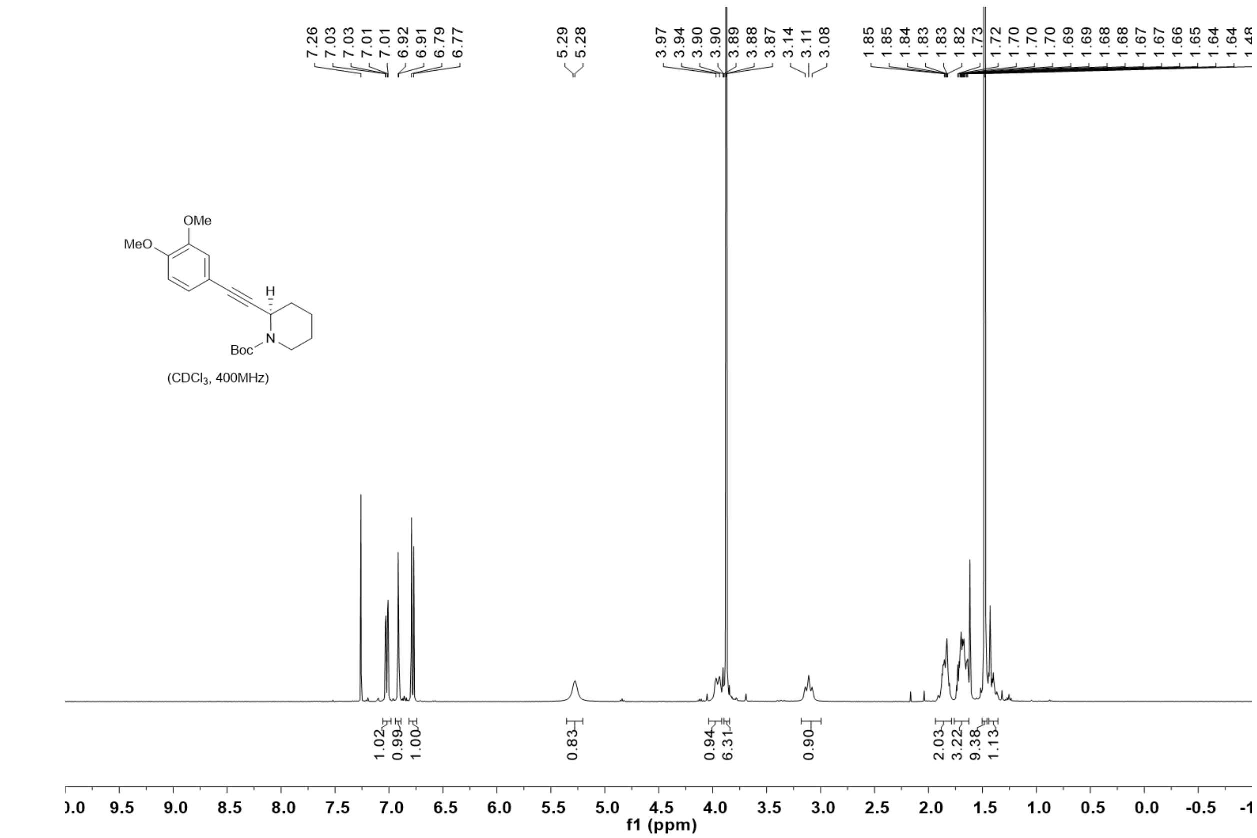

^{13}C NMR (100 MHz, $CDCl_3$) of **S1**

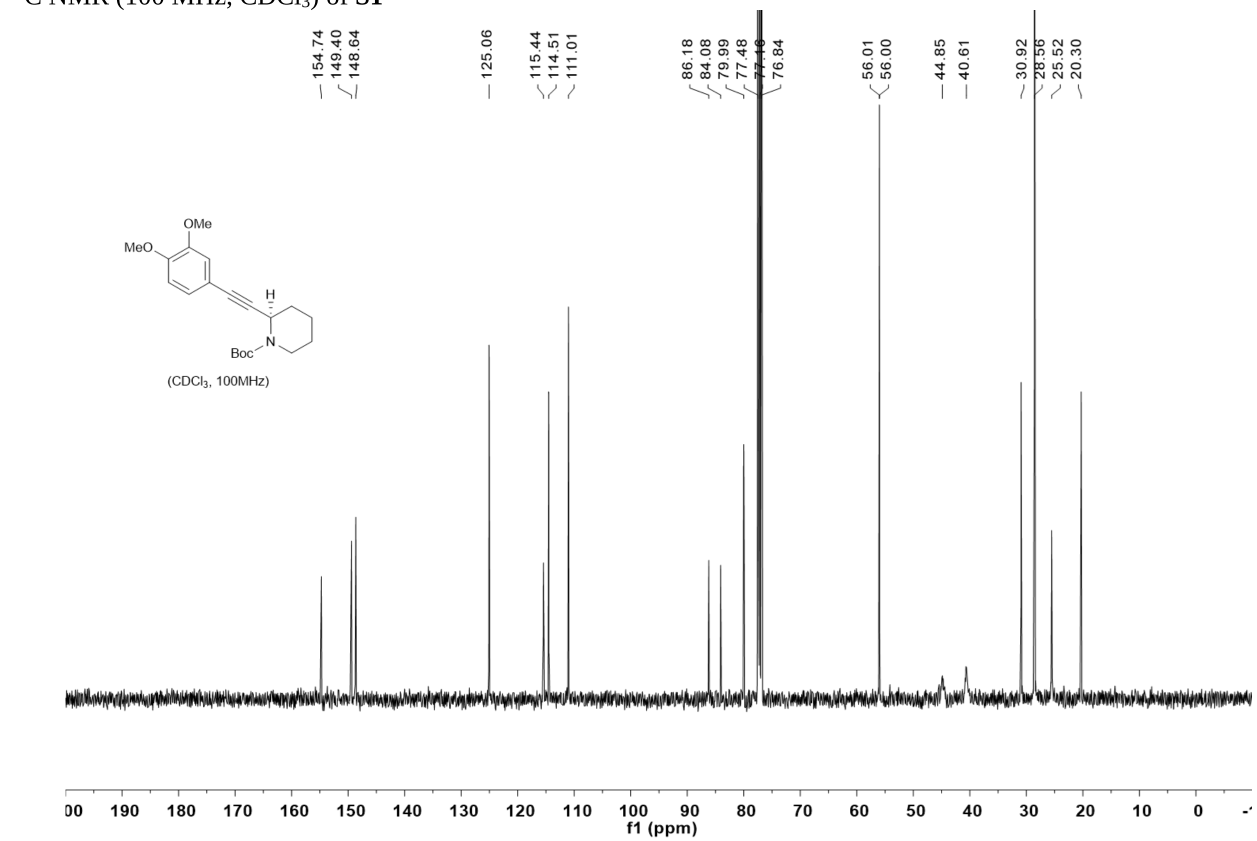

^{1}H NMR (400 MHz, $CDCl_3$) of **13**

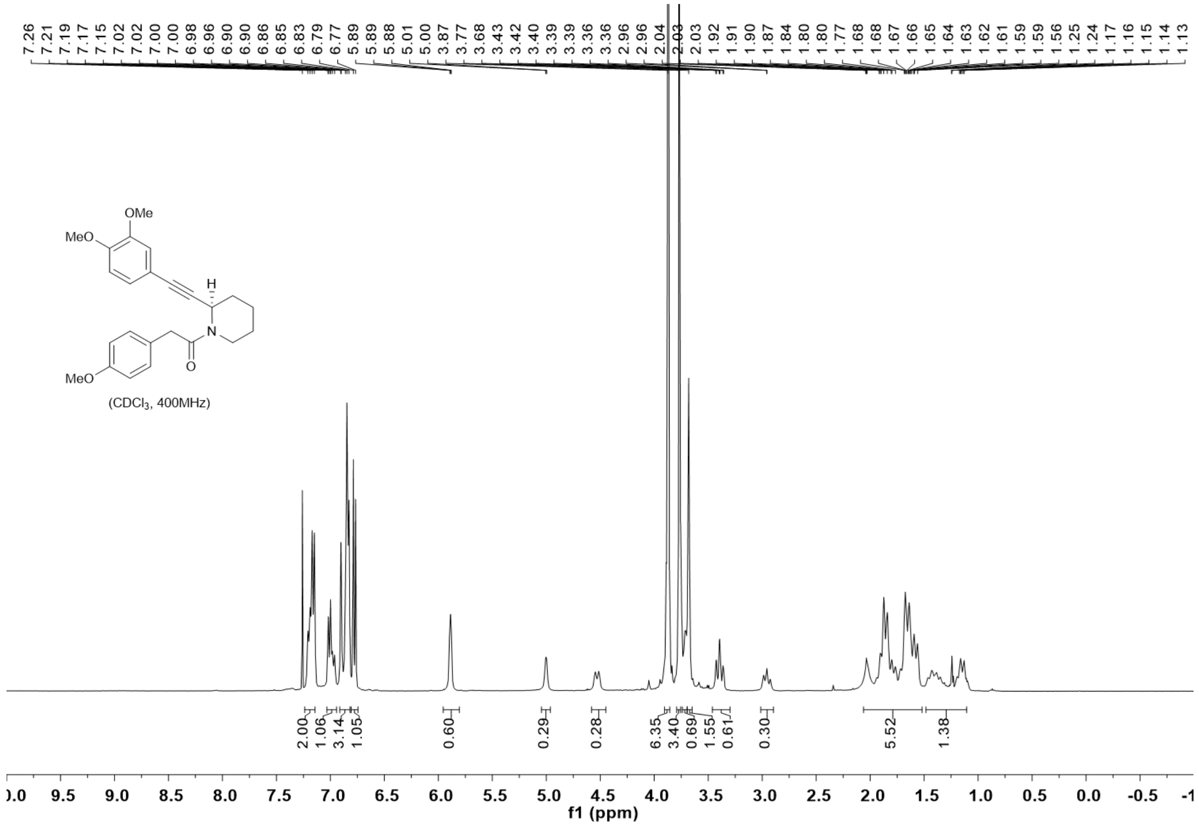

^{13}C NMR (100 MHz, $CDCl_3$) of **13**

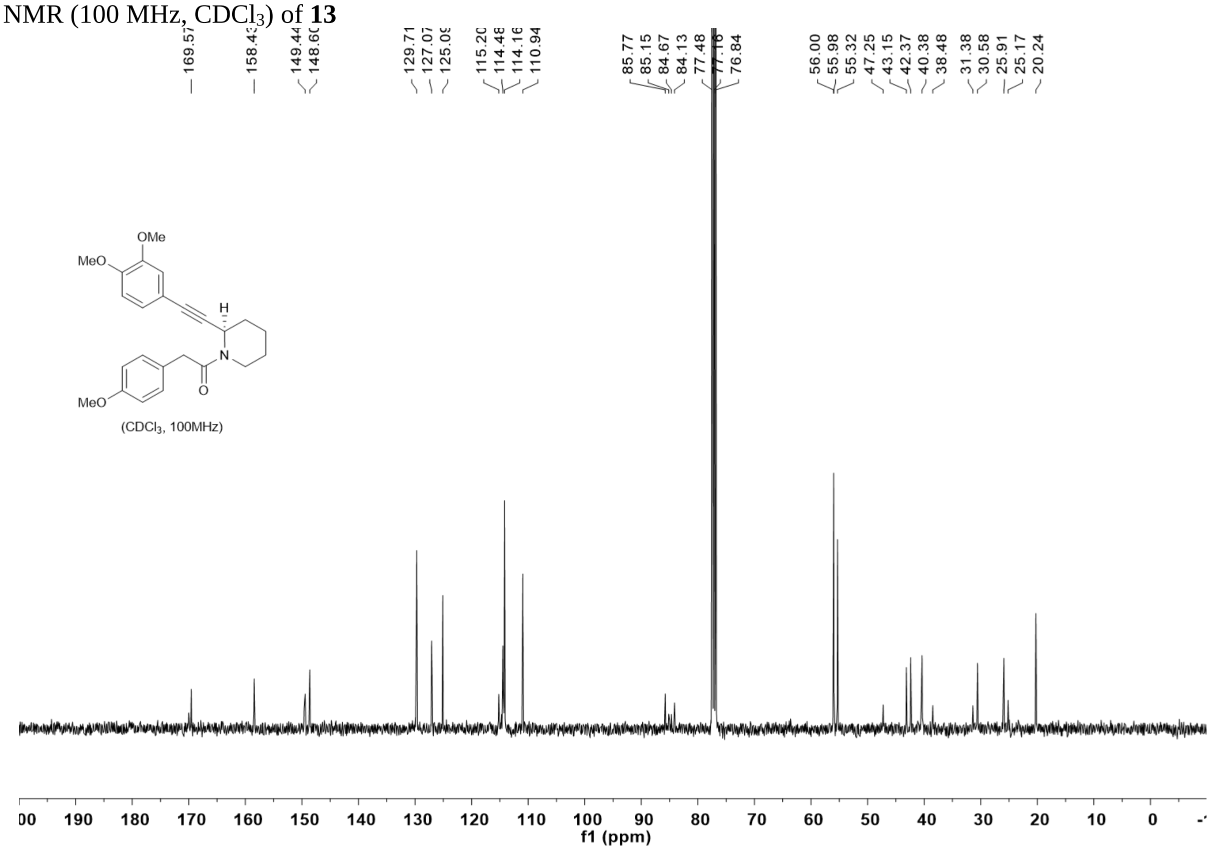

^{1}H NMR (400 MHz, $CDCl_3$) of **S2**

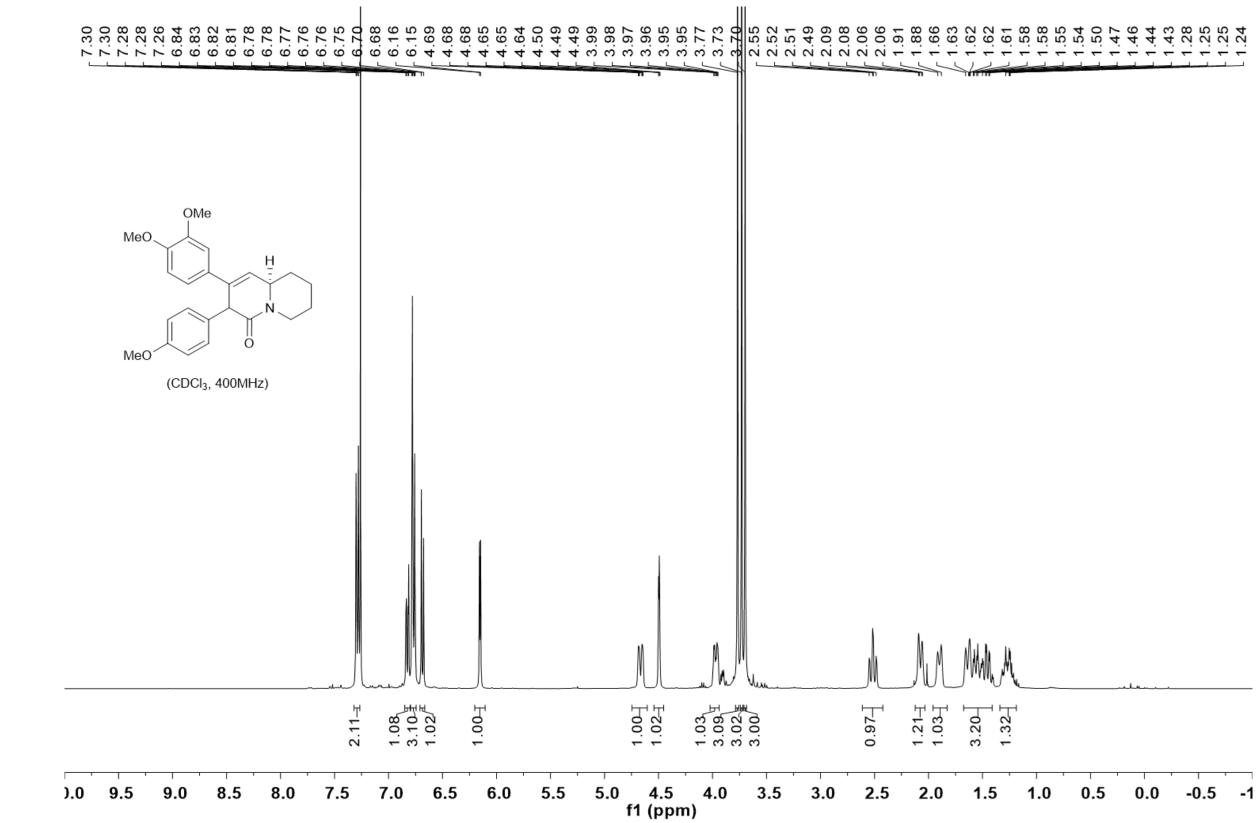

^{13}C NMR (100 MHz, $CDCl_3$) of **S2**

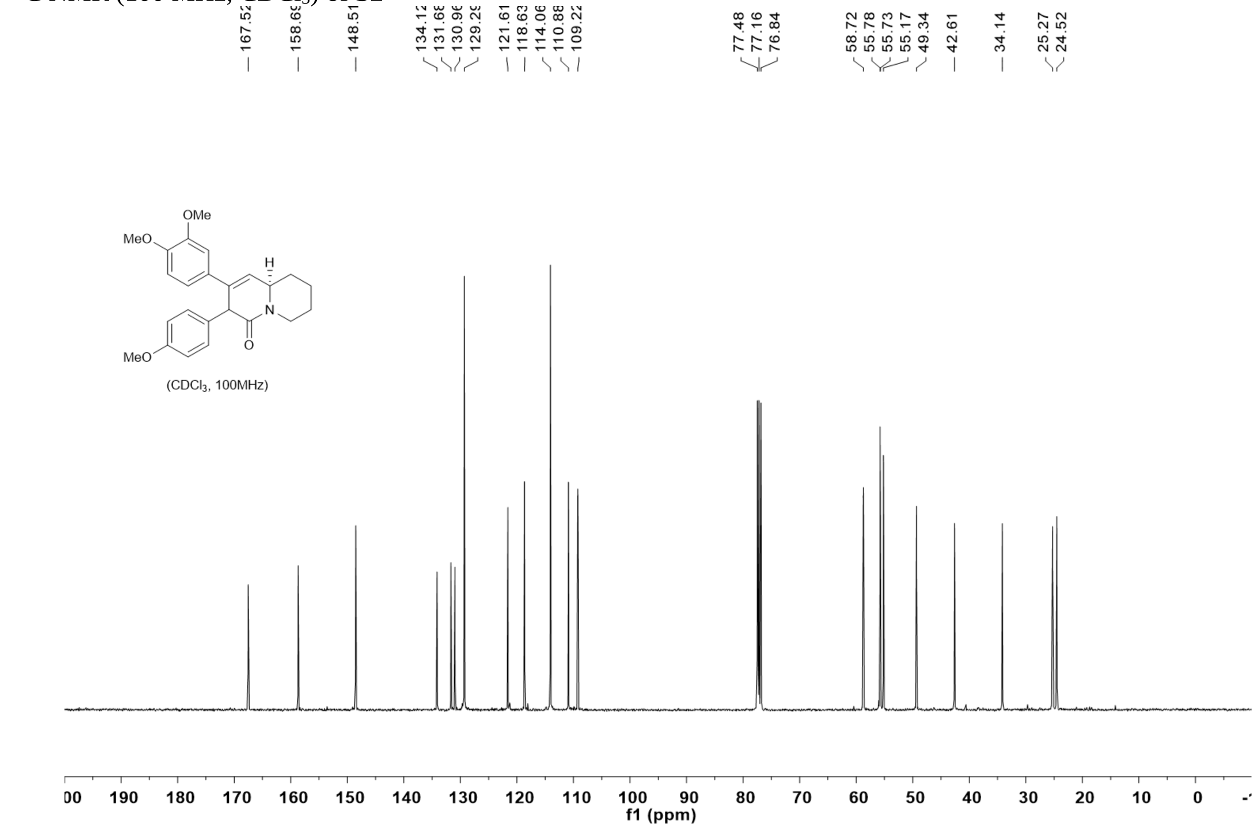

^{1}H NMR (400 MHz, $CDCl_3$) of **14**

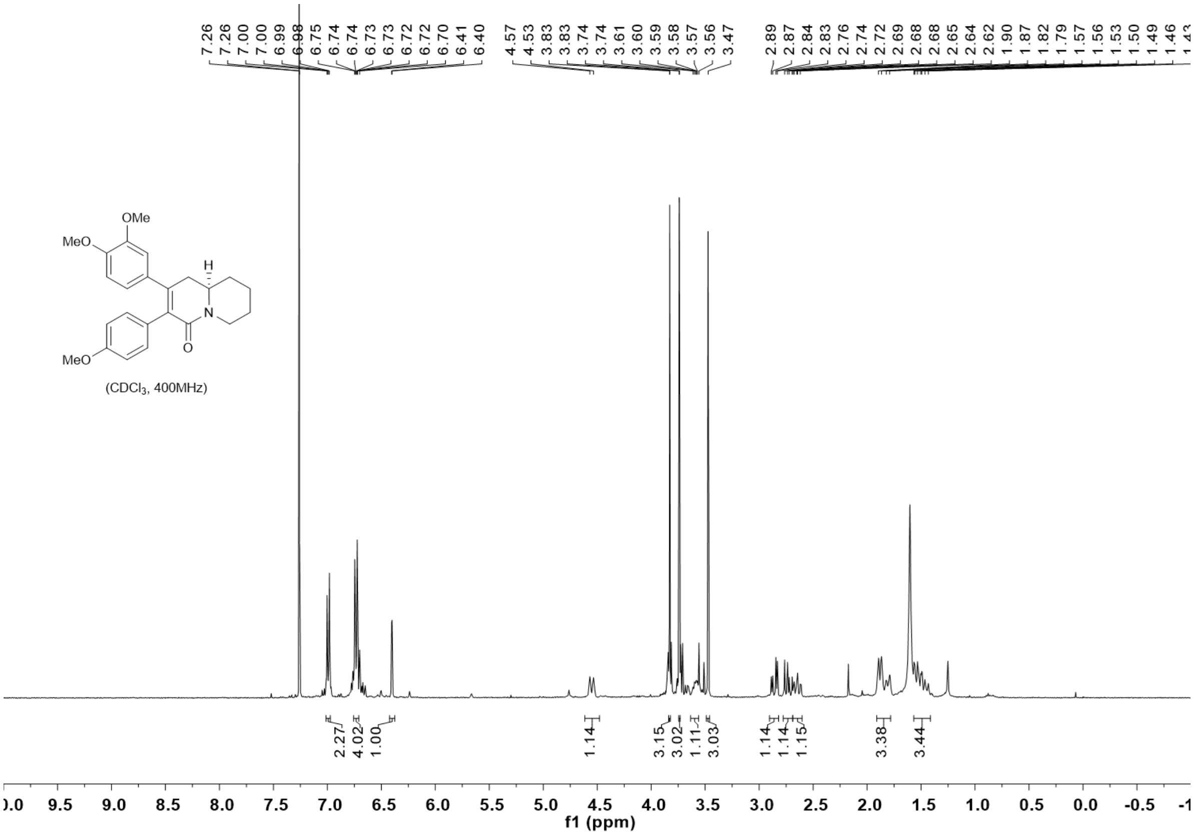

^{13}C NMR (100 MHz, $CDCl_3$) of **14**

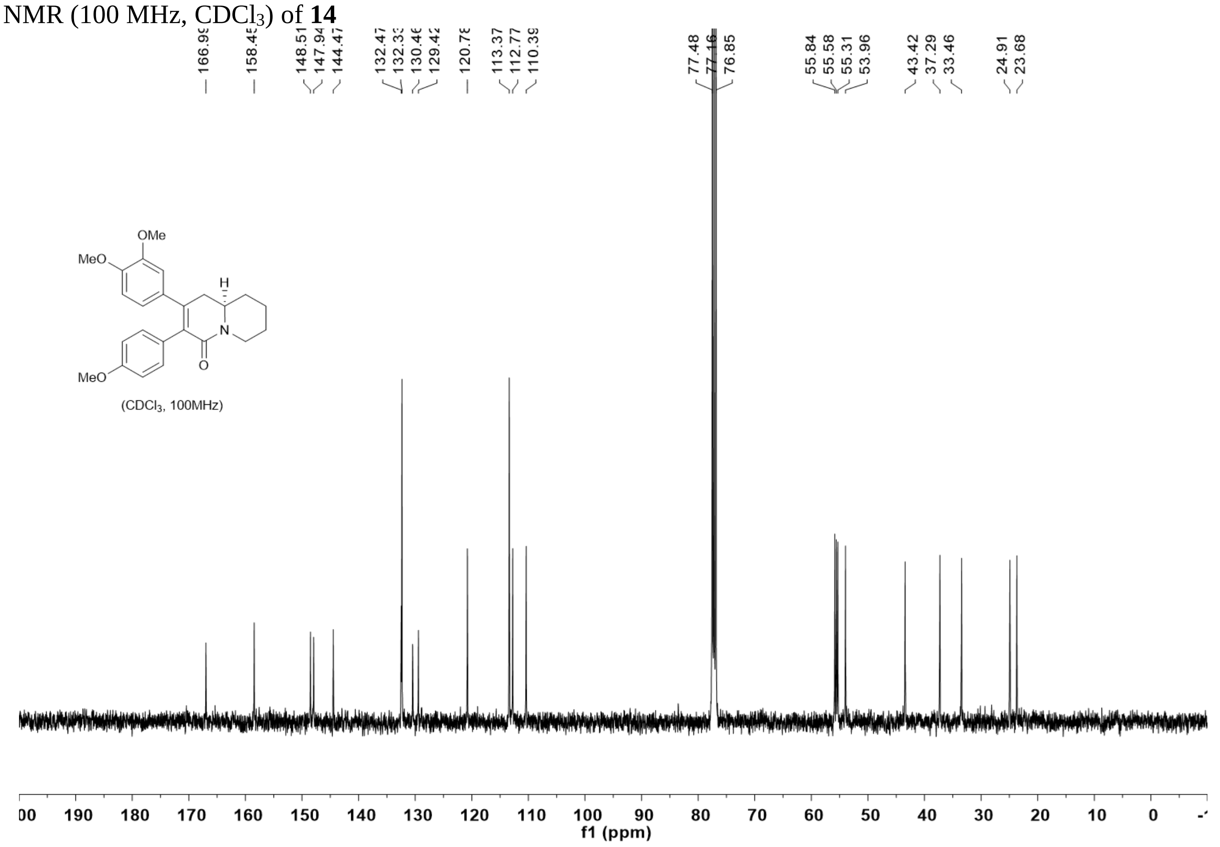

^{1}H NMR (400 MHz, $CDCl_3$) of **S3**

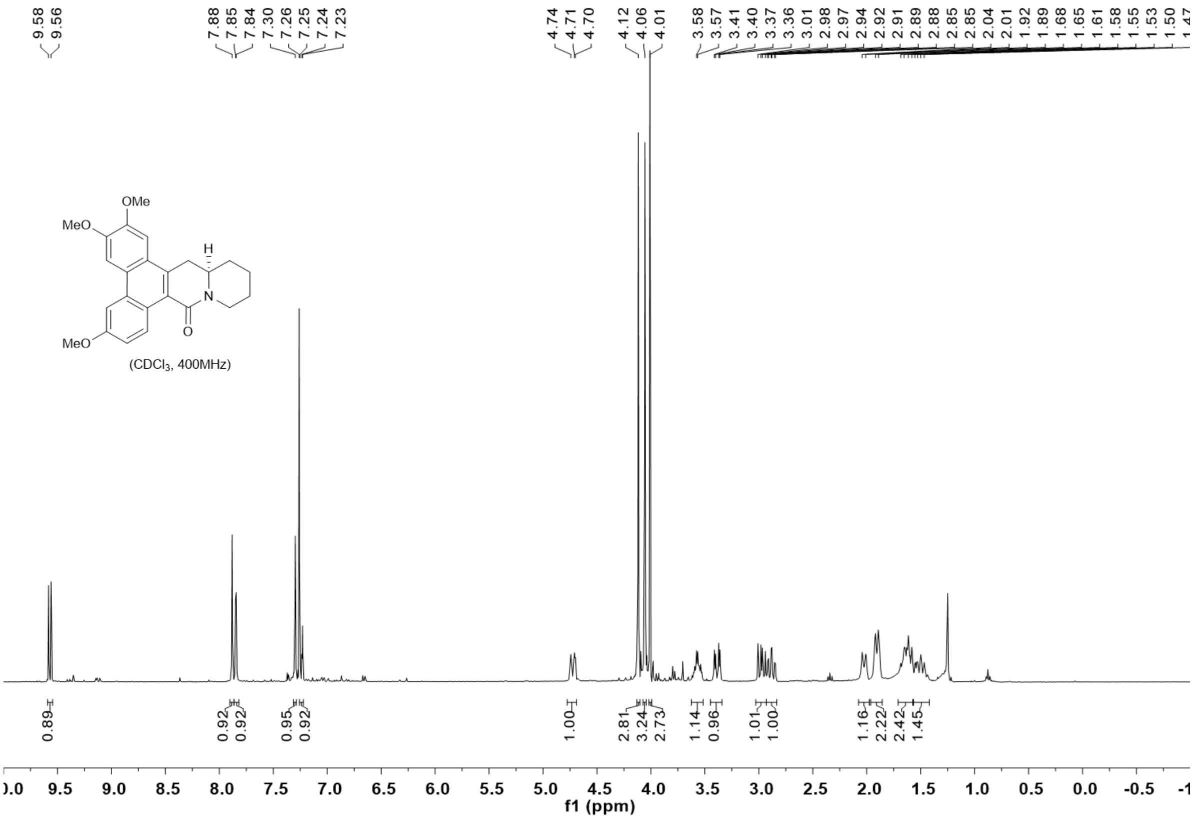

^{13}C NMR (100 MHz, $CDCl_3$) of **S3**

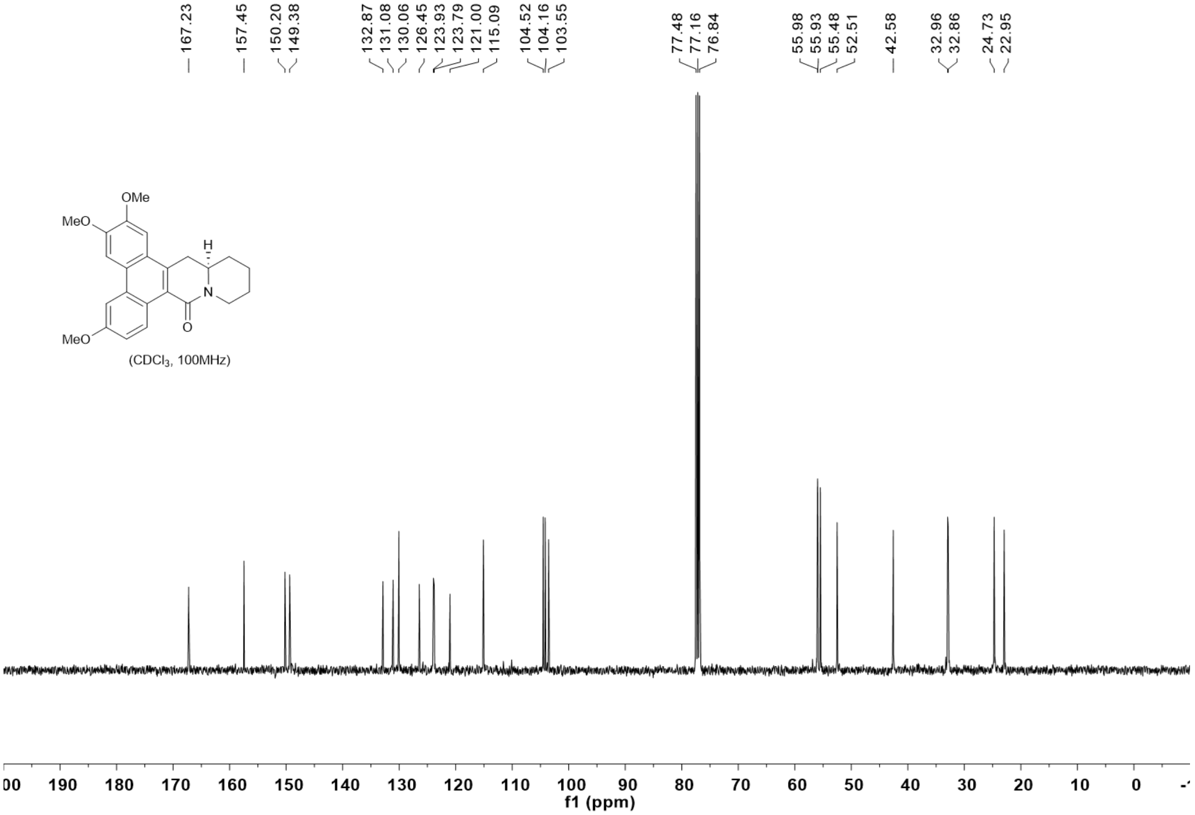

^{1}H NMR (400 MHz, $CDCl_3$) of **4a**

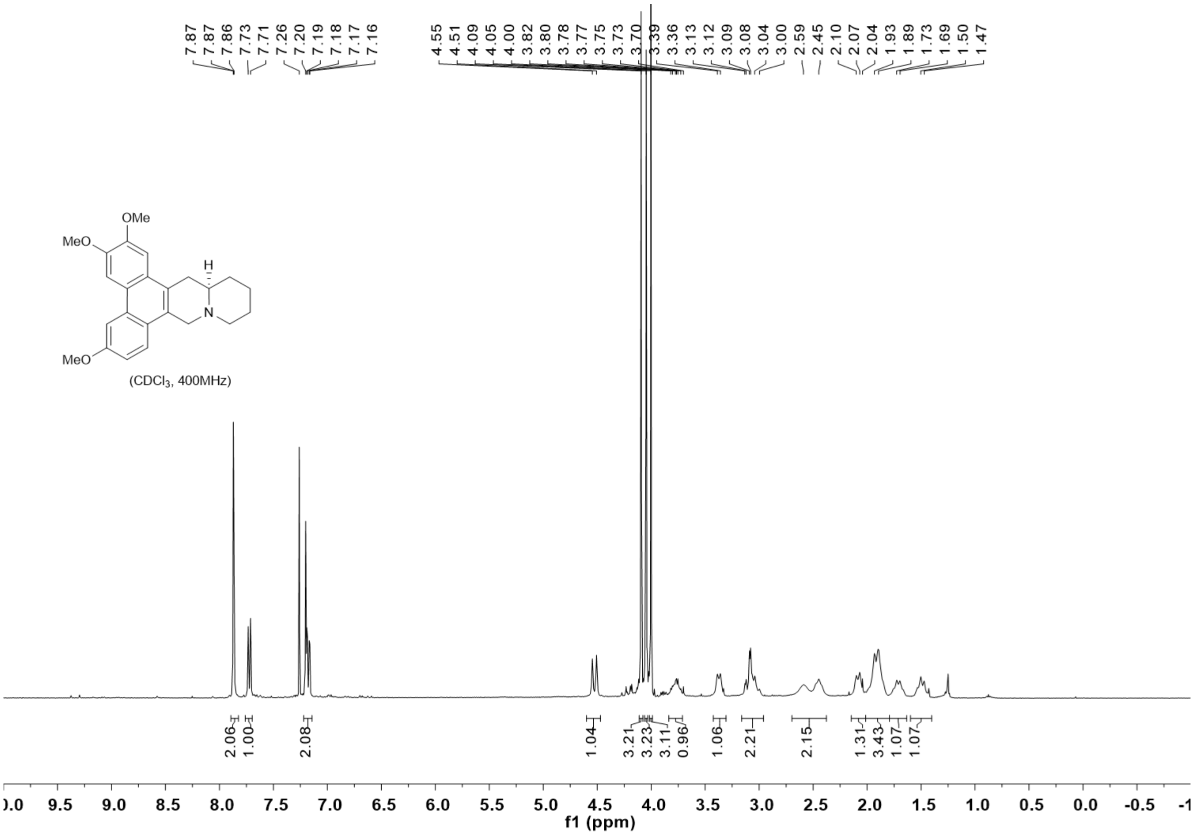

^{13}C NMR (100 MHz, $CDCl_3$) of **4a**

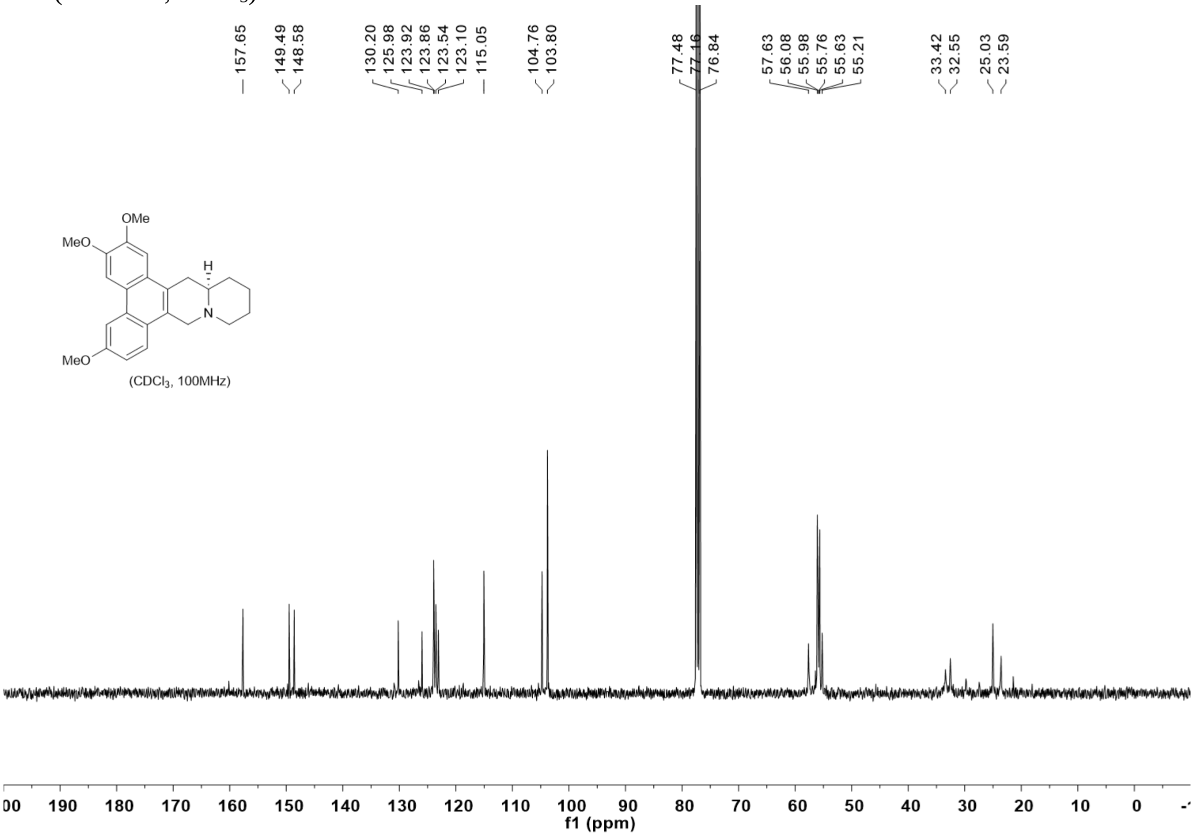

Supplement: Supplementary file 1 [file DataSheet1.pdf]
